# Inhibition of dimeric SARS-CoV-2 Mpro displays positive cooperativity and a mixture of covalent and non-covalent binding

**DOI:** 10.1016/j.isci.2025.112773

**Published:** 2025-05-28

**Authors:** Krishna M. Padmanabha Das, Jun Chen, Paul S. Charifson, Jeremy Green, Henry Tang, Sanjay Panchal, Fan Pu, Alla Korepanova, Abhinav Dubey, Gustavo Afanador, Vladimir Stojkovic, Boguslaw Nocek, Lance Bigelow, Sarah H. Stubbs, Robert A. Davey, David A. DeGoey, Haribabu Arthanari, Mark N. Namchuk

**Affiliations:** 1 Department of Cancer Biology, Dana-Farber Cancer Institute, Boston, MA 02115, USA; 2 Harvard Medical School Therapeutics Translator, Blavatnik Institute, Harvard Medical School, Boston, MA 02115, USA; 3 Research and Development, AbbVie Inc, 1 N Waukegan Road, North Chicago, IL 60064, USA; 4 Department of Biological Chemistry and Molecular Pharmacology, Blavatnik Institute, Harvard Medical School Boston, Boston, MA 02115, USA; 5 Department of Microbiology, Boston University School of Medicine and NEIDL, Boston University, Boston, MA 02118, USA

**Keywords:** Biochemistry, Immunology, Virology, Structural biology

## Abstract

SARS-CoV-2 Mpro is a cysteine protease that acts as a symmetrical dimer and displays positive cooperativity for substrate turnover. A series of potent reversible covalent peptidomimetic aldehydes and nitriles was designed as Mpro inhibitors. To better understand the observed structure activity relationships (SAR), binding potency and mechanism was examined by enzyme activity assay, surface plasmon resonance, X-ray crystallography, matrix-assisted laser desorption electrospray ionization, and nuclear magnetic resonance (NMR). Potent aldehydes bind Mpro cooperativity but bind covalently to only one subunit of the dimer. The analogous nitriles do not bind cooperatively, and the degree of covalent binding observed varied depending on the assay method employed. The NMR studies support that potent inhibition of Mpro by the nitriles does not require covalent binding. The data highlight the caveats in using orthogonal assays to confirm compound mechanism, particularly in cooperative systems.

## Highlights

• Binding of aldehydes and nitriles to Mpro homodimers drives a conformational change
• Binding to the Mpro subunits can differ and may not require a covalent interaction
• Aldehydes can bind cooperativity but only bind one Mpro subunit covalently
• Nitriles bind non-covalently by ^13^C NMR but covalently by MALDESI and X-ray

## Graphical abstract

**Figure:**
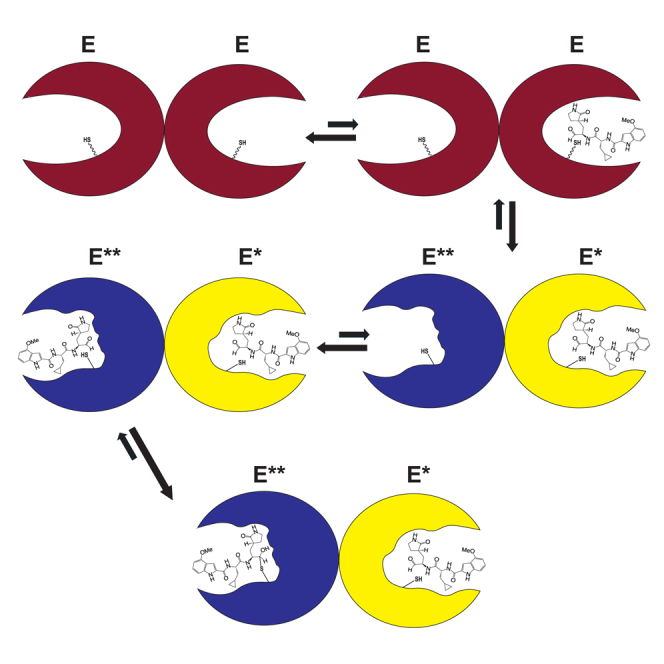

## Introduction

Covalent enzyme inhibition is a widely exploited strategy in drug discovery, in particular in the design of protease inhibitors. Protease inhibitors are a therapeutically important drug class with Food and Administration (FDA)-approved medicines in a variety of areas including diabetes,^1^ hypertension,^2^ cardiovascular disease,^3^ and viral infection.^4^^,^^5^^,^^6^ Cysteine and serine proteases affect bond cleavage through general-acid-/base-catalyzed nucleophilic attack of the target amide bond, forming a covalent enzyme-substrate intermediate followed by hydrolysis of the intermediate to release product and free enzyme. A common molecular design strategy for protease inhibitors is to employ a peptidomimetic scaffold exploiting the interactions that drive substrate recognition coupled to a “warhead” that engages the catalytic nucleophile reversibly or irreversibly. Warheads can include aldehydes, activated ketones, Michael acceptors, and nitriles.^7^^,^^8^^,^^9^ Activated ketones, aldehydes, and nitriles are thought to undergo at least a two-step binding mechanism, first binding the enzyme non-covalently, then undergoing nucleophilic attack to form a reversible, covalent complex. The strongest evidence to support covalent complex formation is often derived from X-ray crystallography studies.^6^^,^^8^^,^^10^^,^^11^^,^^12^

This peptidomimetic design strategy was employed to target the chymotrypsin-like or main protease (Mpro) of SARS-CoV-2.^13^^,^^14^ The first Mpro inhibitor to enter clinical development for treatment of COVID-19 was PF-07304814, which originated from work targeting SARS-CoV Mpro.^11^ PF-07304814 is a phosphate prodrug of the hydroxymethyl ketone PF-00835231. The cell permeability and bioavailability challenges associated with carbonyl bearing warheads have prompted a focus on more drug-like warheads such as nitriles. Nirmatrelvir, the Mpro inhibitor combined with ritonavir in Paxlovid, borrows critical structural features from the HCV protease inhibitor boceprevir, with the ketoamide warhead of boceprevir replaced with a nitrile.^6^ EDP-235 and ibuzatrelvir represent second-generation nitrile bearing peptidomimetic SARS-CoV-2 Mpro inhibitors.^15^^,^^16^

Studies of both substrate turnover and inhibitor binding to the SARS-CoV-2 Mpro have demonstrated a surprising level of mechanistic complexity. SARS-CoV-2 Mpro is a homodimer. Enzyme activity is greatly increased in the dimeric versus the monomeric form.^17^^,^^18^ In addition, substrate hydrolysis by the dimer SARS-CoV-2 Mpro shows positive cooperativity.^19^^,^^20^^,^^21^^,^^22^^,^^23^ Both the dimerization of the enzyme and generation of a catalytically competent conformation of the active site are supported by substrate or inhibitor binding.^24^ For example, Mpro monomers adopt an inactive structure characterized by an “unwound oxyanion loop” that rearranges to a conformation similar to active protein upon binding of covalent^24^ or non-covalent^25^ inhibitors.

The observation of cooperativity is supported by several studies where inhibitor or substrate engagement in one Mpro subunit can drive structural changes in the second subunit, even when unoccupied. Substoichiometric amounts of the aldehyde inhibitor GC373 increased Mpro catalytic activity.^26^ X-ray crystallographic studies by Lee et al. demonstrated that formation of the covalently bound, acyl enzyme intermediate for substrate cleavage drives conformational changes in the second, unoccupied Mpro subunit.^19^ Similarly, co-crystal structures obtained with SARS-CoV-2 Mpro and the aldehyde bearing proteosome inhibitor MG-132 show that inhibitor binding can occur at one subunit, with the other subunit unoccupied when the inhibitor is not added in stoichiometric excess to the protein.^27^ Additionally, incubation under crystallographic conditions that initially yielded crystals with both Mpro subunits occupied by MG-132 converted the structure to a form with only a single subunit occupied by MG-132.^27^ Another series of binding and structural studies were conducted with three Mpro substrates with inactive C145A, H41A, and SARS-CoV-2 Mpro.^23^ Here again significant structural rearrangements were noted for Mpro even when only one Mpro subunit was occupied by substrate.

Herein we present a series of biophysical studies conducted to understand the detailed inhibition mechanism of a peptidomimetic series of inhibitors of SARS-CoV-2 Mpro structurally similar to PF-00835231^11^ and GC-373.^21^ These studies were initiated to better understand differences in the structure activity relationships between analogous aldehydes and nitriles and specifically to determine if the contribution of covalent and non-covalent inhibition could be quantitated. For the tightest binding aldehyde bearing inhibitors in the study, we observed cooperative inhibitor binding, where the aldehydes bind both subunits of the dimer but only form a stable covalent complex on one of the two subunits. For the structurally analogous nitriles, cooperativity was not observed, and the degree of covalent bond formation observed varied by the method employed. Equilibrium binding studies conducted by nuclear magnetic resonance (NMR) suggested minimal covalent bond formation for the most potent nitrile assessed, whereas X-ray crystallographic studies and matrix-assisted laser desorption electrospray ionization (MALDESI) studies suggested significant covalent bond formation was occurring. These findings also provide additional details into the regulation of Mpro activity, the mechanism of compound binding as well as caveats for the use of orthogonal assays for confirmation of compound mechanism and inhibitor design for cooperative dimers.

## Results

### Potency determinations by inhibition of substrate hydrolysis and compound binding by SPR demonstrate inhibitor binding to Mpro is time-dependent and that SAR differs based on compound warhead

A series of approximately 150 compounds was synthesized, focusing on exploration of the SAR for warhead, P1, and P3 substitution on inhibition of SARS-CoV-2 Mpro, and herein we focus on a subset of matched molecular pair compounds that illustrate our findings. Exploration at P1 included substitution with a methyl sulfone that had been explored previously for the Rhino virus Mpro and would decrease hydrogen bond donor count versus the commonly employed γ-lactam.^28^ A clear trend in the SAR was the expected increase in potency when comparing compounds containing an aldehyde warhead to the structurally analogous nitrile. A stronger dependance on an optimal P1 substitution was observed for the nitriles over the aldehydes (see below). To better understand the drivers of the observed SAR, detailed biochemical studies were conducted on four matched molecular pairs contrasting the binding of aldehyde versus nitrile warheads, γ-lactam versus sulfone side chains at P1, and 4-methoxyindole versus carboxybenzoate (CBZ) at P3 (compounds 1–8, Table 1).

**Table 1:** Summary of biochemical and antiviral potency for compounds 1–9

Compound number and structure		SPR *N* = 3 (SD)				Kinetics *N* = 2 (95% CI)				Hill slope		EC_50_ SARS-CoV-2 (μM)
		kon (1/Msec)	koff (1/s)	T_1/2_ (Sec)	K_d_ (μM)	K_i_ (μM)		IC_50_ (μM)				
1		91547 (25,100)	1.00E-05	>69300	<0.00012	0.00041 (0.000298–0.000567)	0.00051 (0.000348–0.000756)	0.0027 (0.00211–0.00309)	0.0021 (0.00173–0.00243)	3.1	2.0	6.2
2		98100 (3340)	4.93E-5 (4.02E-05)	30511 (33768)	0.00051 (0.00042)	0.00025 (0.000177–0.000339)	0.00026 (0.000187–0.000357)	0.0016 (0.00138–0.00175)	0.0016 (0.00131–0.00170)	2.7	2.5	3.3
3		79900 (6150)	2.5E-4 (1.17E-04)	3421 (1920)	0.003 (0.0014)	0.0018 (0.00157–0.00215)	0.0018 (0.00157–0.00211)	0.0061 (0.00561–0.00643)	0.0058 (0.00546–0.00635)	1.5	1.5	ND
4		63800 (7300)	0.00089 (0.000056)	780 (50)	0.014 (0.003)	0.0057 (0.00431–0.00747)	0.0043 (0.00314–0.00605)	0.015 (0.0111–0.0206)	0.012 (0.00666–0.0158)	1.0	1.0	22
5		137367 (55200)	0.002548 (0.0019)	423 (339)	0.018 (0.10)	0.0043 (0.00351–0.00528)	0.0043 (0.00356–0.00509)	0.011 (0.00910–0.0134)	0.012 (0.00824–0.0129)	1.0	1	3.2
6		5437 (255)	0.00562 (0.00031)	124 (7)	1.0 (0.1)	0.22 (0.196–0.258)	0.25 (0.212–0.299)	0.47 (0.403–0.565)	0.52 (0.407–0.680)	1.1	1.0	13
7		1840 (594)	0.0161 (0.0048)	45 (13)	9.8 (4.1)	2.2 (1.64–3.10)	2.5 (2.10–3.05)	4.8 (0.403–0.565)	>12.5	0.6	ND	>50
8				No activity	No Activity	>12.5	>12.5	>12.5	>12.5	ND	ND	ND
9^a^		926333 (602000)	0.000821 (0.00036)	973 (447)	0.001 (0.00028)	0.00044 (0.000351–0.000546)	0.00042 (0.000341–0.000511)	0.0019 0.00173 to 0.0020)	0.0019 (0.00182–0.00204)	1.8	1.9	ND

Also see Figures S1 and S3.

a Compound 9 is PF-00835231.

Compound potency was initially determined using a fluorescence resonance energy transfer (FRET)-based kinetic assay optimized to allow for characterization of tight binding inhibitors.^22^ During assay development, we noted that the addition of higher amounts of citrate led to an up to 5.9-fold increase in catalytic efficiency and increased inhibitor potency.^22^ Positive cooperative behavior for substrate hydrolysis was observed at sub-nanomolar enzyme concentrations in buffers containing high levels of citrate, whereas lower salt conditions did not show cooperative substrate kinetics until the enzyme concentration was greater than 100 nM.^22^ The citrate-containing buffer was adopted as our standard screening condition, allowing compound potency to be assessed on the dimeric form of the enzyme at low enzyme concentrations (1 nM Mpro final concentration). Compounds were incubated with Mpro for 15 min, then the reaction was initiated by substrate addition and total signal measured after an additional 20 min and potencies determined as a 34-point dose response fit to the Morrison tight binding equation (see STAR Methods, Figure S1). Assessments were conducted in duplicate.

Compounds 1 and 2 (both containing aldehyde warheads and a γ-lactam at P1) displayed sub nanomolar K_i_ values (average values of 450 pM and 250 pM, respectively). Accounting for the hydration penalty,^29^ compounds 1 and 2 bind extremely potently, with a likely affinity as low as 10 pM. Across matched molecular pairs, the aldehyde warhead provided up to 1,000-fold greater potency versus the analogous nitrile. Substitution of γ-lactam for a sulfone at P1 led to a 10- to 20-fold decrease in potency for the aldehydes but up to a 750-fold decrease for the nitriles (compound 5 versus 7), suggesting a greater dependence on P1 for nitrile binding. Substitution of the P3 position resulted in a roughly 5-fold increase in potency when comparing the 4-methoxyindole to CBZ for both warheads.

Cellular EC_50_ values were determined for inhibition of *in vitro* replication of SARS-CoV-2 in A549 cells.^30^ The most potent molecules had EC_50_ values in the single-digit micromolar range (Table 1), a decrease of at least 23,000-fold versus the K_i_ determined in citrate buffer for compound 1 (the most potent aldehyde) and 760-fold for compound 5 (the most potent nitrile). Cell permeability issues are well known for peptidomimetics and likely in part contribute to the decreased cellular potency.

Inspection of the dose responses from the initial biochemical potency assessments suggested that the Hill slope might vary by compound, a factor not accounted for in the Morrison equation. The identical datasets were reanalyzed as four-parameter IC_50_s allowing for changes in Hill slope to fit the data (see STAR Methods). The three most potent compounds (compounds 1, 2, and 9) showed clear evidence of a Hill slope greater than 1, whereas the other compounds showed Hill slopes close to 1 (compound 3 showed a Hill slope of 1.5), suggesting the tightest binders might show a degree of cooperativity upon binding to Mpro, analogous to the effects seen with substrate (Table 1; Figure S1).

Simultaneous addition of inhibitor and substrate showed Mpro inhibition to be time dependent (Figure S2). Similar slow onset of inhibition of Mpro has been reported by others including for EDP-235.^16^^,^^24^^,^^31^ Several attempts were made to examine the pre-steady state kinetics of inhibitor binding under the citrate buffer conditions using the FRET assay but did not provide a reliable assessment of pre-steady state compound binding.

Compound binding kinetics were directly assessed via surface plasma resonance (SPR). In general, K_d_ values determined by SPR correlated well with the K_i_ values, though in most cases, the K_d_ values were less potent than K_i_ values (Table 1; Figure S3). The second order rate constants for compound association were low, well below the diffusion-controlled limit for small molecule binding.^32^ For the aldehydes, changes in compound potency largely correlated with off-rate. Compounds 3–9 showed clear evidence of reversible binding (Figure S3), whereas the more potent aldehydes formed a kinetically stable complex with Mpro, with compounds 1 and 2 having estimated half-lives of >69,300 s and 30,500 s respectively (Table 1). In contrast, matched compounds with nitrile warheads had half-lives of 400 s or less. Interestingly, the hydroxymethyl ketone PF-00835231 (compound 9) displayed remarkably different binding kinetics compared to compound 1 despite the structural similarity, with higher rates of association and dissociation (T_1/2_ > 69,300 s versus 973 s for compound 1 versus PF-00835231). For technical reasons, the SPR assessments were conducted in a lower salt buffer than the high citrate buffer used for the K_i_ determinations. Where possible, we conducted subsequent biophysical measurements in both buffers to control for the effect of buffer. Based on the relative compound potencies and differences in complex stability, we wanted to determine if the decreased potency observed with the nitriles was associated with a decrease in the extent of covalent binding. A series of studies was initiated to determine the degree to which covalent binding was a driver of inhibition for both the aldehydes and nitriles.

### X-ray crystallography was consistent with covalent bond formation for both the aldehyde- and nitrile-containing inhibitors

**Figure 1:**
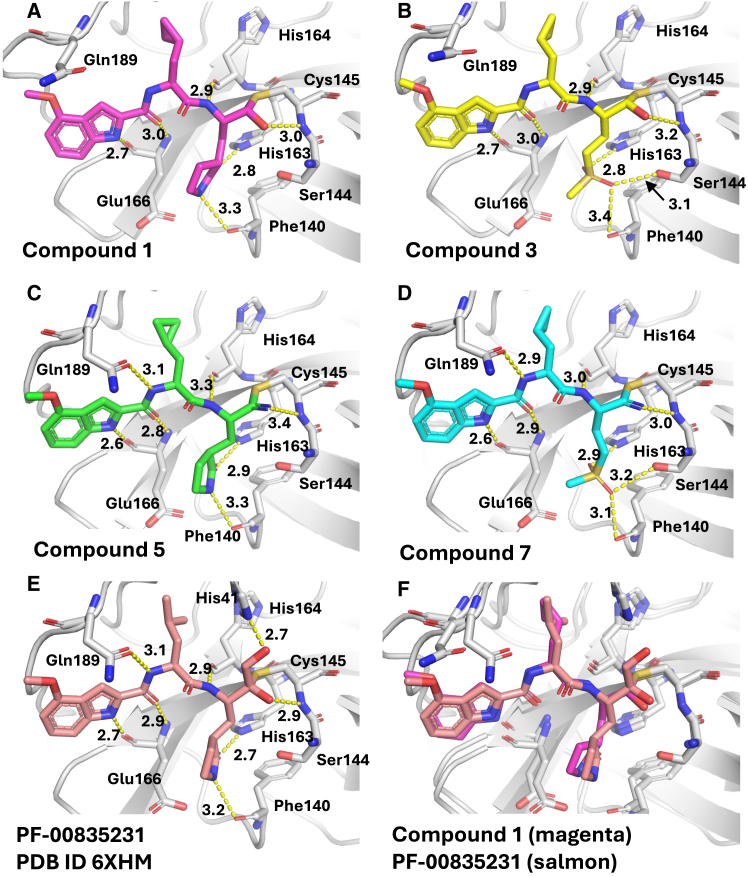
Crystal structures of SARS-CoV-2 Mpro in complex with the four 4-methoxy indoles in the compound set. The overall pose and interactions between the compounds are maintained with changes at the P1 and P3 positions (A–D) and show conservation with the literature compound PF-00835231 (compound 9; E), further reinforced by an overlay of PF-00835231 and compound 1 (F). Polar interactions are depicted by dashed yellow lines, and distances are indicated in angstroms. (Also see Figure S4*;*Table S1).

To examine the detailed binding mode of the compounds in the series, crystal structures were obtained of SARS-CoV-2 Mpro in complex with the 4-methoxyindole containing compounds 1, 3, 5, and 7 (Figure 1). The resolution of the structures ranged from 1.87–2.42 Å. The overall binding pose was similar across the four compounds and in line with what has previously been characterized by Hoffman et al.^11^ The P1 group inserts into the S1 pocket of Mpro, and the cyclopropyl moiety inserts into the S2 pocket, whereas the indole maintains its position across these analogs. All four compounds show a covalent linkage to the catalytic cysteine, Cys145, with contiguous electron density between the sulfur and carbon atoms and the C-S bond distance 1.76–1.77 Å (Figure S4). Small differences were observed in the orientation of the cyclopropyl group that inserts into the S2 pocket across the four structures, which is in line with the nonspecific van der Waals interactions located at this site. Finally, Gln189, which has been observed to interact with the amide nitrogen linking the indole on the compound to the rest of the molecule, appears in two rotameric states across our four structures, half of which do not interact with the amide.

The changes at the warhead and P1 positions of the molecule result in new interactions. For the unmodified portions of the molecule, sidechains in the active site remain in the same rotameric states across these structures with the exception of Gln189. At the warhead position, when the group is a nitrile (compounds 5 and 7), the resulting imine nitrogen, formed following covalent attack by Cys145 thiol, forms a hydrogen bond with the backbone nitrogen of Cys145. When the warhead is an aldehyde (compounds 1 and 3), the resulting secondary alcohol hydrogen bonds with the Cys145 backbone nitrogen. At P1, the installation of a methyl sulfone moiety at this position results in hydrogen bonding to the imidazole of His163 as in the case of the γ-lactam, whereas the other sulfone oxygen is hydrogen-bonded to the side-chain hydroxyl group of Ser144. No differences in ligand density between the two Mpro subunits were noted for any of the compounds assessed.

These studies showed remarkable conservation of binding mode, including a covalent interaction with Cys145, over a >1,000-fold difference in inhibitor potency, and no notable differences in the binding mode for compounds 1 and 9 versus the other compounds. Given the conditions required to obtain crystals may have influenced the extent of covalent bond formation, two additional biophysical methods were applied to directly determine the degree of covalent bond formation and its contribution to binding.

### MALDESI analysis demonstrates covalent bond formation is time dependent and incomplete for the aldehydes

**Figure 2:**
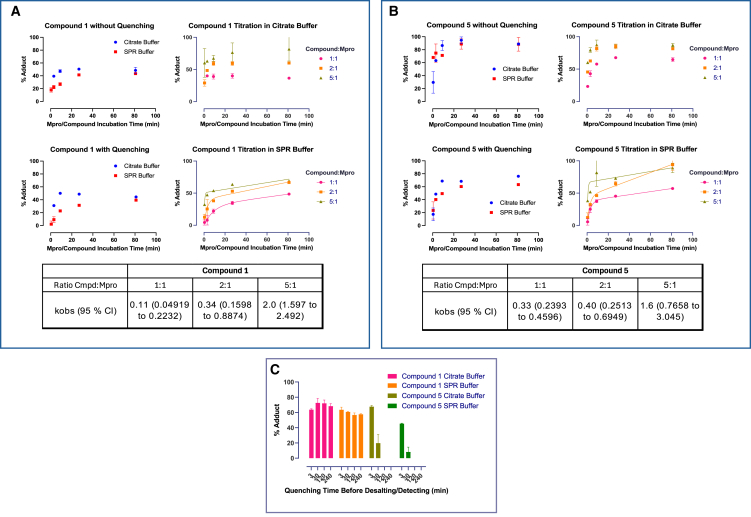
Time course for the degree of covalant bond formation and complex stability for compounds 1 and 5 measured by MALDESI Time course assessments of the degree of covalent adduct formation for compounds 1 (A) and 5 (B) by MALDESI. Time courses were conducted over 80 min of compound incubation time in both the citrate and SPR buffers. Each measurement was conducted five times and is presented as the average (SD) of the points where the percent adduct formation could be accurately calculated (see STAR Methods). Samples were obtained at the indicated time points and either desalted twice before the MALEDSI assessment (without quenching) or treated with 200 μM compound 9 to minimize rebinding of the compound (with quenching). The rate and extent of adduct formation was also assessed at varying molar ratios of compound to Mpro (1:1, 2:1, and 5:1) under both buffer conditions. The time course was best observed in the SPR buffer and fit to the slow tight binding equation (see STAR Methods) to obtain the k_obs_ for adduct formation (bottom tables, A and B). (C) Reversibility of adduct formation was assessed by incubating a 1:1 molar ratio of compound: Mpro then chasing with 200 μM compound 9 for up to four hours. (Also see Figure S5).

To directly examine the rate and extent of covalent bond formation, compound binding was examined by matrix-assisted laser desorption electrospray ionization (MALDESI).^33^ This technique allows for quantification of the amount of covalent bond formation, although it previously has only been applied to irreversible inhibitors.^33^ The six compounds with a K_i_ or K_d_ value less than 1 μM were analyzed. Studies were initially conducted using equimolar amounts of compound and Mpro (10 μM), and the extent of covalent bond formation was examined over a time course of 80 min. Studies were conducted both in the citrate and SPR buffers. Samples were prepared for MALDESI analysis in two ways. At each time point, the reaction mixture was subjected to two rounds of desalting (non-quenched condition) or the reaction was quenched by addition of 200 μM PF-00835231 to ensure any free compound could not rebind to the enzyme, then desalted (see STAR Methods). In both cases, samples were subjected to MALDESI analysis in under 5 min. Representative examples of the data obtained are shown in Figures 2 and S5. For all compounds tested, the extent of covalent complex formation was time dependent. The time course was best observed under the quenched conditions. The rate of covalent adduct formation was similar to the rate of onset of inhibition in the kinetic assay (Figures 2, S2, and S5). In alignment with the data obtained from the SPR studies, the rate of complex formation was similar across all compounds tested and did not correlate to compound affinity.

Although the rate of covalent complex formation was somewhat slower in SPR buffer, the extent of complex formation was the same after 80 min. For the four aldehydes tested, the extent of covalent complex formation was roughly 50% in both the quenched and non-quenched condition (Figures 2 and S5). We confirmed that the substoichiometric binding was not due to epimerization of the P1 group (Figure S6). For the two nitriles assessed, the extent of covalent bond formation was consistent with the relative potency of the compound, and quenching decreased occupancy. A titration was performed for the most potent aldehyde (compound 1) and nitrile (compound 5) (Figures 2A and 2B). Even at a 5-fold molar excess of compound 1, covalent complex formation did not reach saturation, whereas for compound 5 near 100% covalency was achieved in the unquenched condition (Figures 2A and 2B). The time course for complex formation in the dose response study was best observed in the SPR buffer conditions. The data were fit to the slow tight binding equation to determine k_obs_ for the rate of covalent bond formation (Figures 2 and S5). Interestingly, k_obs_ was similar for compounds 1 and 5, but was concentration dependent. Given the compound concentration far exceeds the K_i_ values for both compounds, it argues a much lower affinity interaction is driving the conversion of Mpro to a form capable of covalent binding.

Finally, the reversibility of binding of compounds 1 and 5 was assessed. Both compounds were preincubated for 27 min, then chased with 200 μM compound 9 for up to 4 h. Covalent binding of compound 5 was fully reversed within 1 h, whereas compound 1 binding was unchanged for at least 4 h (Figure 2C). These data are consistent with the half-life values determined for the same compounds by SPR and further supported that the incomplete covalent bond formation observed with compound 1 was not due to desalting or the quenching procedure.

### Protein-detected NMR studies confirm Mpro binding for compounds 1 and 5 and suggest 1:1 stoichiometry with compound 1, with at least two different binding modes

**Figure 3:**
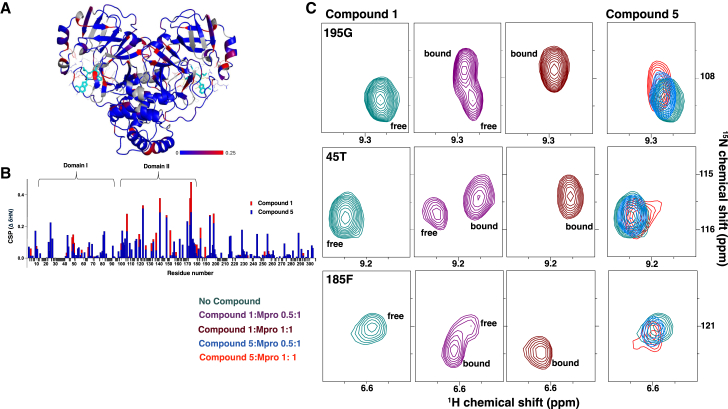
Protein-detected NMR using 15N-1H TROSY-HSQC to study the binding of compounds 1 and 5 to Mpro (A) The residues exhibiting significant CSPs upon addition of either compounds 1 or 5 were identified. The differences in CSPs between compounds 1 and 5 for each residue are mapped onto the crystal structure of the Mpro dimer, using a color gradient from blue (indicating no significant difference) to red (indicating a strong difference between compound 1 and compound 5). (B) CSP for compound 1 (red) and compound 5 (blue) compared to a DMSO control plotted as function residue number. Residues that could not be assigned are colored in gray in the crystal structure and are denoted as negative bars in the CSP plot. (C) Highlights from the ^15^N-^1^H TROSY-HSQC spectrum showing exemplar residues that exhibit significant differences in the exchange regime of binding between compounds 1 and 5. While compound 1 exhibits binding in the classical slow exchange regime, compound 5 binds in the fast exchange regime. (Also see Figure S7)*.*

To confirm the binding stoichiometry and compound association under equilibrium conditions, the binding of compounds 1 and 5 to Mpro was assessed by protein-detected NMR. Due to protein solubility issues, the NMR studies could only be conducted in the SPR buffer. Forty micromolar ^15^N-labeled and deuterated Mpro was incubated first with 20 μM compounds 1 and 5, then subsequently treated with an additional 20 μM compound to reach 1:1 ratio of Mpro to compound (40 μM final concentration). A number of residues showed large chemical shift perturbations (CSPs) upon compound binding including residues distant from the active site, indicating a substantial structural rearrangement in response to the compound (Figure 3). The backbone resonance assignments were transferred from the near-complete NMR assignments (97%) derived from a study on C145A variant of Mpro.^34^ Unfortunately, there were a few differences in chemical shifts when working with the wild-type Mpro, and active site residues such as Cys145 and His41 could not be resolved in the spectrum (Figure S7), but the assigned residues provided important mechanistic detail regarding compound binding. A marked difference in the extent of CSPs was observed for Mpro fully bound to compound 1 versus compound 5, with larger CSPs observed in the case of compound 1 (Figures 3A and 3B), indicative of larger conformational changes upon binding by compound 1. We mapped the CSPs for individual residues onto the PDB structure of Mpro (Figure 3A). The residues that experience significant CSPs are localized within domain I and domain II of Mpro that create both sides of the active site cleft (Figure 3A). CSPs due to compound 1 binding displayed a pattern typical of slow exchange or non-reversible binding where the signals for both the bound and free state are observed with signal integrals representing their relative amounts (Figure 3C). For the majority of the residues, equimolar amounts of Mpro and inhibitor were required to completely convert the signal from the free to the bound form (Figure 3C), supporting that both halves of the dimer are binding compound with 1:1 stoichiometry. Compound 5 displayed a pattern characteristic of rapid, reversible binding, in agreement with the data from the SPR and MALDESI experiments described above. Binding of the nitrile compound resulted in CSPs of relatively lesser magnitude, with peak shifts more characteristic of a fast exchange regime, where each peak represents the population-averaged value of the free and bound states (Figure 3C).^35^

**Figure 4:**
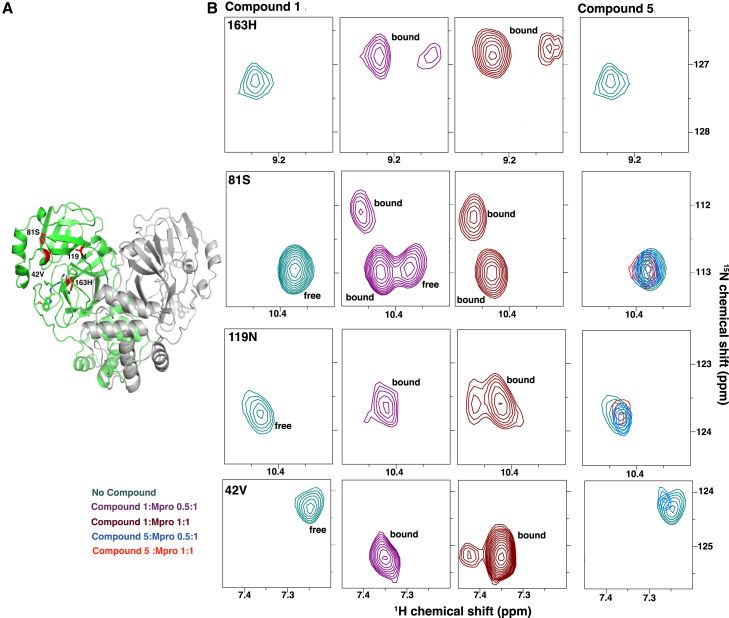
A subset of resonances exhibited two distinct signals for a given residue in the bound conformation at a 1:1 stoichiometry of Mpro to compound 1 (A) Residues displaying two bound conformations are mapped onto the Mpro structure (highlighted in red) bound to compound 5, indicating proximity to the active site. (B) Spectral regions corresponding to these residues show resonance doubling at 20 μM and 40 μM concentrations of compounds 1 and 5. ^1^H-^15^N TROSY-HSQC peaks reveal two distinct bound conformations for compound 1. Binding of compound 5 induces minor chemical shifts (e.g., residues S81 and N119) or results in peak disappearance or decreased intensity (e.g., residues S163 and V42).

Four assignable residues (His163, Asn119, Ser81, and Val42) showed evidence of two new resonances after binding compound 1, suggesting that there may be more than one Mpro conformer when compound 1 is bound (Figure 4). These residues were again located in domains 1 and 2, flanking the compound binding site, but were not residues identified as making direct contact with the inhibitor in the crystal structures. Taken together, these data were most consistent with compound 1 forming a 1:1 stable complex with Mpro but that there may be two or more bound forms of the compound 1:Mpro complex. These same residues did not show two bound populations for compound 5 (Figure 4), suggesting the diversity in bound forms may be specific to the aldehyde. One possibility suggested by the MALDESI studies would be that the aldehyde is bound to the two-halves of the dimer differently, one-half covalently and the other non-covalently, whereas the binding mode for the nitrile was similar on both halves of the dimer.

### ^13^C-labeled compound binding demonstrated covalent and non-covalent binding for compound 1 and non-covalent binding for compound 5

**Figure 5:**
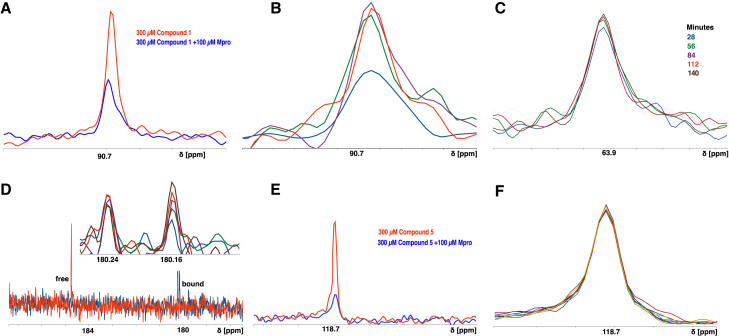
Ligand-detected ^13^C NMR studies to monitor the binding of compounds 1 and 5 to Mpro For both compound 1 and compound 5, a decrease in total signal intensity due to line broadening was observed at the earliest measurable time point (5 min), as shown in (A and E). The hydrated aldehyde peak at 90 ppm increased over time, likely indicating stable complex formation and a slow compound exchange rate on the NMR timescale (B). Compound 1 displayed a time-dependent increase in a peak at 64 ppm, consistent with thiohemiacetal formation upon attack of Cys145 (C). Additionally, two minor peaks emerged at 180 ppm, with chemical shifts consistent with a bound aldehyde (D); the upfield peak increased in intensity over time (inset). Compound 5 was incubated with Mpro for up to 6 h. While line broadening was evident at the earliest time point (E), consistent with binding, no new peaks emerged to suggest thioimidate formation, and no change in intensity was observed for the peak corresponding to the bound nitrile (F). (Also see Figures S8–S10).

NMR studies using ^13^C-labeled inhibitors can provide direct evidence for formation of a covalent enzyme inhibitor complex by examining the chemical shift observed upon rehybridization of the warhead carbon upon inhibitor binding.^36^^,^^37^^,^^38^^,^^39^^,^^40^ To examine the rate and extent of covalent binding under equilibrium conditions, labeled versions of compounds 1 and 5 were synthesized with ^13^C incorporated on the aldehyde carbon of compound 1 and nitrile carbon of compound 5 (Figure S8). Binding of the compounds to Mpro was examined by proton-decoupled ^13^C NMR where binding could be detected by line broadening and covalent binding by chemical shift changes upon rehybridization (SP2-SP3 and SP-SP2 for compounds 1 and 5, respectively).^36^^,^^37^^,^^38^^,^^39^ Compound 1 (300 μM) was mixed with Mpro (100 μM), and binding was followed for 3 h, with spectra collected at roughly 30-min intervals. In the absence of Mpro, compound 1 largely converts to the expected hydrate observed as a single ^13^C peak at 90.7 PPM (Figures 4B and S9) with a minor peak observed at 184.5 PPM corresponding to the free aldehyde (Figures 4D and S9). Addition of Mpro caused a rapid decrease in the peak corresponding to the hydrated aldehyde, consistent with line broadening associated with initial binding of compound 1 as well as loss of the free aldehyde peak. Over time, four peaks increased in intensity. The peak for the hydrated form of the unbound inhibitor at 90.7 PPM was observed at 28 min, and maximal peak intensity was reached by 56 min (Figure 5B). Given the observed slow exchange binding on the NMR timescale for compound 1, the data suggest that the kinetically stable, bound species form over an hour, a time course similar to the other studies. A second peak was observed at the 28-min time point at 63.9 PPM, consistent with the thiohemiacetal expected upon covalent compound binding (Figures 5C and S10). A small increase in signal for the peak was observed at between 28 and 56 min, hence the formation of the covalent intermediate seemed to proceed more quickly than stabilization of the signal intensity for the hydrated aldehyde. In addition, two minor peaks were observed to increase upon Mpro addition at 180.16 and 180.24 PPM consistent with two non-covalently bound forms of the aldehyde (Figures 5D and S9). For the peak at 180.16, signal again increased over time and was still increasing at the 122 min time point. This further supports that even after extended incubation, a portion of compound 1 is stably bound to Mpro non-covalently.

In contrast, while line broadening was observed at all time points measured upon addition of Mpro to ^13^C-labeled compound 5, peak intensity for the free nitrile did not change over time, and no new peaks were observed to support thioimidate formation and covalent binding even after 6 h of incubation (Figures 5E and 5F). These data suggest that under equilibrium conditions the nitrile does not form a significant amount of the covalently bound inhibitor. The lack of covalent bond formation may in part explain the larger CSPs and structural changes upon binding compound 1.

## Discussion

K_i_/IC_50_ determinations for the most potent inhibitors assessed in this study suggested binding may be cooperative for the two most potent aldehyde inhibitors but not for the less potent analogous nitriles. For all assessments conducted, inhibitor binding was time dependent. The time dependence was remarkably consistent across assays and was observed with Mpro concentrations ranging from 1 nM to 100 μM. This suggests these are properties of dimeric Mpro and not principally driven by enzyme dimerization under the conditions tested. For the aldehyde inhibitors, this time course was similar to the rate of covalent binding, but both the NMR studies supported multiple bound forms of the compound Mpro complex.

**Figure 6:**
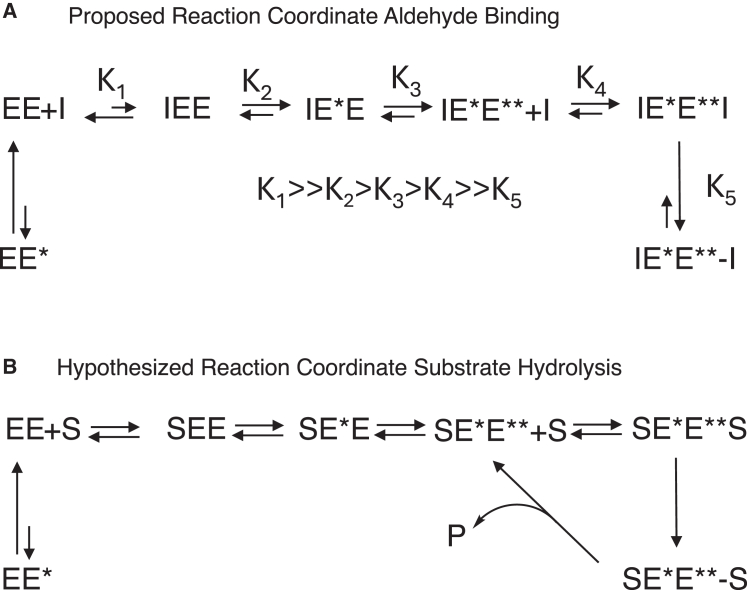
Proposed mechanism for binding of potent aldehydes and for substrate hydrolysis for dimeric SARS-CoV-2 Mpro A proposed reaction coordinate for cooperative binding of the potent aldehydes (A) and substrate hydrolysis (B).

A mechanism for the binding of aldehyde inhibitors to dimeric Mpro is proposed in Figure 6A using terminology similar to Nashed at al.^24^ In the absence of ligand, the dimer is present as an inactive dimer (EE). Compound binding to EE is weak (K_1_) but required to drive conversion of the bound monomer to a higher affinity complex, IE^∗^E (K_2_). Engagement of the first half of the dimer induces a conformational change in the unoccupied subunit that increases the catalytic efficiency of that subunit, IE^∗^E^∗∗^ (K_3_), and subsequently non-covalently binds inhibitor to form IE^∗^E^∗∗^I. The more catalytically active subunit is better aligned to drive covalent bond formation, thus driving accumulation of the IE^∗^E^∗∗^-I (K_5_), which for the more potent aldehydes is kinetically irreversible.

We hypothesize that the time dependence observed for binding is principally driven by the low abundance of IEE and the time required for this complex to equilibrate to the more thermodynamically favored and inhibitor-bound forms of E^∗^ and E^∗∗^. Both E^∗^ and E^∗∗^ can bind to compound covalently, but the relative affinity and extent of covalent bond formation varies by inhibitor and is highly dependent on warhead. Cooperativity occurs when the affinity of the compound is higher for E^∗∗^ than E^∗^ and in this study correlated with covalent binding to E^∗∗^ as observed with the most potent aldehydes. This mechanism is consistent with the positive cooperativity for substrate turnover and could explain the negative cooperativity observed for substrate binding in previous studies.^23^ A related phenomenon can occur where inhibitors of a target initially cause an increase in catalytic activity, by binding to one of the two dimer subunits, increasing the catalytic efficiency of the unoccupied subunit. Enzymes displaying this phenomenon include the GCN2 kinase,^41^ the cysteine protease caspase 1,^42^ the MERS Mpro,^20^ and truncated constructs of SARS-CoV-2 Mpro.^26^

The mechanism of the nitrile-bearing inhibitors of Mpro is less clear but also can be accommodated within the mechanism proposed in Figure 6A. X-ray crystallographic studies demonstrated covalent binding, even for the nitriles with micromolar affinity, whereas no covalent binding was observed in the ^13^C NMR study for the most potent nitrile, compound 5. The CSP data from the ^15^N NMR studies do support a robust shift in the Mpro conformation upon compound binding, corresponding with the formation of the IE^∗^E^∗∗^I complex in our model. The time dependence observed in both the enzyme kinetic studies and MALDESI assessments also support that the nitrile-based inhibitors induce a conformational change and binding to EE^∗^ is favored. The SPR studies, the MALDESI reversibility study, and NMR studies all support that compound 5 has a shorter residence time on Mpro and hence it is in rapid exchange between the bound forms and free enzyme. In the absence of quenching, compound 5 can achieve nearly 100% covalent complex formation measured by MALDESI, which suggests the bound covalent and non-covalent forms of the inhibitor complex are also in rapid exchange. A possibility is that the covalent and non-covalent forms of bound compound 5 have similar affinity (K_4_∼K_5_) consistent with the lack of cooperative binding and may be related to less avid binding of the thioimidate to the oxyanion hole than the analogous aldehydes. The NMR studies do support that compound 5 can bind Mpro without engaging the enzyme covalently. The lack of covalent binding offers a plausible explanation for the observed SAR where nitriles showed a greater dependance on the substitution of P1 versus the analogous aldehydes despite the similarity in the binding mode observed in the X-ray crystallography studies where all compounds were bound covalently. We note that both covalent and non-covalent Mpro inhibitors are viable antivirals^43^ and hence believe these studies such as those conducted herein principally aid in compound molecular design, but the mechanism in a more relevant cellular context is difficult to determine.

An extrapolation of these findings to regulation of Mpro enzymatic activity is shown in Figure 6B. When Mpro is present as a dimer, in the absence of substrate or inhibitor, it is predominantly in a low-activity form (EE). Upon substrate engagement, one monomer forms the SE^∗^E and subsequently SE^∗^E^∗∗^. Consistent with the cooperativity displayed for substrate hydrolysis, substrate turnover is largely driven by the more active E^∗∗^ subunit. Given that the rate of substrate hydrolysis does not increase with time (Figure S2), this suggests that the conversion to the E^∗∗^ in the presence of substrate is rapid on the timescale of substrate turnover. After substrate hydrolysis and product release, the dimer has only one subunit occupied and reverts to SE^∗^E^∗∗^. At steady state, the relative amount of E^∗^E^∗∗^ vs. E^∗^E will be dictated by the rate of substrate turnover and rate of interconversion of E^∗^E^∗∗^ back to E^∗^E and EE. Based on the data discussed above, we suggest that the abundance of E^∗^E^∗∗^ is low and that the activity of E^∗^E^∗∗^ is underestimated by the steady state kinetic analysis.

The studies conducted herein provided additional detail about the mechanism of inhibitor binding to the SARS-CoV-2 Mpro dimer and highlight that caution needs to be applied when using orthogonal biochemical assays to confirm compound mechanism. Medicinal chemistry relies upon integrating numerous assays to inform compound design. The principle underlying this approach is that the “mechanism” of a compound does not change based on the assay employed. The studies of the nitrile-bearing inhibitors highlight that differences in assay type, protein concentration, and compound incubation time may lead to different conclusions about mechanism. These differences are often dictated by practical constraints. To obtain a co-crystal, for example, changes in buffer pH, PEG concentration etc. may be required, and in turn covalent compound binding may support generation of highly ordered crystals. Our data support that nitriles *can* bind covalently to SARS-CoV-2 but it is unclear the extent to which covalent binding drives potency for the compounds we examined.

Such considerations may be particularly important in assay design for enzymes that display cooperativity in steady state kinetic analysis. For the aldehydes characterized in the study, an unusual mechanism was observed, where one monomer of SARS-CoV-2 formed a stable covalent complex, whereas the other half of the dimer did not, likely a consequence of positive cooperativity in inhibitor binding. To the best of our knowledge, our studies also provide the first evidence for non-covalent binding of an aldehyde to a wild-type cysteine protease. While this may be an observation specific to substrate mimetics, the ability of a compound to initially bind, then to drive the conversion to the more tightly bound complex may be a particularly important variable in the design of potent inhibitors of cooperative multimeric enzymes.

### Limitations of the study

We were unable to conduct the NMR binding studies in the high citrate conditions used for the enzyme inhibition assays and hence not rule out a contribution of the change in buffer conditions for the lack of observed covalent binding for compound 5. In addition, the data for these studies were obtained with a small series of structurally related compounds and could in principle be limited to the compound series examined.

## Resource availability

Materials and protocols are available to qualified researchers with only necessary restrictions and in a timely manner.

### Lead contact

Further information and requests for resources and reagents should be directed to and will be fulfilled by the lead contact, Mark Namchuk (mark_namchuk@hms.harvard.edu).

### Materials availability

Material generated in these studies are detailed within the main article or the supplemental information. All unique/stable reagents generated in this study are available from the lead contact with a completed materials transfer agreement.

### Data and code availability

• All data reported in this article will be shared by the lead contact upon request.
• This article does not report the original code.
• Any additional information required to reanalyze the data reported in this article is available from the lead contact upon request.

## Acknowledgments

Funding to support this work was provided by 10.13039/100006483AbbVie (M.N.N., H.A.), the 10.13039/100020366Massachusetts Consortium on Pathogen Readiness (M.N.N.), the 10.13039/100007429Nancy Lurie Marks Family Foundation (M.N.N.), and the Open eye consortium for academic access to their software (M.N.N.). Use of the IMCA-CAT beamline 17-ID at the Advanced Photon Source was supported by the companies of the 10.13039/100016198Industrial Macromolecular Crystallography Association through a contract with Hauptman-Woodward Medical Research Institute. Use of the Advanced Photon Source was supported by the US Department of Energy, Office of Science, Office of Basic Energy Sciences, under Contract No. DE-AC02-06CH11357. The authors thank David Duda (AbbVie) for assistance in refinement of the X-ray crystal structures and their deposition in the PDF and support from Jennifer Smith and the ICCB-Longwood Screening Facility at Harvard Medical School. The authors thank Robert Schmidt (AbbVie) and Greg Gfesser (AbbVie) for compound synthesis and Liz Noey (AbbVie) for assistance with interpretation of structural data. We thank Hong Liu, Lian Wang, Longfei Li, and Xu Zhou and colleagues (IDSU, WuXi Apptec, Tianjin, China) for the synthesis work. The authors also thank Professors Phil Cole, Tim Mitcheson, and Stephen Blacklow for critical discussion in constructing the manuscript.

## Author contributions

Conceptualization, D.A.D., H.A., and M.N.N.; methodology, K.M.P.D., J.C., S.P., F.P., L.B., A.K., and H.A.; formal analysis, K.M.P.D., J.C., P.S.C., J.G., A.D., D.A.D., H.A., and M.N.N.; investigation, K.M.P.D., J.C., P.S.C., J.G., L.B., S.P., F.P., S.H.S., and H.A.; resources, J.G., A.K., V.S., and G.A.; writing—original draft, K.M.P.D., J.G., A.K., S.P., F.P., L.B., H.A., and M.N.N.; writing—review and editing, R.A.D., D.A.D., H.A., and M.N.N.; visualization, K.M.P.D., J.C., L.B., and M.N.N.; supervision, R.A.D., D.A.D., H.A., and M.N.N.; funding acquisition, M.N.N.

## Declaration of interests

The authors declare the following competing financial interest(s): J.C., S.P., F.P., A.K., V.S., L.B., and D.A.D. are employees of AbbVie, and H.T., G.A., and B.N. are former AbbVie employees and may own AbbVie stock. AbbVie sponsored and funded the study, contributed to the design, and participated in the collection, analysis, and interpretation of data and in writing, reviewing, and approval of the final publication. M.N.N. receives compensation as a scientific advisory board member of OMASS Therapeutics and Sionna Therapeutics. The current affiliation for K.M.P.D. is Department of Structural Biology, St. Jude Children’s Research Hospital, Memphis, TN 38105, USA; for H.T. Vertex Pharmaceuticals, 50 Northern Avenue, Boston, MA 02210, USA; and for B.N. Eli Lilly and Company, Lilly Corporate Center, 893 Delaware St., Indianapolis, IN 46285, USA.

## STAR★Methods

### Key resources table

**Table:** 

REAGENT or RESOURCE	SOURCE	IDENTIFIER
**Antibodies**		

Anti-mouse SARS-CoV-2 Nucleocapsid	Sino Biologicals	40143-R004
Goat anti-mouse IgG (H + L) Highly Cross-Adsorbed Secondary Antibody, Alexa Fluor 488	Thermo Scientific	A-11029; RRID:AB_2534088

**Bacterial and virus strains**		

SARS-CoV-2 Beta (B.1.351, hCoV-19/South Africa/KRISP-K005325/2020)	BEI	Cat# NR-52282
*E. coli* BL21-(DE3)-T1R cells	Sigma-Aldrich	B2935

**Chemicals, peptides, and recombinant proteins**		

Neutravidin	Thermo Scientific	31000
Biotin Tris-NTA	Sigma-Aldrich	75543; CAS: 1070867-85-4
(TAMRA)-dPEG2-S-A-V-L-Q-S-G-dPEG2-K(QSY7)-amide	Abbvie^22^	NA
Ulp1 protease	Abbvie	Ulp1
Benzonase	EMD Chemicals Inc	# 1.01656.0002
Ni Sepharose 6 Fast Flow	Cytiva	#17531802
Ammonium ^15^N chloride	Cambridge Isotope Laboratories	NLM-467-PK
Deuterated water, D_2_O	Cambridge Isotope Laboratories	DLM-4-100
IPTG, Isopropyl β-D-1-thiogalactopyranoside, IPTG, Isopropyl β-D-thiogalactoside	Millipore/Sigma	I6758
M9 media, minimal salts, 5x	Millipore/Sigma	M6030
PEG/Ion HT Screen	Hampton Research	HR2-139
PACT Premier Screen	Molecular Dimensions	MD1-36
^15^N NH_4_Cl_2_	Cambridge Isotope Laboratories Inc.	Cat# NLM-467-10
		CAS Number 39466-62-1
D-GLUCOSE-1,2,3,4,5,6,6-D7	Sigma-Aldrich	Cat#552003-1G
		CAS Number 23403-54-5
D_2_O -DEUTERIUM OXIDE (D, 99.9%)	Cambridge Isotope Laboratories Inc.	Cat# DLM-4-100
		CAS Number 7789-20-0
Benzonase	Millipore Sigma	Cat# 1016560001
		CAS Number: 9025-65-4
Ni Sepharose 6 Fast Flow resin	Cytiva Life Sciences	Cat#17531802

**Critical commercial assays**		

Surface Plasmon Resonance CM5 chip	*Cytiva*	29149603

**Deposited data**		

SARS-CoV-2 Mpro in complex with compound 1	www.rcsb.org	PDB: 9DU3
SARS-CoV-2 Mpro in complex with compound 3	www.rcsb.org	PDB: 9DU4
SARS-CoV-2 Mpro in complex with compound 5	www.rcsb.org	PDB: 9DTZ
SARS-CoV-2 Mpro in complex with compound 7	www.rcsb.org	PDB: 9DU2
Backbone assignment of a C145A Mpro	Robertson et al.^34^	BMRB entry 51455
Surface Plasmon Resonance CM5 chip	*Cytiva*	29149603

**Experimental models: Cell lines**		

A549	ATCC	CCL-185
A549-ACE2	This Study	

**Recombinant DNA**		

pET28a (+) DNA -Novagen	Millipore/Sigma	69864
Severe acute respiratory syndrome coronavirus 2 isolate Wuhan-Hu-1 with amino acids 1-306	GenBank accession number GenBank: NC_045512.2	synthetic
pLEX307-ACE2-puro	Addgene	Cat# 158448
pET28-Mpro (1-306) F305A, Q306A	This Paper	N/A

**Software and algorithms**		

Graphpad Prism 10.0	Graphpad	https://www.graphpad.com/search/?searchquery=download
Biacore T200 Evaluation Software 3.2.1	Cytiva	https://www.cytivalifesciences.com/en/us/shop/protein-analysis/spr-label-free-analysis/spr-software-and-extensions/biacore-t200-software-upgrade-3-1-p-05916
ProSight Native	Proteinaceous Inc.	https://www.proteinaceous.net/prosightnative
Autoproc	Global Phasing Ltd., Cambridge, United Kingdom	N/A
Grade2	Global Phasing Ltd., Cambridge, United Kingdom	N/A
DIMPLE	CCP4 Suite (CCP4.ac.uk)	N/A
Phenix.refine	Phenix (phenix-online.org)	N/A
COOT	CCP4 Suite (CCP4.ac.uk)	N/A
NMRPipe	Delaglio et al.^44^	https://www.ibbr.umd.edu/nmrpipe/
CCPNmr 2.0	Vranken et al.^45^	https://ccpn.ac.uk/software/downloads/
The PyMOL Molecular Graphics System, Version 3.0 Schrödinger, LLC.	Schrodinger (schrodinger.com)	https://pymol.org/
nmrglue.	Helmus^46^	https://www.nmrglue.com/

**Other**		

384-well MultiScreen Filter Plate	EMD Millipore	Cat#MZHVN0W10
ProxiPlate 384-shallow well plus, white	Revvity	Cat#6008280
2970 nm IR laser	JGMA Inc.	http://jgma-inc.com/?page_id=29
Q Exactive HF-X mass spectrometer	Thermo Fisher Scientific	N/A
Bio-Gel P-10 gel	BioRad	Cat#1504144
ProxiPlate 384-shallow well plus, black	Revvity	Cat#6008269
MRC 2 Well Crystallization Plate	Hampton Research	HR3-083
Mosquito Liquid Dispenser	SPT Labtech	N/A

### Method details

#### Protein expression and purification

The DNA construct encoding Mpro, main proteinase of Severe acute respiratory syndrome coronavirus 2 isolate Wuhan-Hu-1 with amino acids 1–306 (GenBank accession number GenBank: NC_045512.2) was synthesized and cloned into a pET28a expression vector using NdeI and XhoI sites. Two types of the expression constructs were generated. The first one, with N-terminal His-tag (8X His) followed with SUMO-protease, was used to prepare protein for functional assays and structural analysis. Ulp1 protease digest as part of the purification protocol of this protein form assured authentic N-and C-termini of the final protein important for the functional testing. The second type of the Mpro expression construct used in SPR was designed with N-terminal SUMO tag and C-terminal His-tag. SUMO tag removal resulted in authentic N-terminus of the purified protein form. Two mutations in this expression construct, F305A and Q306A, were introduced at the C-terminus of Mpro to prevent self-cleavage and removal of the His-tag tag during expression.

Unlabeled Mpro for crystallization and assays was expressed in *E. coli* BL21-(DE3)-T1R cells at varying scales using shake flasks. The bacterial cells from a fresh transformation plate were put into 50 mL of TB media with 50 mg/L of kanamycin and grown overnight at 30°C. Each liter of fresh TB media (plus kanamycin) was inoculated with 10 mL of over-night culture and grown at 37°C to an optical density of 1.5–2.0 with a temperature shift to 18°C for 1 h followed by induction with 0.5 mM IPTG. Expression was carried overnight at 18°C. Cells were harvested by centrifugation at 10,000g for 10 min at 5°C.

The isotope-labeled proteins for NMR were also expressed in *E. coli* BL21-(DE3)-T1R cells at varying scales using flasks. The bacterial cells from a fresh transformation plate were grown overnight at 37°C in LB media made in D_2_O overnight. The cells were pelleted to remove the spent media, and were resuspended in 50 mL of deuterated M9 media supplemented with 1 g/L 15N NH_4_Cl_2_ 2g/L of ^12^C ^2^H Glucose and 50 mg/L of kanamycin. The next day, each liter of deuterated M9 media, was inoculated with 50 mL of over-night culture and grown at 37°C to an optical density of 1 followed by a temperature shift to 18°C and addition of the. The expression was induced with 0.5 mM IPTG and carried overnight at 18°C. Cells were harvested by centrifugation at 10,000g for 10 min at 5°C.

Mpro protein expressed in *E.coli* was purified using the following protocol: cells were thawed, homogenized and lysed in 20 mM Tris, pH 8.0, 150 mM NaCl, 5% Glycerol (w/v), 2 mM MgCl_2_, 5 mM Imidazole, 0.5 mM TCEP, using sonication. Benzonase (EMD Chemicals Inc., cat# 1.01656.0002) was added to the lysate according to the manufacturer’s recommendations. Cell lysate was clarified by centrifugation at 27,000g in a Beckman JLA-16.25 for 40 min at 5°C. Protein was purified using batch mode and 10 mL of Ni Sepharose 6 Fast Flow resin (#17531802, Cytiva) equilibrated in column buffer: 20 mM Tris, pH 8.0, 150 mM NaCl, 5% Glycerol (w/v), 0.5 mM TCEP, 5 mM imidazole. Protein was eluted from the column using column buffer with 250 mM imidazole after step wash with 20 mM imidazole. N-terminal His-SUMO or SUMO tag were removed with Ulp1 (in house made) digest performed overnight at 4°C. Digested protein was harvested in flow-through of Ni Sepharose 6 Fast Flow column using the same buffers as first purification chromatography step. Protein was further purified using SEC Superdex 200 chromatography with 20 mM HEPES pH 7.5, 100 mM NaCl, 0.5mM TCEP or PBS, pH 7.4 with 0.5 mM TCEP. The molecular weight of all purified proteins was confirmed by mass spectroscopy.

#### K_i_ determination and onset of inhibition by kinetic assay

SARS-CoV-2 Mpro biochemical assays are performed in 8 μL in 384-well Proxi-Plus plate (PerkinElmer, Waltham, MA) at ambient temperature in the optimized assay buffer (50 mM Hepes, pH 7.1, 800 mM Sodium Citrate, 1 mM EDTA, 1 mM TCEP, 0.005% BSA) with 1 nM of recombinant Mpro and 5 μM Substrate. After 15 min of preincubation of inhibitor with Mpro in assay buffer, reactions are initiated by the addition of an FRET-compatible peptide substrate (TAMRA)-dPEG2-S-A-V-L-Q-S-G-dPEG2-K(QSY7)-amide. Fluorescence is measured at 20 min using a 531/579 excitation/emission filters on EnVision (PerkinElmer, Waltham, MA). Data are normalized to DMSO minus no enzyme controls. IC_50_ and K_i_ of inhibitors were determined from a 34-point compound titration and analyzed using Graphpad Prism 10.0 and either fit to the Morrison tight binding equation or a 4 parameter IC_50_ fit. For the Morrison equation, data were fit to the equation Y=Vo∗(1-((((Et+X+Q)-(((Et+X+Q)ˆ2)-4∗Et∗X)ˆ0.5))/(2∗Et))). E_total_ was fixed at 1 nM, Km at 2.5 μM. IC_50_ was fit to the equation Y=Bottom +(Top-Bottom)/1 + 10ˆ((Log IC_50_-X)∗Hillslope. No constraints were applied to the fit. The timecourse studies (Figure S2) were conducted using a similar protocol without the 15 min preincubation period with substrate and inhibitor addition occurring simultaneously. FRET signal was measured for 1200 s at 5 s intervals.

#### SPR studies

Compounds were assayed *via* Surface Plasmon Resonance (SPR) using Biacore T200 instrument. His tag (8-His at C-terminus) SARS-CoV-2 3CLpro (1-306) F305A, Q306A protein was immobilized on neutravidin surface saturated with biotinylated Tri-NTA-Ni^+2^ on different flow cells and compounds were profiled in single-cycle kinetics mode. The compounds were diluted in the running buffer (10 mM Hepes, pH 7.5, 150 mM NaCl, 0.005% Tween-20, 1 mM TCEP; Final running DMSO concentration was 3%) and injected in a series of increasing concentrations at flow rate of 80 μL/min for contact time of 70 s and dissociation was monitored for up to 3000 s. After each compound dose response, protein surface was regenerated using 60 s of Glycine (pH 2.0) and 60 s of 250 mM EDTA (pH 8.0) solutions and fresh protein was captured on surface before each compound kinetics measurement. Sensorgrams were processed and analyzed using Biacore T200. The binding curves were fit to determine the equilibrium dissociation constant (K_D_) and kinetic constants (k_on_ and k_off_).

#### Covalent bond formation kinetics by MALDESI

10 μM (final) Mpro recombinant protein were added into a 384 well dispensed with 10 μM (final) compounds in either citrate buffer (50 mM Hepes pH 7.1, 650 mM Na3C6H5O7, 1 mM EDTA, 0.5 mM TCEP) or SPR buffer (10 mM Hepes pH 7.5, 150 mM NaCl, 0.005% Tween, 3% DMSO, 0.5 mM TCEP) at different time points so that the incubation time between Mpro and compounds were at 0.5, 3, 9, 27 and 81 min before adding 200 μM (final) PF-00835231 for quenching and buffer exchange.

High throughput buffer exchange was performed using custom-made SEC plates. To make the SEC plates, Bio-Gel P-10 gel (BioRad) was swollen in 50 mM ammonium acetate then dispensed into a 384-well MultiScreen filter plate (EMD Millipore). The SEC plates were spun at 800 g for 2 min to remove storage buffer before loading samples. Buffer exchange was performed twice for each sample by stacking two SEC plates on top of a 384-well collection plate (Proxiplate, Perkin Elmer) and spinning at 800g for 2 min. IR-MALDESI analysis was performed on the collection plate.

A home-built IR-MALDESI coupled to a Q Exactive HF-X mass spectrometer, as previously published,^33^ (Thermo Fisher Scientific, Bremen) was used to acquire intact protein MS data. Briefly, a 2970 nm IR laser (JGMA Inc.) was used to desorb samples from a microtiter plate, the desorbed samples were then ionized by electrospray (ESI). ESI solvent was 80:20 methanol:water v/v with 0.1% formic acid. MS data was acquired in positive ion mode with an *m/z* range of 1000 to 2500. Injection time was fixed at 100 ms with automatic gain control turned off, and mass resolution was set at 7,500 (FWHM at *m/z* = 200). The acquired raw mass spectra were then deconvoluted for calculating % adduct. Protein deconvolution was performed using ProSight Native (Proteinaceous Inc.) following a reported workflow.^33^^,^^47^ Minimum SNR for deconvolution was 3. Percent adduct formation was calculated using the following equation: %Adduct= (I_bound_/I_bound_+I_unbound_)X100. In this equation, I_bound_ and I_unbound_ are the deconvoluted intensities of bound and unbound protein, respectively. Each time point is the average of 5 replicate assessments. Sample recovery was in some cases too low to accurately calculate the %adduct. These datapoints were excluded from the analysis. Data presented in the graph are generally averages of 5 determinations to help overcome 8.3% missing data points due to loss of protein during high throughput buffer exchange using custom-made SEC plates.

The rate of covalent bond formation (kobs) was estimated by fitting the amount of covalent complex over time to the slow tight binding equation Y=Vs∗X+((Vi-Vs)/kobs)∗(1-exp(-kobs∗X)). Data were fit with no constraints using Graphpad Prism 10.0.

#### X-Ray structure determination

Mpro protein sample (SARS-CoV-2 3CL protease residues 1-306) was concentrated to 9.35 mg/mL in buffer composed of 20 mM Tris pH 8.0, 150 mM NaCl, and 0.5 mM TCEP. Compounds were dissolved in DMSO to 200 mM and added to the protein at a molar ratio of 5:1 (compound:protein). The complexes were screened using the sitting-drop vapor diffusion technique in 96-well MCR 2 Well plates (Hampton Research, Aliso Viejo, CA, USA) with 0.1 μL of protein and 0.1 μL of screening solution over wells containing 40 μL screening solution using a Mosquito liquid dispenser (TTP Labtech, Cambridge, MA, USA). Several commercially available crystallization screens were used and specific conditions for each complex are described below. All plates were stored at 23°C.

##### Compound 1 structure (PDB: 9DU3)

Crystals of Mpro in complex with compound 1 appeared within 1 to 3 days. The best condition was from the PEG/Ion HT screen (Hampton Research) well D2, consisting of 20 % PEG 3350 and 0.2 M ammonium tartrate dibasic. The crystals were harvested using 25% glycerol as a cryo-protectant and were flash frozen in liquid nitrogen. Diffraction data were collected at the IMCA-CAT beamline 17-ID at the Advanced Photon Source (Argonne National Laboratory, Lemont, IL, USA).

##### Compound 3 structure (PDB: 9DU4)

Crystals of Mpro in complex with compound 3 appeared within 8 days. The best condition was from the PEG/Ion HT screen (Hampton Research, Viejo, CA, USA) well B3, consisting of 20% PEG 3350 and 0.2 M lithium nitrate. The crystals were harvested using 25% glycerol as a cryo-protectant and were flash frozen in liquid nitrogen. Diffraction data were collected at Beamline I04-1 at Diamond Light Source (Didcot, United Kingdom).

##### Compound 5 structure (PDB: 9DTZ)

Crystals of Mpro in complex with compound 5 appeared within 1 to 3 days. The best condition was from the PACT premier screen (Molecular Dimensions, Holland, OH, USA) well B7, consisting of 20% PEG 6000, 0.1 M MES pH 6.0 and 0.2 M sodium chloride. The crystals were harvested using 25% glycerol as a cryo-protectant and were flash frozen in liquid nitrogen. Diffraction data were collected at the IMCA-CAT beamline 17-ID at the Advanced Photon Source (Argonne National Laboratory, Lemont, IL, USA).

##### Compound 7 structure (PDB: 9DU2)

Crystals of Mpro in complex with compound 7 appeared within 1 day. The best condition was from the PACT premier screen (Molecular Dimensions) well F7, consisting of 20% PEG 3350, 0.1 M Bis-tris propane pH 6.5, and 0.2 M sodium acetate. The crystals were harvested using 25% glycerol as a cryo-protectant and were flash frozen in liquid nitrogen. Diffraction data were collected at the IMCA-CAT beamline 17-ID at the Advanced Photon Source (Argonne National Laboratory, Lemont, IL, USA).

##### Data processing and refinement

Diffraction data reduction and scaling was performed with Autoproc (Global Phasing Ltd., Cambridge, United Kingdom), and small molecule restraints for refinement were generated with Grade2 (Global Phasing). Molecular replacement was initially performed with PDB ID 6XHM as the input model, then with subsequently refined models using Dimple as part of the CCP4 suite. Structure refinement was performed with phenix.refine (Phenix reference) and fitting was performed in Coot (CCP4). Structure figures were generated in PyMOL (Schrodinger, New York, NY).

#### NMR binding studies

^1^H-^15^N HSQC -TRSOY spectra were recorded at 298 K on an 800 MHz Bruker spectrometer equipped with an AVANCE III console and a cryogenically cooled probe. Fourty μM of deuterated ^15^N labeled Mpro was incubated with 0, 20 or 40 μM compound 1 or compound 5, and HSQC -TROSY spectra were recorded. The acquired spectra were processed using NMRPipe^44^ and were analyzed using CCPNmr 2.0.^45^ The chemical shifts were transferred from BMRB entry 51455.^34^ Assignments from very crowded regions of the spectra were removed to avoid ambiguous assignments. The chemical shift perturbations (CSPs) on both ^1^H and ^15^N dimensions were used to calculate the weighted CSP.^35^ The difference between the CSP values (Δ δHN) for each residue was calculated and these values were then plotted onto the PDB structure using pymol to map the regions that showed significant differences in chemical shifts upon binding to compound 1 in comparison to compound 5.

The ^13^C labeled ligand detected experiments were carried out on the same magnet as above using a 1D ^13^C experiment with proton decoupling with carbon centered at 150 ppm 300 μM of compound 1or compound 5 was mixed with 100 μM of unlabeled Mpro and ^13^C 1 D experiments were run at an interval of 28 min each. The 1D spectra were processed in NMRPipe and the curves were plotted using nmrglue.^46^

#### Conformation of the absence of epimerization for Compound 1

To confirm that compound 1 did not undergo epimerization at P1 under the buffer conditions used for the MALDESI and NMR studies, we monitored the NMR signal of the hydrogen (denoted as αH in Figure S6) after transferring the compound to D_2_O containing SPR buffer where epimerization would lead to a loss of signal for the αH resonance over time.

The αH exhibits a doublet of triplets due to coupling with adjacent hydrogens. To assign this hydrogen unambiguously, we performed 2D COSY, 2D TOCSY, and 2D JRES experiments on compound 1, as well as a long-range ^1^H-^13^C HMBC experiment on ^13^C-aldehyde-labeled compound 1.

The 2D J-resolved (JRES) NMR spectrum provides ^1^H chemical shifts in the direct dimension and ^1^H scalar couplings in the indirect dimension. After processing, which includes tilting and symmetrizing the 2D resonances, the 1D projection in the direct dimension yields a ^1^H-decoupled 1D spectrum. The combination of long-range HMBC, 2D COSY, and TOCSY confirmed the assignment of αH to 3.86 ppm. The JRES NMR coupling pattern further strengthened our confidence in this assignment, despite the proximity of the αH peak to an intense buffer peak in the 1D spectrum (Figures S6A and S6B).

The experiments on compound 1 were acquired on NMR spectrometer operating at 600 MHz ^1^H Larmor frequency and equipped with cryogenically cooled probe. 1D ^1^H NMR was acquired with 32768 points in the direct dimension (16 ppm spectral width and 1.7 s acquisition time) with 256 number of scans and 2 s recycle delay. 2D COSY NMR spectrum was recorded with 4096 and 1024 points (real + imaginary) in the direct and indirect dimension, respectively. The spectrum was acquired with 24 scans, 2 s recycle delay and 15 ppm spectral width (227 ms and 57 ms acquisition time in direct and indirect dimension respectively) in both the dimensions. 2D JRES NMR spectrum was acquired with 8192 and 80 points in the direct and indirect dimension with 64 scans and 1 s recycle delay. Spectral width was 16.6 ppm and 0.08 ppm with acquisition time 410 ms and 800 ms in the direct and indirect dimension, respectively. 2D TOCSY NMR spectrum was acquired with 2048 and 256 points (real + imaginary), 128 scans and 1 s recycle delay. The TOCSY mixing time was set to 80 ms. Spectral width was 10 ppm in both the dimensions with acquisition time of 170 and 21 ms in the direct and indirect dimension, respectively. In all the experiments the carrier frequency was set to 4.7 ppm.

##### Test of epimerization

10 mL of SPR buffer was lyophilized, resuspended in D_2_O, and used in the epimerization studies. Although the resonance of the αH hydrogen overlapped with the shoulders of the intense buffer resonance, we were able to clearly identify and track the αH resonance over time. Compound 1 (500 μM) was diluted into either H_2_O or D_2_O buffer from a 10 mM DMSO stock. The first measurement was taken 10 min after sample preparation, and the resonance was monitored for 10 h at 16-min intervals. The αH resonance in the D_2_O sample showed no change in intensity over the entire 10-h observation period. An overlay of the spectra from the first three hours, corresponding to the biochemical experiments, is shown in Figure S6A.

We also conducted two-dimensional ^1^H-^13^C HMBC experiments optimized for long-range couplings to monitor potential epimerization over a 12-h period. No epimerization was observed during this time (Figure S6E).

#### Anti-viral potency determination

Compounds were assessed for anti-viral activity using the method of Patten et al.^30^ A 10-point compound titration was conducted started at 50 μM diluted in 3.16-fold increments. Activity was scored on a per well basis as the ratio of cells positive for SARS-CoV-2 N-Protein versus total cell count determined by nuclear staining. Assays were conducted in triplicate. The cell lines were purchased from ATCC and certified to be A549 cells and free of mycoplasma at the time of purchase.

#### Compound synthesis and analytical data

All compounds with the exception of compound 3 were synthesized at WuXi Apptec (Tianjin). Compound 3 was synthesized at Abbvie.

#### Compound synthetic schemes and analytical data

##### Abbreviations

CMPI chloro-1-methylpyridinium iodide
Cpa (S)-cyclopropylalanine
DBU 2,3,4,6,7,8,9,10-octahydropyrimido [1,2-a]azepine
DCM dichloromethane
DIEAN,N-diisopropylethylamine
EDCI1-(3-dimethylaminopropyl)-3-ethylcarbodiimide hydrochloride
HATUN N,N′,N′-tetramethyl-O-(7-azabenzotriazol-1-yl) uronium hexafluorophosphate
HOAt1-hydroxy-7-azabenzotriazole
NMM N-methylmorpholine
TFAA trifluoracetic anhydride

#### Preparation of (*S*)-2-(((benzyloxy)carbonyl)amino)-3-cyclopropylpropanoic acid (Cbz-CpaOH)

To a mixture of HCpaOH (15 g, 116mmol) and NaOH (15.6 g, 390 mmol) in H_2_O (390 mL) was added benzyl chloroformate (23.8 g, 139.4 mmol, 19.8mL) dropwise at 0°C. The mixture was stirred at 20°C for 30 h. The reaction mixture was extracted with DCM (100 mL) and the organic phase was discarded. The aqueous phase was acidified with concentration HCl to adjust the pH ∼1 and then extracted with DCM (3 × 200 mL). The combined organic phases were washed with water (200 mL), dried over anhydrous Na_2_SO_4_, filtered and concentrated under reduced pressure to give Cbz-CpaOH (20.3 g, crude) as a pale yellow oil.

^**1**^**H NMR**: (400 MHz, CDCl_3_)

δ 8.22 (s, 1 H), 7.32–7.38 (m, 5 H), 5.44 (d, J = 8.0 Hz, 1 H), 5.09–5.20 (m, 2 H), 4.45–4.57 (m, 1 H), 1.76 (t, J = 6.4 Hz, 2 H), 0.69–0.82 (m, 1 H), 0.41–0.58 (m, 2 H), 0.13 (d, J = 2.4 Hz, 2H).

#### Preparation of (*S*)-2-((*tert*-butoxycarbonyl)amino)-3-cyclopropylpropanoic acid (Boc-CpaOH)

To a mixture of HCpaOH (10 g, 77.4 mmol) and K_2_CO_3_ (36.4 g, 263 mmol) in THF (120 mL) and H_2_O (120 mL) was added (Boc)_2_O (21.97 g, 101 mmol) at 0°C. The resulting mixture was stirred at 20°C for 12 h. THF was removed under reduced pressure. The aqueous phase was acidified to pH ∼3 with 1 N citric acid aqueous. The resulting mixture was extracted with DCM (3 × 100 mL). The combined organic phases were washed with water (100 mL), dried over anhydrous Na_2_SO_4_, filtered and concentrated. The crude was purified by column chromatography (SiO_2_, petroleum ether/EtOAc = 10/1 to 3/1) to give Boc-CpaOH (17.5 g, 98.6% yield) as a pale yellow oil.

^**1**^**H NMR** 400 MHz, CDCl_3_

δ 5.15 (d, J = 6.8 Hz, 1 H), 4.41 (d, J = 6.0 Hz, 1 H), 1.73 (t, J = 6.0 Hz, 2 H), 1.47 (s, 9 H), 0.77 (s, 1 H), 0.51 (d, J = 4.0, 2 H), 0.14 (d, J = 4.0 Hz, 2 H).

#### Scheme 1: Preparation of Compound 6

**Figure:**
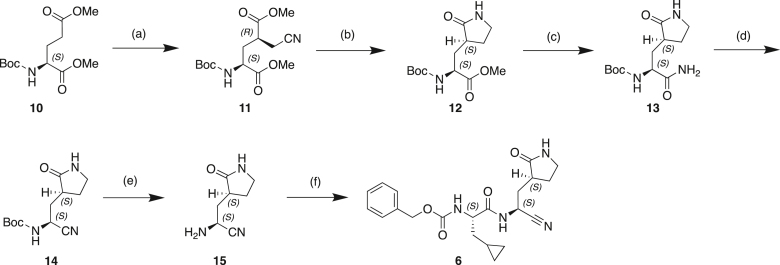

(a) BrCH_2_CN, LHMDS, THF, −70°C; (b) CoCl_2._6H_2_O, NaBH_4_, MeOH, 0°C–25°C; (c) Mg(OEt)_2_, NH_3_, MeOH, toluene, 25°C; (d) TFAA, Et_3_N, THF, 20°C; (e) TFA, DCM, 20°C; (f) Cbz-CpaOH, CMPI, NMM, DCM, 20°C.

#### Preparation of dimethyl (2*S*,4*R*)-2-((*tert*-butoxycarbonyl)amino)-4-(cyanomethyl)pentanedioate (11)

To a solution of Boc-(S)-glutamic acid dimethyl ester (**10**) (70 g, 254 mmol) in THF (700 mL) was added a solution of LiHMDS (1 M, 549 mL) dropwise at −70°C under an argon atmosphere. The resulting mixture was stirred at −70°C for 1 h. At the same time, 2-bromoacetonitrile (50 g) was stirred with basic aluminum oxide (9 g) for 2 h and then filtered. The freshly filtered 2-bromoacetonitrile (32.63 g, 272.1 mmol, 18.1 mL) was added to the above mixture while maintaining the temperature below −70°C. The reaction mixture was stirred at −70°C for another 2 h. The reaction was quenched with cold MeOH (35 mL) and stirred for 30 min. The resulting methoxide was then quenched with a cold AcOH in THF (31.5 mL AcOH/210 mL THF) in one portion. After stirring for 30 min, the reaction mixture was allowed to warm up to 0°C–5°C and then poured into a brine solution. The resulting mixture was extracted with EtOAc (3 × 800 mL). The combined reaction mixture was washed with brine (800 mL), dried over Na_2_SO_4_, filtered and concentrated under reduced pressure to give crude product. The crude product was purified by column chromatography (SiO_2_, petroleum ether/EtOAc = 1/0 to 0/1) to give **11** (40 g, 44% yield) as a yellow oil.

^**1**^**H NMR:** 400MHz CDCl_3_

δ 5.12 (br d, J = 6.84 Hz, 1 H) 4.39 (br d, J = 4.19 Hz, 1 H) 3.77 (d, J = 4.19 Hz, 6 H) 2.71–2.92 (m, 3 H) 2.09–2.25 (m, 2 H) 1.45 (s, 9 H).

#### Preparation of methyl (*S*)-2-((*tert*-butoxycarbonyl)amino)-3-((*S*)-2-oxopyrrolidin-3-yl)propanoate (12)

To a mixture of **11** (12.5 g, 39.8 mmol) in MeOH (250 mL) was added CoCl_2_.6H_2_O (5.7g, 23.9 mmol) in one portion at 0°C. After addition, NaBH_4_ (9. g, 238.6 mmol) was added in portions under N_2_ atmosphere at 0°C. The mixture was warmed to 25°C and stirred at 25°C for 12 h. The reaction mixture was poured into saturated ammonium chloride solution (100 mL). Five additional vials were set up as described above and all six reaction mixtures were combined. The combined resulting mixture were filtered with celite and the filtrate was distilled off under reduced pressure, extracted with DCM (3 × 750 mL). The organic phase was combined, washed with water (1 L), dried over Na_2_SO_4_, filtered and concentrated under reduced pressure to give a residue. The residue was purified by column chromatography (SiO_2_, petroleum ether/EtOAc = 2/1 to 0/1) to give **12** (28 g, 49% yield) as a white solid.

^**1**^**H NMR:** 400MHz CDCl_3_

δ 6.29 (br s, 1 H) 5.54 (br d, J = 8.16 Hz, 1 H) 4.25–4.36 (m, 1 H) 3.74 (s, 3 H) 3.32–3.37 (m, 2 H) 2.43–2.52 (m, 2 H) 2.09–2.17 (m, 1 H) 1.79–1.86 (m, 2 H) 1.44 (s, 9 H).

#### Preparation of *tert*-butyl ((*S*)-1-amino-1-oxo-3-((*S*)-2-oxopyrrolidin-3-yl)propan-2-yl)carbamate (13)

To a mixture of **12** (3 g, 10.5 mmol) and diethoxymagnesium (2.64 g, 23.1 mmol) in toluene (48 mL) was added a solution of NH_3_/MeOH (7 M, 12 mL). The mixture was stirred at 20°C for 20 h in a sealed tube under N_2_ atmosphere. The reaction mixture was diluted with EtOAc (50 mL), filtered with celite. The filtrate was concentrated in vacuo. The crude was purified by column chromatography (SiO_2_, EtOAc: MeOH = 10:1 to 3:1) to give **13** (2.5 g, 85% yield) as a white solid.

^**1**^**H NMR:** 400MHz CDCl_3_

δ 7.00–7.23 (m, 1 H) 6.90 (br d, J = 13.67 Hz, 1 H) 6.19–6.40 (m, 1 H) 6.08 (br d, J = 5.51 Hz, 1 H) 4.24–4.45 (m, 1 H) 3.25–3.41 (m, 2 H) 2.51 (dt, J = 15.66, 7.83 Hz, 1 H) 2.29–2.40 (m, 1 H) 2.05–2.12 (m, 1 H) 1.74–1.90 (m, 2 H) 1.41–1.43 (m, 9 H).

#### Preparation of *tert*-butyl ((*S*)-1-cyano-2-((*S*)-2-oxopyrrolidin-3-yl)ethyl)carbamate (14)

To a mixture of **13** (1 g, 3.7 mmol) and Et_3_N (746 mg, 7.4 mmol, 1.03 mL) in THF (60 mL) was added TFAA (1.55 g, 7.4 mmol, 1.03 mL) dropwise at 0°C under N_2_ atmosphere. The mixture was warmed to 20°C and stirred for 1 h. A solution of NaHCO_3_ (2 g) in water (10 mL) were added to the above reaction mixture. The resulting mixture was concentrated to remove THF. The aqueous phase was extracted with EtOAc (3 × 20 mL). The combined organic phase was washed with aqueous NaHCO_3_ solution (5%, 20 mL), brine (20 mL), dried over anhydrous Na_2_SO_4_, filtered and concentrated under reduced pressure to give a residue. The residue was purified by column chromatography (SiO_2_, petroleum ether/EtOAc = 1/1 to 0/1) to give **14** (730 mg, 73.7% yield) as a white solid.

^**1**^**H NMR:** 400MHz CDCl_3_

δ 6.07 (br s, 1 H) 5.86 (br d, J = 7.06 Hz, 1 H) 4.63–4.80 (m, 1 H) 3.33–3.46 (m, 2 H) 2.40–2.56 (m, 2 H) 2.22–2.36 (m, 1 H) 1.81–1.99 (m, 2 H) 1.44–1.50 (m, 9 H).

**LCMS (ESI+)**: m/z 197.9 (M-55)^+^

#### Preparation of (*S*)-2-amino-3-((*S*)-2-oxopyrrolidin-3-yl)propanenitrile (15)

To a solution of **14** (200 mg, 789.6 μmol) in DCM (3 mL) was added TFA (2 mL) dropwise at 0°C. The reaction solution was stirred at 20°C for 1 h and concentrated in vacuo. The residual material was added dropwise to NaHCO_3_ (1 g) in THF (10 mL) and stirred at 20°C for 10 h. The resulting mixture was filtered, and the filtrate concentrated in vacuo to afford **15** (120 mg) as a yellow oil.

^**1**^**H NMR:** 400MHz DMSO-d_6_

δ 3.70–3.93 (m, 1 H) 3.10–3.24 (m, 2 H) 2.31–2.42 (m, 1 H) 2.19–2.28 (m, 1 H) 1.87–2.00 (m, 1 H) 1.51–1.71 (m, 2 H).

**LCMS (ESI+)**: m/z 154.0 (M+1)^+^.

#### Preparation of benzyl ((*S*)-1-(((*S*)-1-cyano-2-((*S*)-2-oxopyrrolidin-3-yl)ethyl)amino)-3-cyclopropyl-1-oxopropan-2-yl)carbamate (6)

A mixture of **15** (99.46 mg, 372.2 μmol) and NMM (161.35 mg, 1.6 mmol, 175.4 μL) in DCM (2 mL) was stirred at 0°C for 0.5 h. Cbz-CpaOH (70 mg, 265.9 μmol) and CMPI (170 mg, 664.7 μmol) were added. The resulting mixture was stirred at 0°C for 3 h and was concentrated in vacuo to give a residue which was dissolved in DMF (0.5 mL) and filtered. The solution was purified by prep-HPLC (column: Phenomenex Gemini-NX C18 75 × 30 mm × 3 μm; mobile phase: [water (10 mM NH_4_HCO_3_)- MeCN]; B%: 25%–55%, 12 min) to afford **6** (5 mg, 41.8% yield) as a white gum.

^**1**^**H NMR:** 400MHz DMSO-d_6_

δ 8.77–9.01 (m, 1 H) 7.65–7.80 (m, 1 H) 7.55 (br t, J = 8.71 Hz, 1 H) 7.35 (br d, J = 5.29 Hz, 5 H) 4.76–5.17 (m, 3 H) 4.01 (br d, J = 6.62 Hz, 1 H) 2.93–3.18 (m, 2 H) 2.03–2.41 (m, 3 H) 1.52–1.84 (m, 3 H) 1.21–1.43 (m, 1 H) 0.72 (br s, 1 H) 0.38 (br s, 2 H) 0.03–0.20 (m, 2 H).

**LCMS (ESI+)**: m/z 399.2 (M + H)^+^.

#### Scheme 2: Preparation of Compound 5

**Figure:**
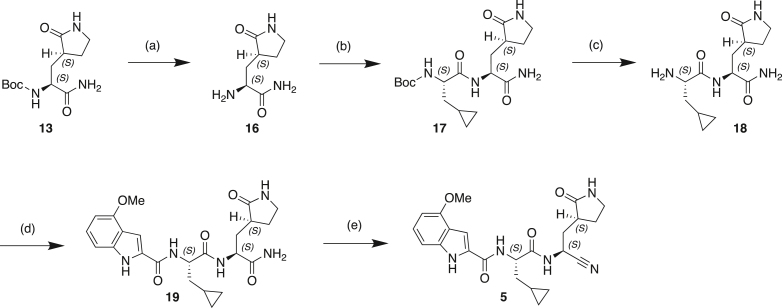

(a) TFA, DCM, 0°C–20°C; (b) Boc-CpaOH, CMPI, NMM, DCM, 20°C; (c) TFA, DCM; (d) 4-methoxy-1H-indole-2-carboxylic acid, CMPI, NMM, DCM, 20°C; (e) TFAA, Et_3_N, THF, 20°C.

#### Preparation of (*S*)-2-amino-3-((*S*)-2-oxopyrrolidin-3-yl)propenamide (16)

To a solution of **13** (1.1 g, 4.1 mmol) in DCM (18 mL) was added TFA (4.6 g, 40.5 mmol, 3.00 mL) dropwise at 0°C. The reaction was stirred at 20°C for 1 h. The reaction solution was concentrated in vacuo to give **16** (1.4 g, crude, TFA salt) as a yellow oil which was used to next step without further work-up or purification.

^**1**^**H NMR:**400MHz DMSO-d_6_

δ 8.24 (br s, 2 H) 7.98 (s, 1 H) 7.84–7.93 (m, 1 H) 7.59 (br s, 1 H) 3.72–3.97 (m, 1 H) 3.20 (br s, 2 H) 2.42–2.49 (m, 1 H) 2.21–2.34 (m, 1 H) 1.92–2.05 (m, 1 H) 1.66–1.83 (m, 2 H).

**LCMS (ESI+)**: m/z 172.1 (M + H)^+^.

#### Preparation of *tert*-butyl ((*S*)-1-(((*S*)-1-amino-1-oxo-3-((*S*)-2-oxopyrrolidin-3-yl)propan-2-yl)amino)-3-cyclopropyl-1-oxopropan-2-yl)carbamate (17)

A mixture of **16** (1.3 g, 4.7 mmol TFA salt) and NMM (4 g, 39.3 mmol, 4.3 mL) in DCM (30 mL) was stirred at 0°C for 0.5 h. Boc-CpaOH (0.9 g, 3.9 mmol) and CMPI (2.5 g, 9.8 mmol) were added to the above mixture in order. The resulting mixture was stirred at 0°C for 10 h. The reaction solvent was removed by evaporation and the residue was dissolved into DMF (5 mL). The resulting solution was filtered and purified by prep-HPLC (column: Phenomenex Gemini-NX C18 75 × 30 mm × 3 μm; mobile phase: [water (10 mM NH_4_HCO_3_) - MeCN]; B%: 5%–35%, 12 min) to give **17** (500 mg, 33.3% yield) as a white solid.

^**1**^**H NMR:** 400MHz DMSO-d_6_

δ 7.86 (br d, J = 8.60 Hz, 1 H) 7.52–7.67 (m, 1 H) 7.24–7.40 (m, 1 H) 6.92–7.15 (m, 2 H) 4.19–4.34 (m, 1 H) 3.87–3.98 (m, 1 H) 3.01–3.19 (m, 2 H) 2.10–2.30 (m, 2 H) 1.92–2.03 (m, 1 H) 1.59–1.70 (m, 1 H) 1.43–1.56 (m, 2 H) 1.37 (s, 10 H) 0.72 (br s, 1 H) 0.27–0.45 (m, 2 H) −0.05 - 0.14 (m, 2 H).

**LCMS (ESI+)**: m/z 383.2 (M + H)^+^.

#### Preparation of (*S*)-2-amino-*N*-((*S*)-1-amino-1-oxo-3-((*S*)-2-oxopyrrolidin-3-yl)propan-2-yl)-3-cyclopropylpropanamide (18)

To a solution of **17** (200 mg, 522.3 μmol) in DCM (5 mL) was added TFA (1 mL) dropwise at 0°C. The reaction solution was stirred at 0°C for 2 h. The mixture was concentrated in vacuo to give **18** (207 mg, crude, TFA salt) as a yellow oil which was used in the next step without work-up or further purification.

^**1**^**H NMR:** 400MHz DMSO-d_6_

δ 8.56 (br d, J = 8.44 Hz, 1 H) 7.64–7.72 (m, 1 H) 7.51–7.61 (m, 1 H) 7.07–7.18 (m, 1 H) 4.28–4.47 (m, 1 H) 3.89 (br d, J = 6.11 Hz, 1 H) 3.06–3.23 (m, 2 H) 2.13–2.34 (m, 2 H) 1.93–2.06 (m, 1 H) 1.45–1.79 (m, 4 H) 0.73 (br s, 1 H) 0.33–0.49 (m, 2 H) 0.11 (br s, 2 H).

**LCMS (ESI+)**: m/z 283.3 (M + H)^+^.

#### Preparation of *N*-((*S*)-1-(((*S*)-1-amino-1-oxo-3-((*S*)-2-oxopyrrolidin-3-yl)propan-2-yl)amino)-3-cyclopropyl-1-oxopropan-2-yl)-4-methoxy-1*H*-indole-2-carboxamide (19)

To a solution of **18** (199 mg, 502.1 μmol, TFA salt) in DCM (5 mL) was added NMM (254 mg, 2.5mmol, 276 μL) dropwise at 0°C and the resulting mixture was stirred at 0°C for 30 min 4-Methoxy-1H-indole-2-carboxylic acid (80 mg, 418.5 μmol) and CMPI (267 mg, 1.1 mmol) were added to the above mixture and stirred at 20°C for 2 h. The reaction solvent was removed by evaporation and the residue was dissolved in DMF (2 mL). The crude was purified by prep-HPLC (column: Phenomenex Gemini-NX C18 75 × 30 mm × 3 μm; mobile phase: [water (10 mM NH_4_HCO_3_) - MeCN]; B%: 20%–40%, 8 min) to give **19** (70 mg, 36.7% yield) as a yellow solid.

^**1**^**H NMR:** 400MHz DMSO-d_6_

δ 11.58 (s, 1 H) 8.43 (br d, J = 7.72 Hz, 1 H) 8.03 (d, J = 8.60 Hz, 1 H) 7.52–7.63 (m, 1 H) 7.26–7.37 (m, 2 H) 6.96–7.14 (m, 3 H) 6.51 (d, J = 7.72 Hz, 1 H) 4.43–4.58 (m, 1 H) 4.22–4.35 (m, 1 H) 3.89 (s, 3 H) 3.01–3.17 (m, 2 H) 2.22–2.32 (m, 1 H) 2.09–2.17 (m, 1 H) 1.95–2.05 (m, 1 H) 1.44–1.82 (m, 4 H) 0.75–0.87 (m, 1 H) 0.31–0.45 (m, 2 H) 0.06–0.24 (m, 2 H).

**LCMS (ESI+)**: m/z 456.3 (M + H)^+^.

#### Preparation of *N*-((*S*)-1-(((*S*)-1-cyano-2-((*S*)-2-oxopyrrolidin-3-yl)ethyl)amino)-3-cyclopropyl-1-oxopropan-2-yl)-4-methoxy-1*H*-indole-2-carboxamide (5)

To a mixture of **19** (40 mg, 88 μmol) and Et_3_N (267 mg, 2.63 mmol, 367 μL) in THF (2.4 mL) was added TFAA (92 mg, 439.1 μmol, 61 μL) dropwise at 0°C under N_2_ atmosphere. The reaction mixture was stirred at 20°C for 10 h. The reaction solvent was removed by evaporation and the residue was dissolved in DMF (2 mL). The solution was purified by prep-HPLC (column: Phenomenex Gemini-NX C18 75 × 30 mm × 3 μm; mobile phase: [water (10 mM NH_4_HCO_3_) - MeCN]; B%: 20%–50%, 12 min) to give **5** (7.2 mg, 19% yield) as a white solid.

^**1**^**H NMR:** 400MHz DMSO-d_6_

δ 11.57 (s, 1 H) 8.86–9.02 (m, 1 H) 8.50 (d, J = 7.50 Hz, 1 H) 7.66–7.78 (m, 1 H) 7.36 (d, J = 1.54 Hz, 1 H) 7.06–7.13 (m, 1 H) 6.98–7.03 (m, 1 H) 6.50 (d, J = 7.50 Hz, 1 H) 4.87–5.03 (m, 1 H) 4.38–4.54 (m, 1 H) 3.89 (s, 3 H) 3.05–3.20 (m, 2 H) 2.35–2.42 (m, 1 H) 2.01–2.29 (m, 2 H) 1.63–1.90 (m, 3 H) 1.39–1.58 (m, 1 H) 0.80 (br d, J = 5.51 Hz, 1 H) 0.33–0.51 (m, 2 H) 0.03–0.26 (m, 2 H).

**LCMS (ESI+)**: m/z 438.2 (M + H)^+^.

#### Scheme 3: Preparation of Compound 2

**Figure:**
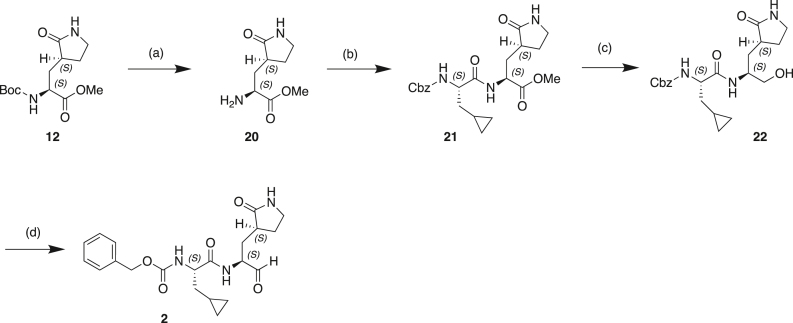

(a) TFA, DCM; (b) Cbz-CpaOH, CMPI, NMM, DCM, 0°C–25°C; (c) LiBH_4_, MeOH; (d) DMP, DCM.

#### Preparation of methyl (*S*)-2-amino-3-((*S*)-2-oxopyrrolidin-3-yl)propanoate (20)

To a solution of **12** (46 g, 160.7 mmol) in DCM (920 mL) was added TFA (92 mL) dropwise at 0°C. The reaction solution was stirred at 25°C for 1 h. The reaction solution was concentrated in vacuo and the TFA residue was removed with toluene under reduce pressure to afford **20** (48.2g, crude, TFA salt) as a yellow oil.

^**1**^**H NMR:** 400MHz DMSO-d_6_

δ 8.58 (s, 3 H), 7.99 (s, 1 H), 4.12–4.29 (m, 1 H), 3.71–3.80 (m, 3 H), 3.10–3.26 (m, 2 H), 2.54–2.56 (m, 1 H), 2.21–2.27 (m, 1 H), 1.96–2.06 (m, 1 H), 1.84–1.94 (m, 1 H), 1.59–1.74 (m, 1 H).

#### Preparation of methyl (*S*)-2-((*S*)-2-(((benzyloxy)carbonyl)amino)-3-cyclopropylpropanamido)-3-((*S*)-2-oxopyrrolidin-3-yl)propanoate (21)

To a solution of **20** (40.02 g, 133.3 mmol, TFA salt) in DCM (450 mL) and DMF (45 mL) was added NMM (83 g, 820.4 mmol) dropwise at 0°C. The resulting mixture was stirred at 0°C for 30 min. Cbz-CpaOH (27 g, 102.6 mmol) and CMPI (65.5 g, 256.4 mmol) were added to the above mixture. The resulting mixture was stirred at 20°C for 10 h. The reaction mixture was poured into ice-water (500 mL). The aqueous phase was extracted with EtOAc (3 × 500 mL). The combined organic phases were washed with brine, dried over anhydrous Na_2_SO_4_, filtered and concentrated. The crude was purified by column chromatography (SiO_2_, petroleum ether/EtOAc = 4/1 to 0/1) to give **21** (35.4 g, 72% yield) as a yellow gum.

^**1**^**H NMR:** 400MHz DMSO-d_6_

δ 8.43 (d, *J* = 8.0 Hz, 1 H), 7.57–7.73 (m, 1 H), 7.24–7.49 (m, 6 H), 4.90–5.13 (m, 2 H), 4.22–4.46 (m, 1 H), 4.01–4.14 (m, 1 H), 3.61 (s, 3 H), 2.99–3.16 (m, 2 H), 2.20–2.41 (m, 1 H), 1.96–2.16 (m, 2 H), 1.48–1.67 (m, 3 H), 1.31–1.45 (m, 1 H), 0.76 (m, 1 H), 0.28–0.44 (m, 2 H), 0.01–0.18 (m, 2 H).

#### Preparation of benzyl ((*S*)-3-cyclopropyl-1-(((*S*)-1-hydroxy-3-((*S*)-2-oxopyrrolidin-3-yl)propan-2-yl)amino)-1-oxopropan-2-yl)carbamate (22)

A solution of LiBH_4_ (6.1 g, 278.1 mmol) in THF (200 mL) was added to a solution of **21** (40 g, 92.7 mmol) in THF (800 mL) dropwise at 0°C. EtOH (400 mL) was added and the resulting mixture was stirred at 20°C for 12 h. The reaction mixture was quenched by HCl (5% wt, 300 mL), adjust the pH ∼2 and the solvent was removed under reduced pressure. The concentrated mixture was diluted with EtOAc (2 L), washed with brine (500 mL), dried over Na_2_SO_4_, filtered and concentrated under reduced pressure to give a residue. The residue was purified by column chromatography (SiO_2_, EtOAc/MeOH = 1/0 to 10/1) to give **22** (30.9 g, 81% yield) as a white solid.

^**1**^**H NMR:** 400MHz DMSO-d_6_

δ 7.65 (d, *J* = 9.2 Hz, 1 H), 7.51 (s, 1 H), 7.24–7.45 (m, 6 H), 4.93–5.09 (m, 2 H), 4.66 (t, J = 5.6 Hz, 1 H), 3.94–4.10 (m, 1 H), 3.78 (s, 1 H), 3.37 (s, 1 H), 3.18–3.28 (m, 1 H), 2.92–3.15 (m, 2 H), 2.05–2.31 (m, 2 H), 1.65–1.83 (m, 1 H), 1.48–1.65 (m, 2 H), 1.24–1.43 (m, 2 H), 0.65–0.79 (m, 1 H), 0.27–0.45 (m, 2 H), 0.02–0.16 (m, 2 H).

#### Preparation of benzyl ((*S*)-3-cyclopropyl-1-oxo-1-(((*S*)-1-oxo-3-((*S*)-2-oxopyrrolidin-3-yl)propan-2-yl)amino)propan-2-yl)carbamate (2)

To a solution of **22** (10 g, 24.8 mmol) in DCM (300 mL) was added Dess-Martin periodinane (15.8 g, 37.2 mmol) in portions at 0°C under N_2_ atmosphere. The reaction mixture was stirred at 25°C for 3 h. The reaction mixture was cooled to 0°C, and then poured into Na_2_S_2_O_3_ (10% wt, 600 mL). The resulting mixture was stirred at 0°C for 3 min. The organic phase was washed with Na_2_S_2_O_3_ (10% wt, 400 mL), saturated NaHCO_3_ (400 mL), water (200 mL), dried over anhydrous Na_2_SO_4_, filtered and concentrated in vacuo. The crude was purified by column chromatography (SiO_2_, petroleum ether/EtOAc = 5/1 to 0/1, 0.5% DCM), and the solvent was removed under reduced pressure. A sample of the isolated solid was triturated with DCM (10 mL/g) to give **2** (25.7 mg) as a white solid.

^**1**^**H NMR:** 400MHz DMSO-d_6_

δ 9.41 (s, 1 H), 8.47 (d, *J* = 7.6 Hz, 1 H), 7.62 (s, 1 H), 7.51 (d, *J* = 8.0 Hz, 1 H), 7.08–7.41 (m, 5 H), 5.03 (d, *J* = 1.6 Hz, 2 H), 4.04–4.25 (m, 2 H), 2.95–3.20 (m, 2 H), 2.27–2.38 (m, 1 H), 2.05–2.21 (m, 1 H), 1.80–1.94 (m, 1 H), 1.52–1.70 (m, 3 H), 1.31–1.48 (m, 1 H), 0.67–0.77 (m, 1 H), 0.29–0.46 (m, 2 H), 0.01–0.19 (m, 2 H).

**LCMS (ESI+)**: m/z 402.2 (M + H)^+^.

**Figure:**
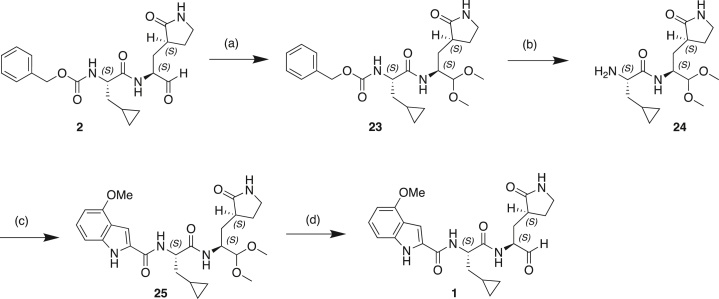

#### Scheme 4: Preparation of Compound 1

(a) (MeO)_3_CH, TsOH. H_2_O, MeOH, 70°C; (b) H_2_ gas, 10% Pd/C, MeOH, RT; (c) 4-methoxy-1H-indole-2-carboxylic acid, CMPI, NMM, DCM, DMF; (d) TFA, H_2_O, 0°C.

#### Preparation of benzyl ((*S*)-3-cyclopropyl-1-(((*S*)-1,1-dimethoxy-3-((*S*)-2-oxopyrrolidin-3-yl)propan-2-yl)amino)-1-oxopropan-2-yl)carbamate (23)

A mixture of **2** (2 g, 5 mmol), trimethoxymethane (19 g, 179.4 mmol, 19.7 mL) and TsOH·H_2_O (28.4 mg, 149.5 μmol) in MeOH (100 mL) was stirred at 70°C for 4 h. The reaction solution was concentrated in vacuo to give an oil. The oil was purified by column chromatography (SiO_2_, petroleum ether/EtOAc = 1/1 to EtOAc/MeOH = 50/1) to give **23** (2.2g, yield 60%) as a white solid.

^**1**^**H NMR:** 400MHz DMSO-d_6_

δ 7.73 (br d, J = 9.42 Hz, 1 H) 7.51 (s, 1 H) 7.24–7.45 (m, 6 H) 4.93–5.11 (m, 2 H) 4.16 (d, J = 5.48 Hz, 1 H) 3.98–4.08 (m, 1 H) 3.90 (td, J = 5.84, 3.34 Hz, 1 H) 3.17–3.32 (m, 6 H) 2.84–3.16 (m, 2 H) 2.01–2.27 (m, 2 H) 1.74–1.88 (m, 1 H) 1.23–1.61 (m, 4 H) 0.66–0.83 (m, 1 H) 0.28–0.45 (m, 2 H) 0.00–0.19 (m, 2 H).

**LCMS (ESI+)**: m/z 448.3 (M + H)^+^.

#### Preparation of (*S*)-2-amino-3-cyclopropyl-*N*-((*S*)-1,1-dimethoxy-3-((*S*)-2-oxopyrrolidin-3-yl)propan-2-yl)propanamide (24)

To a solution of **23** (1.7 g, 3.8mmol) in MeOH (36 mL) was added 10% Pd/C (500 mg, 3.8 mmol) under N_2_ atmosphere. The suspension was degassed under vacuum and purged with H_2_ gas several times. The mixture was stirred under H_2_ gas (15 psi) at 20°C for 2 h. The reaction mixture was filtered through celite and the filtrate was concentrated in vacuo to give **24** (1.2 g, crude, yield 100%) as a pale yellow oil which was used directly for the next reaction without any purification.

^**1**^**H NMR:** 400MHz DMSO-d_6_

δ 7.72 (br d, J = 9.48 Hz, 1 H) 7.54 (s, 1 H) 4.22 (d, J = 4.85 Hz, 1 H) 3.82–3.99 (m, 1 H) 3.29 (d, J = 14.55 Hz, 6 H) 3.04–3.21 (m, 3 H) 2.04–2.24 (m, 2 H) 1.66–2.02 (m, 3 H) 1.50–1.60 (m, 1 H) 1.26–1.44 (m, 3 H) 0.69–0.83 (m, 1 H) 0.29–0.45 (m, 2 H) −0.04 - 0.13 (m, 2 H).

**LCMS (ESI+)**: m/z 314.3 (M + H)^+^.

#### Preparation of *N*-((*S*)-3-cyclopropyl-1-(((*S*)-1,1-dimethoxy-3-((*S*)-2-oxopyrrolidin-3-yl)propan-2-yl)amino)-1-oxopropan-2-yl)-4-methoxy-1*H*-indole-2-carboxamide (25)

To a solution of 4-methoxy-1H-indole-2-carboxylic acid (25 mg, 130.8 μmol) in DCM (0.5 mL) were add NMM (79.4 mg, 784.6 μmol, 86.3 μL) and CMPI (83.5mg, 326.9 μmol). A solution of **24** (49.2 mg, 157 μmol) in DMF (0.1 mL) was added to above mixture. The resulting mixture was stirred at 20°C for 2 h. The mixture was filtered, and the filtrate was purified by prep-HPLC (Column: Phenomenex Gemini-NX 80 × 40 mm × 3 μm; mobile phase: [water (10 mM NH_4_HCO_3_) - MeCN]; B%: 25%–45%, 8 min) to give **25** (3.7 mg, yield 5.6%) as a white solid.

^**1**^**H NMR:** 400MHz DMSO-d_6_

δ 11.59 (s, 1 H) 8.38 (d, J = 7.87 Hz, 1 H) 7.83 (d, J = 9.42 Hz, 1 H) 7.52 (s, 1 H) 7.34 (d, J = 1.67 Hz, 1 H) 7.06–7.13 (m, 1 H) 6.96–7.03 (m, 1 H) 6.50 (d, J = 7.75 Hz, 1 H) 4.48 (td, J = 8.55, 5.19 Hz, 1 H) 4.18 (d, J = 5.60 Hz, 1 H) 3.80–4.00 (m, 4 H) 3.28 (s, 3 H) 3.23 (s, 3 H) 2.99–3.14 (m, 2 H) 2.19–2.31 (m, 1 H) 2.02–2.15 (m, 1 H) 1.78–1.91 (m, 1 H) 1.66–1.77 (m, 1 H) 1.40–1.58 (m, 2 H) 1.25–1.38 (m, 1 H) 0.74–0.89 (m, 1 H) 0.31–0.47 (m, 2 H) 0.06–0.25 (m, 2 H).

**LCMS (ESI+)**: m/z 455.3 (M + H)^+^.

#### Preparation of *N*-((*S*)-3-cyclopropyl-1-oxo-1-(((*S*)-1-oxo-3-((*S*)-2-oxopyrrolidin-3-yl)propan-2-yl)amino)propan-2-yl)-4-methoxy-1*H*-indole-2-carboxamide (1)

To a solution of **25** (130 mg, 267.2 μmol) in TFA (2 mL) was added water (2 mL) dropwise at 0°C. The solution was stirred at 0°C for 4 h. The reaction mixture was filtered and purified by prep-HPLC (Column: Phenomenex Luna C18 100 × 40 mm × 5 μm; mobile phase: [water (TFA) - MeCN]; B%: 2%–45%, 8 min) to give **1** (19.7 mg, yield 16.7%, >97% purity) as a white solid.

^**1**^**H NMR:** 400MHz DMSO-d_6_

δ 0.07–0.26 (m, 2 H) 0.33–0.47 (m, 2 H) 0.83 (br d, J = 6.17 Hz, 1 H) 1.35–2.01 (m, 5 H) 2.09–2.38 (m, 2 H) 3.00–3.18 (m, 2 H) 3.89 (s, 3 H) 4.16–4.29 (m, 1 H) 4.51–4.72 (m, 1 H) 5.71 (t, J = 6.50 Hz, 1 H) 6.50 (d, J = 7.72 Hz, 1 H) 6.97–7.04 (m, 1 H) 7.05–7.13 (m, 1 H) 7.32–7.38 (m, 1 H) 7.48–7.63 (m, 1 H) 8.35–8.63 (m, 2 H) 9.43 (s, 1 H) 11.58 (br s, 1 H).

**LCMS (ESI+)**: m/z 441.3 (M + H)^+^.

#### Scheme 5: Preparation of Compound 8

**Figure:**
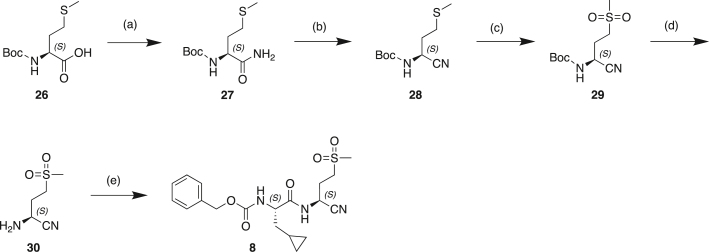

(a) ClCO_2_Et, Et_3_N, NH_4_Cl, THF, 0°C; (b) TFAA, pyridine, THF, 5°C to 20°C; (c) NaIO_4_, RuCl_3_. 2H_2_O, MeCN, H_2_O, CCl_4_, 0°C–20°C; (d) methanesulfonic acid, THF, 0°C–20°C; (e) Cbz-CpaOH, HATU, DIEA, DMF, 20°C.

#### Preparation of *tert*-butyl (*S*)-(1-amino-4-(methylthio)-1-oxobutan-2-yl)carbamate (27)

To a colorless solution of (S)-N-Boc-methionine (**26**) (19 g, 76.2 mmol) in THF (500 mL) were added ethyl chloroformate (10.5 g, 97.1 mmol) and Et_3_N (28.6mL, 205.8 mmol) at 0°C under N_2_ atmosphere. After stirring for 0.5 h at 0°C, a solution of NH_4_Cl (1 M, 114.31 mL) was added to the colorless suspension at 0°C. Four additional vials were set up as described above and all five reaction mixtures were combined. The combined mixture was quenched with water (2 L) and extracted with EtOAc (2 × 1 L). The combined organic layer was washed with brine (2 × 1 L), dried over anhydrous Na_2_SO_4_, filtered and concentrated to give **27** (95 g, yield 90%) as white powder. The reaction was used to next step without further purification.

^**1**^**H NMR:** 400MHz CDCl_3_

δ 1.44 (s, 9 H) 1.92 (dq, *J* = 14.31, 7.29 Hz, 1 H) 2.05–2.16 (m, 4 H) 2.51–2.63 (m, 2 H) 4.23–4.38 (m, 1 H) 5.35 (br s, 1 H) 5.93 (br s, 1 H) 6.43 (br s, 1 H).

**LCMS (ESI+):** m/z 248.0 (M-H)^-^.

#### Preparation of *tert*-butyl (*S*)-(1-cyano-3-(methylthio)propyl)carbamate (28)

To a solution of **27** (19 g, 76.5 mmol) in THF (320 mL) was added pyridine (31.2 g, 394.8 mmol) at 5°C under N_2_ atmosphere. TFAA (28.3 g, 134.65 mmol) was added dropwise, and the mixture was stirred for 0.5 h at 5°C. Then the reaction was stirred at 20°C for 1 h. Four additional vials were set up as described above and all five reaction mixtures were combined. The combined mixture was poured into saturated NaHCO_3_ (1 L) and maintaining the pH between 6 and 7 through the addition of solid NaHCO_3_. The inorganic solid was removed by filtration and the filter cake was washed with EtOAc (500 mL). The combined filtrate was separated, and the aqueous layer extracted with EtOAc (2 × 1 L). The combined organic layers were dried over Na_2_SO_4_, filtered and concentrated to give a brown oil. The oil was purified by column chromatography on silica gel (petroleum ether/EtOAc = 5/1) to give **28** (61 g, yield 62.3%) as colorless oil.

^**1**^**H NMR:** 400MHz CDCl_3_

δ 1.47 (br s, 9 H) 2.07–2.19 (m, 5 H) 2.59–2.71 (m, 2 H) 4.70–5.22 (m, 2 H).

**LCMS (ESI+):** m/z 230.9 (M + H)^+^.

#### Preparation of *tert*-butyl (*S*)-(1-cyano-3-(methylsulfonyl)propyl)carbamate (29)

A mixture of **28** (4 g, 17.4 mmol) in a solvent mixture of MeCN (46 mL), chloroform (46 mL) and water (68 mL) was cooled to 0°C. Then NaIO_4_ (4.5 g, 20.8 mmol) and trichlororuthenium hydrate (195.8mg, 868.3 μmol) were added. The reaction mixture was stirred at 20°C for 20 h. Four additional vials were set up as described above and all five reaction mixtures were combined. The organic layer was separated, and the aqueous layer was extracted with DCM (2 × 1 L). The combined organic layer was washed with water (1 L), dried over anhydrous Na_2_SO_4_, filtrated and concentrated under reduced pressure to afford a dark solid. The solid was purified by column chromatography on silica gel (EtOAc/MeOH = 25/1) to give **29** (11.9 g, yield 47%).

^**1**^**H NMR:** 400MHz CDCl_3_

δ 1.48 (s, 9 H) 2.42 (q, *J* = 7.42 Hz, 2 H) 3.00 (s, 3 H) 3.10–3.27 (m, 2 H) 4.77 (br s, 1 H) 5.20 (br s, 1 H).

**LCMS**: m/z: 206.9 (M-*t*Bu+H)^+^; 262.9 (M + H)^+^

#### Preparation of (*S*)-2-amino-4-(methylsulfonyl)butanenitrile (30)

To a solution of **29** (2 g, 7.6 mmol) in THF (15 mL) was added dropwise methanesulfonic acid (7.33 g, 76.2 mmol) at 0°C. The mixture was stirred for 2 h at 20°C. Four additional vials were set up as described above and all five reaction mixtures were combined. The reaction was poured into aqueous NaHCO_3_ (70 mL) at 0°C and adjusted to pH = 8 by NaHCO_3_ (solid). The mixture was extracted with DCM (3 × 100 mL). The combined organic layer was washed with water (100 mL), dried over Na_2_SO_4_, filtered and concentrated under reduced pressure to give **30** (3.7 g, yield 53.8%) as a light-yellow solid. The crude product was used in the next step without further purification.

^**1**^**H NMR:** 400MHz DMSO-d_6_.

δ 1.97–2.11 (m, 2 H) 3.01 (s, 3 H) 3.22 (t, *J* = 7.95 Hz, 2 H) 3.75–3.90 (m, 1 H).

**LCMS (ESI+):** m/z 163.2 (M + H)^+^.

#### Preparation of benzyl ((*S*)-1-(((*S*)-1-cyano-3-(methylsulfonyl)propyl)amino)-3-cyclopropyl-1-oxopropan-2-yl)carbamate (8)

To a solution of Cbz-CpaOH (130 mg, 493.2 μmol) in DMF (5 mL) was added HATU (234.4 mg, 616.5 μmol), **30** (100 mg, 616.5 μmol) and DIEA (81.3 mg, 629 μmol) at 0°C. The reaction mixture was stirred at 20°C for 3 h. Three additional vials were set up as described above and all four reaction mixtures were combined. The mixture was poured into H_2_O (40 mL) and extracted with EtOAc (3 × 20 mL). The combined organic layer was washed with brine (2 × 30 mL), dried over Na_2_SO_4_, filtered and concentrated under reduced pressure to afford a colorless crude product. The crude product was purified by prep-HPLC (Column: Phenomenex Luna C18 100 × 30 mm × 5 μm; mobile phase: [water (0.1% TFA) - MeCN]; B%: 30%–50%, 10 min) to give **8** (53 mg, yield 21%) as a white powder.

^**1**^**H NMR:** 400MHz DMSO-d_6_

δ −0.01 - 0.18 (m, 2 H) 0.31–0.47 (m, 2 H) 0.65–0.80 (m, 1 H) 1.29–1.43 (m, 1 H) 1.55–1.69 (m, 1 H) 2.13–2.32 (m, 2 H) 3.02 (s, 3 H) 3.20 (br t, *J* = 8.01 Hz, 2 H) 3.96–4.07 (m, 1 H) 4.90–5.09 (m, 3 H) 7.16–7.42 (m, 5 H) 7.60 (d, *J* = 7.46 Hz, 1 H) 8.86 (d, *J* = 7.95 Hz, 1 H).

**LCMS (ESI+)**: m/z 408.2 (M + H)^+^.

#### Scheme 6: Preparation of Compound 7

**Figure:**
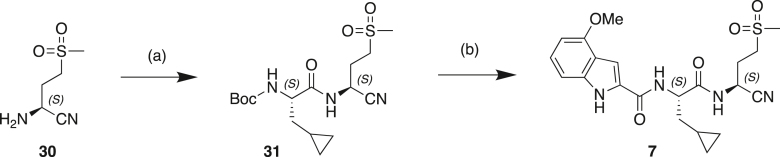

(a) Boc-CpaOH, HATU, DIEA, DMF, 20°C; (b) (1) methanesulfonic acid, THF, 0°C–20°C, (2) 4-methoxy-1H-indole-2-carboxylic acid, HATU, DIEA, DMF, 20°C.

#### Preparation of *tert*-butyl ((*S*)-1-(((*S*)-1-cyano-3-(methylsulfonyl)propyl)amino)-3-cyclopropyl-1-oxopropan-2-yl)carbamate (31)

To a solution of **30** (706.7 mg, 3.1 mmol) in DMF (5.3 mL) was added Boc-CpaOH (0.5 g, 3.1 mmol), HATU (1.2 g, 3.1 mmol) and DIEA (406.4 mg, 3.1 mmol) at 0°C. The reaction mixture was stirred at 20°C for 3 h. Three additional vials were set up as described above and all four reaction mixtures were combined. The mixture was poured into H_2_O (20 mL) and extracted with EtOAc (2 × 25 mL). The combined organic layer was washed with brine (2 × 25 mL), dried over Na_2_SO_4_, filtered and concentrated under reduced pressure to afford a colorless crude product. The crude product was purified by silica gel chromatography (petroleum ether/EtOAc = 1/2) to give **31** (241 mg, yield 21.1%) as a white powder.

^1^H NMR: 400MHz DMSO-d_6_

δ −0.03 - 0.16 (m, 2 H) 0.32–0.46 (m, 2 H) 0.71 (br s, 1 H) 1.26–1.44 (m, 10 H) 1.48–1.69 (m, 1 H) 2.12–2.29 (m, 2 H) 3.02 (s, 3 H) 3.13–3.26 (m, 2 H) 3.92 (q, *J* = 7.38 Hz, 1 H) 4.87–5.01 (m, 1 H) 7.08 (br d, *J* = 7.34 Hz, 1 H) 8.85 (br d, *J* = 7.95 Hz, 1 H).

**LCMS (ESI+):** m/z 274.2 (M-Boc+H)^+^.

#### Preparation of *N*-((*S*)-1-(((*S*)-1-cyano-3-(methylsulfonyl)propyl)amino)-3-cyclopropyl-1-oxopropan-2-yl)-4-methoxy-1*H*-indole-2-carboxamide (7)

To a solution of **31** (0.1 g, 267.8 μmol) in THF (1.06 mL) was added methanesulfonic acid (128.7 mg, 1.34 mmol, 95.3 μL) under N_2_ atmosphere at 0°C. The reaction was stirred at 20°C for 2 h under N_2_ atmosphere. Then to above mixture was added DIEA (450 mg, 3.5mmol, 606.3 μL), 4-methoxy-1H-indole-2-carboxylic acid (51.2 mg, 267.8 μmol) and HATU (101.8 mg, 267.8 μmol) in DMF (1.5 mL) at 0°C. The reaction was stirred at 20°C for 2 h. The mixture was filtered to give a colorless crude product. The crude product was purified by prep-HPLC (Column: Phenomenex Gemini-NX C18 75 × 30 mm × 3 μm; mobile phase: [water (10 mM NH_4_HCO_3_) - MeCN]; B%: 35%–65%, 8 min) to give **7** (21.2 mg, yield 17.7%) as a white powder.

^**1**^**H NMR:**^1^H NMR 400 MHz, DMSO-d_6_

δ ppm 0.01–0.13 (m, 1 H) 0.21 (br d, *J* = 9.78 Hz, 1 H) 0.42 (br d, *J* = 6.72 Hz, 2 H) 0.81 (br s, 1 H) 1.43–1.60 (m, 1 H) 1.71–1.91 (m, 1 H) 2.15–2.32 (m, 2 H) 3.01–3.07 (m, 3 H) 3.22 (br t, *J* = 8.01 Hz, 2 H) 3.89 (s, 3 H) 4.37–4.61 (m, 1 H) 4.89–5.12 (m, 1 H) 6.51 (d, *J* = 7.70 Hz, 1 H) 6.96–7.15 (m, 2 H) 7.37 (br s, 1 H) 8.54 (br d, *J* = 7.21 Hz, 1 H) 8.82–9.13 (m, 1 H) 11.58 (br s, 1 H).

**LCMS (ESI+):** m/z 447.1 (M + H)^+^.

#### Scheme 7: Preparation for Compound 4

**Figure:**
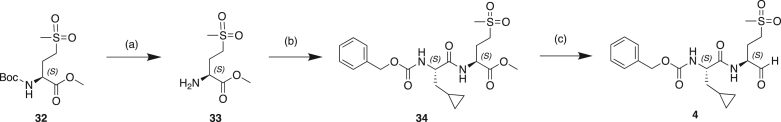

(a) TFA, DCM, 0°C–25°C; (b) Cbz-CpaOH,CMPI, NMM, DCM, DMF, 0°C–25°C; (c) DIBAl-H, −78°C.

#### Preparation of methyl (*S*)-2-amino-4-(methylsulfonyl)butanoate (33)

To a solution of **32**^48^ (5 g, 16.9 mmol) in DCM (50 mL) was added TFA (19.3 g, 169.3 mmol, 12.5 mL) in dropwise at 0°C. Then the reaction mixture was stirred at 25°C for 1 h. The reaction mixture was concentrated under reduced pressure to give **33** (6.3 g, 14.3 mmol, 84.2% yield, TFA salt) as light yellow oil.

^**1**^**H NMR:** 400MHz CD_3_OD

δ 2.50–2.30 (m, 2H), 3.04 (s, 3H), 3.39–3.32 (m, 2H), 3.88 (s, 3H), 4.28 (t, J = 6.6 Hz, 1H).

#### Preparation of methyl (*S*)-2-((*S*)-2-(((benzyloxy)carbonyl)amino)-3-cyclopropylpropanamido)-4-(methylsulfonyl)butanoate (34)

To a solution of **33** (6.3 g, 14.2 mmol, 70% purity, TFA salt) in DMF (30 mL) and DCM (10 mL) was added NMM (7.8 g, 77.5 mmol, 8.5 mL) at 0°C and the reaction mixture was stirred at 0°C for 30 min. Then Cbz-CpaOH (3.4 g, 12.9 mmol) and CMPI (8.3 g, 32.3 mmol) was added and the reaction mixture was stirred at 25°C for 12 h. The reaction mixture was poured into water (50 mL) and extracted with EtOAc (50 mL × 3). The combined organic layer was dried over Na_2_SO_4_, filtered and concentrated under reduced pressure to give a residue. The residue was triturated with petroleum ether/EtOAc (1/1, 20 mL) at 25°C for 30 min and filtered to give **34** (4.1 g, 72.1% yield) as light yellow solid.

^**1**^**H NMR:** 400MHz CD_3_OD

δ 0.19–0.07 (m, 2H), 0.54–0.41 (m, 2H), 0.80 (s, 1H), 1.67–1.53 (m, 2H), 2.23–2.07 (m, 1H), 2.37 (br d, J = 6.4 Hz, 1H), 2.94 (s, 3H), 3.27–3.16 (m, 2H), 3.73 (s, 3H), 4.15 (t, J = 6.5 Hz, 1H), 4.56 (s, 1H), 4.64 (dd, J = 4.1, 8.9 Hz, 1H), 5.09 (s, 2H), 7.39–7.24 (m, 5H).

^**1**^**H NMR:** 400MHz DMSO-d_6_

δ 0.16–0.00 (m, 2H), 0.45–0.30 (m, 2H), 0.75 (br s, 1H), 1.46–1.35 (m, 1H), 1.58–1.48 (m, 1H), 2.08–1.94 (m, 1H), 2.23–2.11 (m, 1H), 2.97 (s, 3H), 3.25–3.05 (m, 2H), 3.63 (s, 3H), 4.07 (dt, J = 5.6, 8.3 Hz, 1H), 4.48–4.38 (m, 1H), 5.02 (d, J = 1.8 Hz, 2H), 7.39–7.27 (m, 4H), 7.45 (d, J = 7.9 Hz, 1H), 8.41 (d, J = 7.9 Hz, 1H).

**LCMS (ESI+)**: m/z 441.1 (M + H)^+^.

#### Preparation of benzyl ((*S*)-3-cyclopropyl-1-(((*S*)-4-(methylsulfonyl)-1-oxobutan-2-yl)amino)-1-oxopropan-2-yl)carbamate (4)

To a solution of **34** (3 g, 6.8 mmol) in DCM (30 mL) was added diisobutylaluminum hydride (1 M, 23.8 mL) at −70°C. Then the reaction mixture was stirred at −78°C for 3 h. The reaction mixture was quenched by addition of cold MeOH (20 mL) at −60°C. Then the reaction mixture was warmed to 20°C, diluted with EtOAc (100 mL), filtered and the filtrate was concentrated under reduced pressure to give a residue. The residue was triturated with MeCN/DCM (1/1, 30 mL) at 25°C for 30 min and filtered to give a crude product (1.1 g) as off-white solid. Then 160 mg of the crude product was triturated with MeCN and filtered to give **4** (120 mg) as white solid.

^**1**^**H NMR:** 400MHz DMSO-d_6_

δ 0.18–0.01 (m, 2H), 0.48–0.29 (m, 2H), 0.76 (br s, 1H), 1.48–1.34 (m, 1H), 1.67–1.51 (m, 1H), 1.94–1.78 (m, 1H), 2.28–2.11 (m, 1H), 3.00–2.91 (m, 3H), 3.19–3.02 (m, 2H), 4.12–4.02 (m, 1H), 4.28–4.18 (m, 1H), 5.12–4.97 (m, 2H), 7.43–7.27 (m, 5H), 7.57 (d, J = 7.3 Hz, 1H), 8.55 (d, J = 7.5 Hz, 1H), 9.39 (s, 1H).

**LCMS (ESI+)**: m/z 411.1 (M + H)^+^.

#### Scheme 8: Preparation of Compound 3

**Figure:**
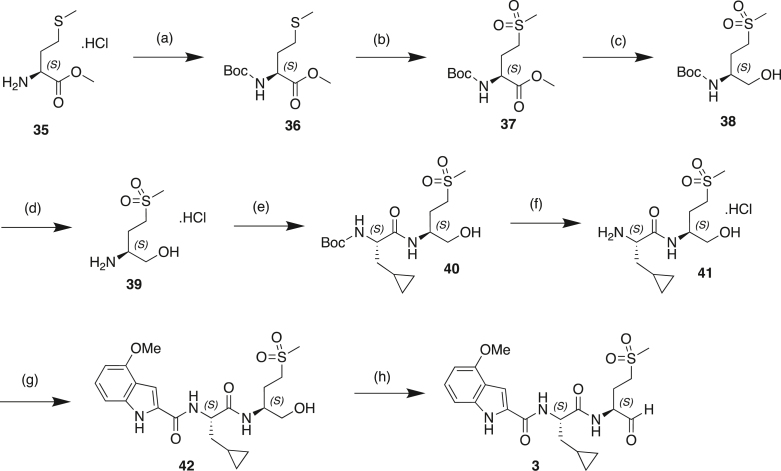

(a) (Boc)_2_O, Et_3_N, DCM, 0°C; (b) methyltrioxorhenium(VII), EtOH, AcOH, 30% H_2_O_2_, 3°C; (c) LiBH_4_, THF, −75°C to RT; (d) 4N HCl/Dioxane, 50°C; (e) Boc-CpaOH DCHA salt, HATU, Et_3_N, DMF, 0°C; (f) 4N HCl/Dioxane, 50°C; (g) 4-methoxy-1H-indole-2-carboxylic acid, EDCI, HOAT, Et_3_N, DMF, 0°C; (h) DMSO, DCM, DIEA, SO_3_-py complex, 5°C to RT.

#### Preparation of Boc-(S)-methionine methyl ester (36)

A mixture of methionine methyl ester hydrochloride (**35**) (10 g, 50.1 mmol) in DCM (100 mL) was cooled to 0°C, treated dropwise with Et_3_N (17.1 mL, 123 mmol) over 10 min, keeping the internal temperature below 2°C, treated dropwise with a solution of (Boc)_2_O (11.6 mL, 50.1 mmol) in DCM (25 mL) over 20 min, keeping the internal temperature below 2°C, and then stirred overnight at ambient temperature. The next morning solid was present in the reaction mixture. The mixture was treated with methyl *tert*-butyl ether (100 mL) and stirred for 10 min. The solids were collected by filtration, washed with methyl *tert*-butyl ether, and discarded. The filtrate was concentrated. The residue was dissolved in a small amount of DCM and transferred to a Teledyne Isco 65g load cartridge containing 25 g of silica gel wet with heptanes atop a pre-equilibrated 80 g column. Chromatography on a CombiFlash Nextgen 300 Plus eluting with 10% (2.5 min), 10–50% (over 22.5 min) EtOAc in heptanes provided **36** (12.1 g, 45.9 mmol, 92% yield).

^**1**^**H NMR:** 400 MHz DMSO-d_6_

δ 7.28 (d, J = 7.9 Hz, 1H), 4.10 (td, J = 8.4, 5.1 Hz, 1H), 3.63 (s, 3H), 2.49–2.41 (m, 2H), 2.03 (s, 3H), 1.94–1.78 (m, 2H), 1.38 (s, 9H).

**LCMS**: m/z: 163.9 (M-Boc+H)^+^

#### Preparation of methyl (*S*)-2-((*tert*-butoxycarbonyl)amino)-4-(methylsulfonyl)butanoate (37)^49^

A 0°C solution of **36** (5 g, 19 mmol) and methyltrioxorhenium(VII) (0.047 g, 0.19 mmol) in a 9:1 mixture of EtOH (45 mL) and AcOH (5 mL) was treated dropwise with aqueous 30% H_2_O_2_ (4.9 mL, 47.5 mmol) over 40 min keeping the internal temperature below 3°C. The mixture was slowly warmed to ambient temperature for 2.5 h. The reaction was stirred for an additional 45 min and was then poured into a 2 L Erlenmeyer flask containing a vigorously stirred mixture of EtOAc (500 mL), aqueous 1M sodium thiosulfate pentahydrate (76 mL, 76 mmol), and aqueous saturated sodium bicarbonate (80 mL) and stirred for 15 min. The layers were separated, and the aqueous layer was extracted with EtOAc (2 x 100 mL). The combined EtOAc layers were washed with brine (2 x 25 mL), dried over magnesium sulfate, filtered, and concentrated to provide **37** (5.3 g, 95% yield), as a white solid.

^**1**^**H NMR:** 400 MHz DMSO-d_6_

δ 7.38 (d, J = 8.1 Hz, 1H), 4.13 (td, J = 8.9, 4.9 Hz, 1H), 3.65 (s, 3H), 3.21 (ddd, J = 13.8, 10.9, 5.2 Hz, 1H), 3.15–3.03 (m, 1H), 2.97 (s, 3H), 2.18–2.08 (m, 1H), 2.04–1.91 (m, 1H), 1.39 (s, 9H).

**LCMS**: m/z: 196.1 (M-Boc+H)^+^

#### Preparation of *tert*-butyl (*S*)-(1-hydroxy-4-(methylsulfonyl)butan-2-yl)carbamate (38)

A solution of **37** (993 mg, 3.4 mmol) in THF (14 mL) was chilled to −75°C and treated all at once with solid lithium borohydride (110 mg, 5 mmol). The reaction mixture was stirred overnight at ambient temperature. A resulting homogeneous white mixture was chilled in an ice bath and treated dropwise with water (0.7 mL, 36.1 mmol) over 5 min. The mixture was stirred at ambient temperature for 15 min. The mixture was diluted with EtOAc (50 mL), stirred for 1 min, then treated all at once with magnesium sulfate (869 mg, 72.2 mmol), some bubbling occurred at this point. The mixture was stirred at ambient temperature for 10 min. The mixture was filtered to remove the solids and rinsed with EtOAc (2 x 25 mL). The filtrate was concentrated to provide **38** (992.5 mg) as a colorless oil which solidified upon cooling.

^**1**^**H NMR:** 400 MHz CDCl_3_

δ 4.94 (d, J = 8.6 Hz, 1H), 3.80–3.63 (m, 3H), 3.14 (t, J = 8.0 Hz, 2H), 2.93 (s, 3H), 2.32 (t, J = 5.4 Hz, 1H), 2.18–2.01 (m, 2H), 1.45 (s, 9H).

**LCMS**: m/z: 212.2 (M-*t*Bu+H)^+^; 167.9 (M-Boc+H)^+^

#### Preparation of (*S*)-2-amino-4-(methylsulfonyl)butan-1-ol (39)

A 4N hydrogen chloride (12 mL, 48 mmol) solution in dioxane was added to a solution of **38** (992.5 mg, 3.7 mmol) in dioxane (12 mL). The solution was heated at 50°C. A white precipitate formed within 5 min. The mixture was stirred for 1 h and concentrated to a white slurry. EtOAc was added and the resulting solid was collected by filtration, rinsed with EtOAc, and freeze-dried overnight on a lyophilizer to give **39** (652 mg, 3.2 mmol, 86% yield) as a white solid.

**LCMS**: m/z 168.0.

#### Preparation of *tert*-butyl ((*S*)-3-cyclopropyl-1-(((*S*)-1-hydroxy-4-(methylsulfonyl)butan-2-yl)amino)-1-oxopropan-2-yl)carbamate (40)

A mixture of **39** (350 mg, 1.72 mmol) in DMF (8mL) was cooled to 0°C, treated with HATU (849 mg, 2.2 mmol), Boc-CpaOH (2.2 mmol) and Et_3_N (0.9 mL, 6.5 mmol), and stirred the reaction mixture at 0°C. After 1 h, the mixture was quenched with water (50 mL) aqueous 1M citric acid (6 mL) was added and extracted with DCM (2 x 50 mL), filtered through a Biotage Isolute phase separator, and concentrated to a colorless oil. The oil was diluted with DCM. Chromatography on a Biotage Sfar HC 50g column, eluting with 100% DCM for 2 CV (120 mL/min), 0–100% EtOAc in DCM for 15 CV (120 mL/min), and 0–100% 1:3 ethanol: EtOAc in EtOAc for 10 CV (120 mL/min) provided **40** (507 mg, 1.34 mmol, 78% yield) as a colorless oil which solidified upon standing.

^**1**^**H NMR:** 600 MHz CDCl_3_

δ 6.68 (d, J = 8.5 Hz, 1H), 5.18 (d, J = 6.9 Hz, 1H), 4.11–4.02 (m, 2H), 3.70 (qd, J = 11.1, 4.1 Hz, 2H), 3.15 (t, J = 7.8 Hz, 2H), 2.94 (s, 3H), 2.19–2.08 (m, 2H), 1.64 (dh, J = 28.1, 6.9 Hz, 2H), 1.45 (s, 9H), 0.75–0.67 (m, 1H), 0.54–0.47 (m, 2H), 0.16–0.10 (m, 2H).

**LCMS:** m/z 279.3 (M-Boc+H)^+^; 322.3(M-*t*Bu+H)^+^; 379.4 (M + H)^+^

#### Preparation of (*S*)-2-amino-3-cyclopropyl-*N*-((*S*)-1-hydroxy-4-(methylsulfonyl)butan-2-yl)propenamide hydrochloride (41)

A 4N hydrogen chloride solution (3.5 mL, 14 mmol) in dioxane was added to a solution of **40** (496.5 mg, 1.3 mmol) in dioxane (6.5 mL). The solution was heated at 50°C. A white precipitate formed within 20 min. The mixture was stirred for 1 h and concentrated to a white slurry. EtOAc was added, and the resulting solid collected by filtration, rinsed with EtOAc, and freeze-dried overnight on a lyophilizer to give **41** (352 mg, 1.12 mmol, 85% yield) as a white solid.

**LCMS**: m/z 279.3 (M + H)^+^.

#### Preparation of *N*-((*S*)-3-cyclopropyl-1-(((*S*)-1-hydroxy-4-(methylsulfonyl)butan-2-yl)amino)-1-oxopropan-2-yl)-4-methoxy-1*H*-indole-2-carboxamide (42)

A solution of **41** (352 mg, 1.12 mmol), 4-methoxy-1H-indole-2-carboxylic acid (235 mg, 1.23 mmol), EDCl (257 mg, 1.34 mmol), and HOAt) (152 mg, 1.12 mmol) in DMF (7 mL) was chilled to 0°C, and then treated with Et_3_N (0.5 mL, 3.6 mmol). The turbid solution was stirred overnight at ambient temperature. The reaction mixture was quenched with water (50 mL), aqueous 1M citric acid (5 mL) was added, extracted with DCM (2 x 50 mL), filtered through Biotage Isolute phase separator, and concentrated to a yellow oil. The oil was diluted with DCM. Chromatography on a Biotage Sfar HC 25g column, eluting with 100% DCM for 3 CV (80 mL/min), then 0–20% MeOH in DCM for 20 CV (80 mL/min). The fractions with product were combined and concentrated and diluted with EtOAc and chromatographed on a Biotage Sfar HC 25g column, eluting with 100% EtOAc for 3 CV (80 mL/min), then 0–100% 1:3 EtOH:EtOAc in EtOAc for 20 CV (80 mL/min)provided **42** (390 mg, 0.86 mmol, 77% yield) as a white solid.

^**1**^**H NMR:** 500 MHz CDCl_3_

δ 10.13 (d, J = 2.3 Hz, 1H), 7.65 (d, J = 8.7 Hz, 1H), 7.21 (d, J = 7.7 Hz, 1H), 7.17 (dd, J = 8.3, 7.7 Hz, 1H), 7.14 (dd, J = 2.3, 0.9 Hz, 1H), 7.01 (dt, J = 8.3, 0.8 Hz, 1H), 6.48 (dd, J = 7.9, 0.6 Hz, 1H), 4.74 (q, J = 7.1 Hz, 1H), 4.13–4.04 (m, 1H), 3.90 (s, 3H), 3.71 (dd, J = 6.3, 4.0 Hz, 2H), 3.57 (t, J = 6.2 Hz, 1H), 3.15–3.03 (m, 2H), 2.72 (s, 3H), 2.16–2.01 (m, 2H), 1.75 (td, J = 7.0, 3.1 Hz, 2H), 0.76–0.67 (m, 1H), 0.46–0.38 (m, 2H), 0.12–0.05 (m, 2H).

**LCMS**: m/z 452.4 (M + H)^+^.

#### Preparation of *N*-((*S*)-3-cyclopropyl-1-(((*S*)-4-(methylsulfonyl)-1-oxobutan-2-yl)amino)-1-oxopropan-2-yl)-4-methoxy-1*H*-indole-2-carboxamide (3)

To a solution of **42** (130 mg, 0.29 mmol) in DMSO (1 mL) was added DCM (1 mL) and DIEA (0.15 mL, 0.86 mmol) and the solution was chilled to 1°C. A solution of SO_3_-py complex (137 mg, 0.86 mmol) in DMSO (1 mL) and DCM (1 mL) was added dropwise to the chilled solution and reaction temperature maintained below 5°C. The ice bath was removed after 15 min and the reaction was stirred at ambient temperature for 30 min. The reaction mixture was quenched with 18 mL water, and 2 mL aqueous 1M citric acid was added. The mixture was extracted with DCM (2 x 20 mL) and filtered through a Biotage Isolute phase separator. A translucent solid which was product was collected and rinsed with DCM (20 mL), transferred to a vial with EtOAc and concentrated. MeCN was added. The mixture concentrated and freeze-dried on the lyophilizer to provide **3** (70 mg, 0.16 mmol, 54. % yield) as a white solid.

^**1**^**H NMR:** 600 MHz DMSO-d_6_

δ 11.56 (d, *J* = 2.3 Hz, 1H), 9.41 (s, 1H), 8.61 (d, *J* = 7.6 Hz, 1H), 8.49 (d, *J* = 7.6 Hz, 1H), 7.37 (dd, *J* = 2.3, 0.9 Hz, 1H), 7.13–7.07 (m, 1H), 7.02 (dd, *J* = 8.3, 0.9 Hz, 1H), 6.51 (dd, *J* = 7.8, 0.7 Hz, 1H), 4.54 (ddd, *J* = 9.3, 7.6, 5.2 Hz, 1H), 4.24 (ddd, *J* = 9.3, 7.5, 4.6 Hz, 1H), 3.89 (s, 3H), 3.16 (ddd, *J* = 13.9, 11.1, 5.4 Hz, 1H), 3.10 (ddd, *J* = 13.9, 11.4, 4.9 Hz, 1H), 2.99 (s, 3H), 2.24 (ddt, *J* = 13.7, 11.4, 5.0 Hz, 1H), 1.92 (dddd, *J* = 13.9, 11.1, 9.3, 4.8 Hz, 1H), 1.80 (ddd, *J* = 13.8, 9.3, 6.4 Hz, 1H), 1.61 (ddd, *J* = 13.4, 7.6, 5.3 Hz, 1H), 0.88–0.80 (m, 1H), 0.47–0.36 (m, 2H), 0.25–0.18 (m, 1H), 0.14–0.08 (m, 1H).

**LCMS**: m/z 450.3 (M + H)

#### Scheme 9: Preparation of 45

**Figure:**
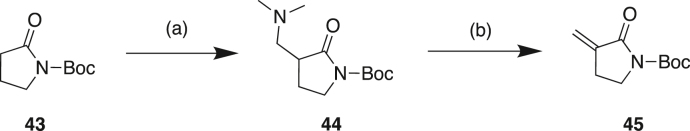

(a) N-methyl-N-methylenemethanaminium iodide, LiHMDS, THF; (b) 3-bromoprop-1-ene, Na_2_CO_3_, EtOH.

#### Preparation of *tert*-butyl 3-((dimethylamino)methyl)-2-oxopyrrolidine-1-carboxylate (44)

To a solution of N-Boc-pyrrolidone (**43**) (10 g, 54 mmol, 9.2 mL) in THF (150 mL) was added lithium bis(trimethylsilyl)amide (1 M, 70.2 mL) dropwise at −60°C under N_2_. The yellow solution was stirred at −60°C for 1 h. Then N-methyl-N-methylenemethanaminium iodide (15 g, 81 mmol) was added in one portion and the resulting mixture was stirred at −60°C for 2.5 h. The resulting yellow suspension was warmed to 0°C and stirred for 0.5 h. Then the mixture was quenched with saturated NH_4_Cl aqueous (400 mL) and extracted with EtOAc (3 × 300 mL). The combined organic phases were dried over Na_2_SO_4_, filtered, and the filtrate was concentrated under reduced pressure to give **44** (13 g, 99.3% yield) as a red oil. The crude product was used directly to next step without further purification.

^**1**^**H NMR** 400 MHz, CDCl_3_

δ ppm 3.56–3.84 (m, 2 H), 2.68–2.84 (m, 1 H), 2.39–2.60 (m, 2 H), 2.32 (s, 3 H), 2.27 (s, 3 H), 1.51–1.54 (m, 9 H), 1.42–1.49 (m, 2 H).

#### Preparation of *tert*-butyl 3-methylene-2-oxopyrrolidine-1-carboxylate (45)

To a solution of **44** (13 g, 53.6 mmol) in EtOH (200 mL) were added allyl bromide (51.9 g, 429.2 mmol, 37.1 mL) and Na_2_CO_3_ (34.1 g, 321.9 mmol). The resulting yellow suspension was stirred at 25°C for 18 h. The resulting white suspension was filtered, and the filtrate was concentrated under reduced pressure. The crude was purified by column chromatography on silica gel, eluting with 10% EtOAc in petroleum ether to give **45** (5 g, 47% yield) as a yellow oil.

^**1**^**H NMR** 400 MHz, CDCl_3_

δ ppm 6.09 (t, *J* = 2.81 Hz, 1 H), 5.38 (t, *J* = 2.44 Hz, 1 H), 3.64 (t, *J* = 7.25 Hz, 2 H), 2.65 (tt, *J* = 7.22, 2.66 Hz, 2 H), 1.45 (s, 9 H).

#### Scheme 10: Preparation of ^13^C–5

**Figure:**
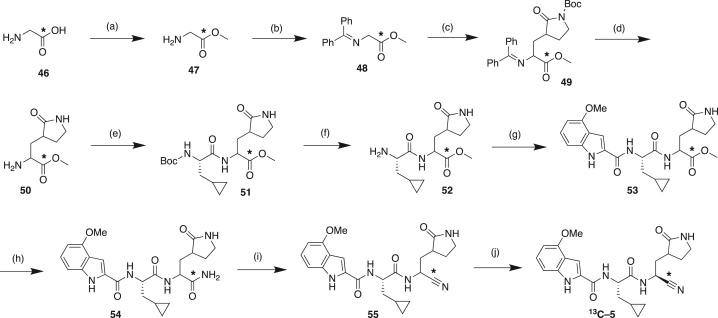

∗ indicates ^13^C-label. (a) SOCl_2_, MeOH; (b) benzophenone, TsOH, DIEA, toluene; (c) **45**, DBU, LiBr, THF, 25°C; (d) TFA, DCM; (e) Boc-CpaOH, HATU, HOAt, DMF, DCM; (f) TFA, DCM; (g) 4-methoxy-1H-indole-2-carboxylic acid, HATU, HOAt, DMF, DCM; (h) NH_3_, MeOH; (i) Burgess reagent, DCM; (j) SFC separation.

#### Preparation of methyl glycinate-1-^13^*C* (47)

To a white suspension of glycine-1-^13^C (**46**) (6 g, 80 mmol) in MeOH (60 mL) was added thionyl chloride (17.1 g, 144 mmol, 10.4 mL) dropwise at 0°C. The colorless solution was stirred 25°C for 2 h. The resulting white slurry was heated at 70°C for 2 h. After cooling, the resulting white suspension was concentrated. The off-white solid was triturated with methyl *tert*-butyl ether (20 mL), filtered and the filter cake was dried in vacuo to give **47** (9.9 g, 98.7% yield, HCl salt) as a white solid.

^**1**^**H NMR** 400 MHz, D_2_O

δ ppm 3.91 (d, *J* = 6.50 Hz, 2 H), 3.81 (d, *J* = 3.88 Hz, 3 H).

#### Preparation of methyl 2-((diphenylmethylene)amino)acetate-1-^13^*C* (48)

A white suspension of **47** (9.9 g, 78.9 mmol, HCl salt), benzophenone (28.7 g, 158 mmol) and 4-methylbenzenesulfonic acid (1.4 g, 7.9 mmol) in toluene (90 mL) was stirred at 130°C. The water was removed by Dean-Stark trap. Then N-ethyl-N-isopropylpropan-2-amine (21.4 g, 165.6 mmol, 28.8 mL) was added dropwise at 130°C over 6 h. After cooling, the resulting mixture was quenched with water (100 mL) and extracted with EtOAc (3 × 100 mL). The combined organic phases were washed with brine (100), dried over Na_2_SO_4_, filtered, and the filtrate was concentrated at 45°C under reduced pressure. The crude was purified by column chromatography on silica gel, eluting with 9–15% EtOAc in petroleum ether to give **48** (7.4 g, 37% yield) as a pale yellow oil.

^**1**^**H NMR** 400 MHz, CDCl_3_

δ ppm 7.67 (d, *J* = 7.13 Hz, 2 H), 7.45–7.52 (m, 3 H), 7.39–7.44 (m, 1 H), 7.32–7.38 (m, 2 H), 7.16–7.22 (m, 2 H), 4.23 (d, *J* = 7.00 Hz, 2 H), 3.76 (d, *J* = 3.88 Hz, 3 H).

#### Preparation of *tert*-butyl 3-(2-((diphenylmethylene)amino)-3-methoxy-3-oxopropyl-3-^13^*C*)-2-oxopyrrolidine-1-carboxylate (49)

To a solution of **48** (7.4 g, 29.2 mmol) and **45** (7.5 g, 38 mmol) in THF (150 mL) were added DBU (890 mg, 5.8 mmol, 881 μL) and lithium bromide (2.54 g, 29.2 mmol, 733.3 μL) at 25°C. The mixture was stirred at 25°C for 15 h. The solution was concentrated under reduced pressure to dryness. The crude product was purified by column chromatography on silica gel, eluting with 25% EtOAc in petroleum ether to give **49** (5.6 g, 42.6% yield) as a colorless gum.

^**1**^**H NMR** 400 MHz, CD_3_OD

δ ppm 7.59 (d, *J* = 7.50 Hz, 2 H), 7.48–7.55 (m, 3 H), 7.41–7.46 (m, 1 H), 7.33–7.39 (m, 2 H), 7.18–7.26 (m, 2 H), 4.14–4.37 (m, 1 H), 3.66–3.75 (m, 4 H), 3.45–3.58 (m, 1 H), 2.45–2.72 (m, 2 H), 1.85–2.12 (m, 2 H), 1.46–1.54 (m, 9 H).

**LCMS (ESI+)**: m/z 452.5 (M + H)^+^.

#### Preparation of *tert*-butyl 3-(2-amino-3-methoxy-3-oxopropyl-3-^13^*C*)-2-oxopyrrolidine-1-carboxylate (50)

To a colorless solution of **49** (5.6 g, 12.4 mmol) in DCM (115 mL) was added TFA (14.2 g, 124.3 mmol, 9.2mL) at 0°C. The resulting solution was stirred at 25°C for 1 h. The solution was poured in water (80 mL) and extracted with DCM (3 × 50 mL). The aqueous layer was lyophilized to give **50** (3.5g, 93.3% yield, TFA salt) as a yellow gum. The product was used directly to next step without further purification.

^**1**^**H NMR** 400 MHz, CD_3_OD

δ ppm 4.17–4.36 (m, 1 H), 3.86 (dd, *J* = 6.42, 3.85 Hz, 3 H), 3.34–3.42 (m, 2 H), 2.52–2.84 (m, 1 H), 2.39 (m, 1 H), 2.16–2.30 (m, 1 H), 1.95–2.14 (m, 1 H), 1.79–1.94 (m, 1 H).

#### Preparation of methyl 2-((*S*)-2-((*tert*-butoxycarbonyl)amino)-3-cyclopropylpropanamido)-3-(2-oxopyrrolidin-3-yl)propanoate-1-^13^*C* (51)

To a mixture of Boc-CpaOH (1.7 g, 7.36 mmol), HATU (2.2 g, 5.7 mmol) and HOAt (154 mg, 1.1 mmol, 158.42 μL) in DMF (17 mL) was added DIEA (2.4 g, 18.67 mmol, 3.3 mL). The mixture was stirred at 25°C for 15 min. Then a mixture of **50** (1.7 g, 5.7 mmol, TFA salt) in DCM (17 mL) was added to the reaction mixture and the mixture was stirred at 25°C for 15 h. The mixture was quenched with water (50 mL) and extracted with EtOAc (3 × 50 mL). The combined organic phase was washed with saturated NaHCO_3_ (50 mL) and then HCl (1 M, 50 mL). The combined organic phase was washed with brine (100 mL), dried over Na_2_SO_4_, filtered, and the filtrate was concentrated under reduced pressure to dryness. The crude was purified by column chromatography on silica gel, eluting with EtOAc to give **51** (1.0 g, 46% yield) as a white solid.

^**1**^**H NMR** 400 MHz, CD_3_OD

δ ppm 4.45–4.67 (m, 1 H), 4.05–4.16 (m, 1 H), 3.68–3.77 (m, 3 H), 3.35 (br d, *J* = 3.30 Hz, 1 H), 3.25 (br s, 1 H), 2.12–2.64 (m, 3 H), 1.52–1.89 (m, 4 H), 1.35–1.52 (m, 9 H), 0.72–0.88 (m, 1 H), 0.48 (br t, *J* = 7.76 Hz, 2 H), 0.06–0.20 (m, 2 H).

**LCMS (ESI+)**: m/z 399.4 (M + H)^+^

#### Preparation of methyl 2-((*S*)-2-amino-3-cyclopropylpropanamido)-3-(2-oxopyrrolidin-3-yl)propanoate-1-^13^*C* (52)

To a solution of **51** (1 g, 2.5 mmol) in DCM (30 mL) was added TFA (30 mL) at 25°C. The resulting mixture was stirred at 25°C for 1.5 h. The mixture was concentrated under reduced pressure to give **52** (1. g, 100% yield, TFA salt) as a colorless oil. The crude product was used directly to next step without further purification.

^**1**^**H NMR** 400 MHz, CD_3_OD

δ ppm 4.45–4.74 (m, 1 H), 3.90–3.98 (m, 1 H), 3.70–3.78 (m, 3 H), 3.32–3.40 (m, 2 H), 2.09–2.55 (m, 3 H), 1.69–1.94 (m, 4 H), 0.72–0.90 (m, 1 H), 0.53–0.64 (m, 2 H), 0.16–0.27 (m, 2 H).

#### Preparation of methyl 2-((*S*)-3-cyclopropyl-2-(4-methoxy-1*H*-indole-2-carboxamido)propanamido)-3-(2-oxopyrrolidin-3-yl)propanoate-1-^13^*C* (53)

To a mixture of 4-methoxy-1H-indole-2-carboxylic acid (622 mg, 3.3 mmol), HATU (952 mg, 2.5 mmol) and HOAt (68 mg, 500.8 μmol, 70 μL) in DMF (20 mL) was added DIEA (1.1 g, 8.3 mmol, 1.4 mL) at 25°C. The mixture was stirred at 25°C for 0.5 h. Then a solution of **52** (1. g, 2.5 mmol, TFA salt) in DCM (20 mL) was added to the reaction mixture and the mixture was stirred at 25°C for 12 h. The resulting mixture was quenched with water (50 mL) and extracted with EtOAc (3 × 100 mL). The combined organic phase was washed with saturated NaHCO_3_ (50 mL), and HCl (1 M, 50 mL). The organic layer was washed with brine (100 mL), dried over Na_2_SO_4_, filtered, and the filtrate was concentrated under reduced pressure. The crude was purified by column chromatography on silica gel, eluting with 5% MeOH in EtOAc to give **53** (0.64 g, 54.3% yield) as a yellow solid.

^**1**^**H NMR** 400 MHz, CD_3_OD

δ ppm 7.26 (s, 1 H), 7.12–7.18 (m, 1 H), 7.03 (d, *J* = 8.00 Hz, 1 H), 6.51 (d, *J* = 7.75 Hz, 1 H), 4.49–4.72 (m, 2 H), 3.93 (s, 3 H), 3.69–3.74 (m, 3 H), 3.22–3.30 (m, 2 H), 2.09–2.69 (m, 3 H), 1.67–1.91 (m, 4 H), 0.88 (ddt, *J* = 14.79, 7.72, 3.75, 3.75 Hz, 1 H), 0.44–0.57 (m, 2 H), 0.13–0.26 (m, 2 H).

**LCMS (ESI+)**: m/z 472.5 (M + H)^+^.

#### Preparation of *N*-((2*S*)-1-((1-amino-1-oxo-3-(2-oxopyrrolidin-3-yl)propan-2-yl-1-^13^*C*)amino)-3-cyclopropyl-1-oxopropan-2-yl)-4-methoxy-1*H*-indole-2-carboxamide (54)

NH_3_ gas was bubbled into a solution of **53** (0.64 g, 1.4 mmol) in MeOH (60 mL) at −40°C for 30 min. Then the mixture was stirred at 25°C for 15 h. The resulting solution was concentrated under reduced pressure to give **54** (0.6 g, 96.8% yield) as an off-white solid. The crude product was used directly to next step without further purification.

^**1**^**H NMR** 400 MHz, CD_3_OD

δ ppm 7.23–7.31 (m, 1 H), 7.12–7.18 (m, 1 H), 6.99–7.05 (m, 1 H), 6.51 (d, *J* = 7.63 Hz, 1 H), 4.37–4.62 (m, 2 H), 3.93 (s, 3 H), 3.19–3.30 (m, 2 H), 2.07–2.64 (m, 3 H), 1.67–1.94 (m, 4 H), 0.76–0.96 (m, 1 H), 0.42–0.59 (m, 2 H), 0.12–0.26 (m, 2 H).

**LCMS (ESI+)**: m/z 457.5 (M + H)^+^.

#### Preparation of *N*-((2*S*)-1-((1-(cyano-^13^*C*)-2-(2-oxopyrrolidin-3-yl)ethyl)amino)-3-cyclopropyl-1-oxopropan-2-yl)-4-methoxy-1*H*-indole-2-carboxamide (55)

To a mixture of **54** (500 mg, 1.1mmol) in DCM (50 mL) was added Burgess reagent (863 mg, 3.6 mmol) at 25°C. The reaction mixture was stirred at 25°C for 10 h. The yellow mixture was quenched with water (3 mL) and then concentrated by evaporation. The residue was diluted with MeCN and purified by prep-HPLC (column: Phenomenex Luna C18 100∗40mm∗5 μm; mobile phase: [water (0.1% TFA)-MeCN]; gradient:25%–55% B over 8 min) to give **55** (337 mg, 70.2% yield) as a white solid.

#### Preparation of *N*-((*S*)-1-(((*S*)-1-(cyano-^13^*C*)-2-((*S*)-2-oxopyrrolidin-3-yl)ethyl)amino)-3-cyclopropyl-1-oxopropan-2-yl)-4-methoxy-1*H*-indole-2-carboxamide (^13^C–5)

A solution of **55** (365 mg, 834.3 μmol) in MeOH (1 mL) was separated by Chiral-SFC under neutral condition to give ^**13**^**C-5** (61 mg, 16.7% yield) as a white solid.

##### Chiral SFC method

Instrument: Waters SFC80 preparative SFC; Column: DAICEL CHIRALCEL OX (250mm∗30mm,10 μm); Mobile phase: A for CO_2_ and B for EtOH; Gradient: B% = 40% isocratic elution mode; Flow rate: 70 g/min; Wavelength:220 nm; Column temperature: 40°C; System back pressure: 100 bar.

^**1**^**H NMR** 400 MHz, CD_3_OD

δ ppm 7.26 (s, 1 H), 7.12–7.17 (m, 1 H), 7.02 (d, *J* = 8.16 Hz, 1 H), 6.51 (d, *J* = 7.65 Hz, 1 H), 5.07 (td, *J* = 9.60, 5.77 Hz, 1 H), 4.54 (t, *J* = 7.40 Hz, 1 H), 3.93 (s, 3 H), 3.26–3.30 (m, 2 H), 2.65 (qd, *J* = 9.26, 5.21 Hz, 1 H), 2.21–2.44 (m, 2 H), 1.75–1.96 (m, 3 H), 1.66 (dt, *J* = 14.15, 7.29 Hz, 1 H), 0.79–0.91 (m, 1 H), 0.54 (d, *J* = 8.03 Hz, 2 H), 0.13–0.26 (m, 2 H).

**LCMS (ESI+)**: m/z 439.5 (M + H)^+^.

#### Scheme 11: Preparation of ^13^C–1

**Figure:**
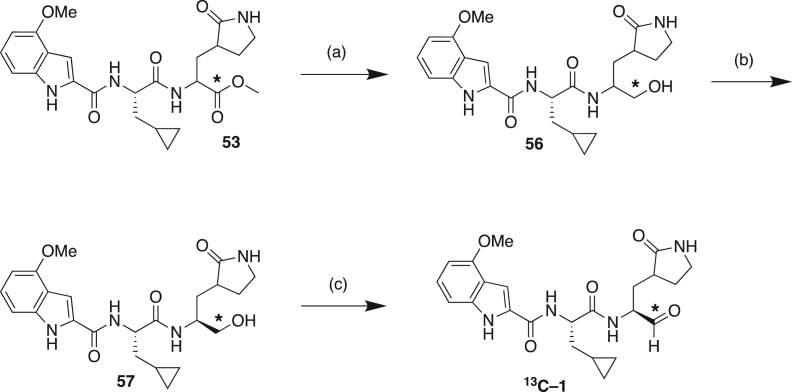

(a) LiBH4, THF, MeOH; (b) SFC; (c) Dess-Martin periodinane, DMSO, THF.

#### Preparation of *N*-((2*S*)-3-cyclopropyl-1-((1-hydroxy-3-(2-oxopyrrolidin-3-yl)propan-2-yl-1-^13^*C*)amino)-1-oxopropan-2-yl)-4-methoxy-1*H*-indole-2-carboxamide (56)

To a mixture of **53** (500 mg, 1.1 mmol) in THF (3.5 mL) was added lithium borohydride (2 M, 2.7 mL) dropwise. The mixture was stirred at 25°C for 0.5 h. Then MeOH (18 mL) was added dropwise to the mixture and the resulting mixture was stirred at 25°C for another 1.5 h. The resulting suspension was concentrated under reduced pressure. The white residue was diluted with MeOH (2.5 mL) and purified by Prep-HPLC under TFA condition to give **56** (370 mg, 78.7% yield) as a white solid.

#### Preparation of *N*-((*S*)-3-cyclopropyl-1-(((*S*)-1-hydroxy-3-((*S*)-2-oxopyrrolidin-3-yl)propan-2-yl-1-^13^*C*)amino)-1-oxopropan-2-yl)-4-methoxy-1*H*-indole-2-carboxamide (57)

The product **56** was dissolved in MeOH (1 mL) and separated by Chiral-SFC under neutral condition to give **57** (69 mg, 18.6% yield) as a white solid.

##### Chiral SFC method

Instrument: Waters SFC80Q Preparative SFC System. Column: Daicel ChiralPak IG (250∗30mm, 10 μm). Mobile phase: A for CO_2_ and B for EtOH. Gradient: B% = 55.00% isocratic elution mode. Flow rate: 80.00 g/min. Monitor wavelength: 220&254 nm. Column temperature: 40°C. System back pressure: 100 bar.

^**1**^**H NMR** 400 MHz, CD_3_OD

δ ppm 7.26 (d, *J* = 0.73 Hz, 1 H), 7.11–7.18 (m, 1 H), 7.02 (d, *J* = 8.31 Hz, 1 H), 6.51 (d, *J* = 7.70 Hz, 1 H), 4.59 (dd, *J* = 8.07, 6.48 Hz, 1 H), 3.97–4.06 (m, 1 H), 3.93 (s, 3 H), 3.64–3.78 (m, 1 H), 3.33–3.41 (m, 1 H), 3.19–3.29 (m, 2 H), 2.51–2.60 (m, 1 H), 2.28–2.38 (m, 1 H), 1.94–2.03 (m, 1 H), 1.67–1.86 (m, 3 H), 1.50–1.60 (m, 1 H), 0.80–0.91 (m, 1 H), 0.44–0.57 (m, 2 H), 0.12–0.26 (m, 2 H).

**LCMS (ESI+)**: m/z 444.5 (M + H)^+^.

#### Preparation of *N*-((*S*)-3-cyclopropyl-1-oxo-1-(((*S*)-1-oxo-3-((*S*)-2-oxopyrrolidin-3-yl)propan-2-yl-1-^13^*C*)amino)propan-2-yl)-4-methoxy-1*H*-indole-2-carboxamide (^13^C–1)

To a suspension of **57** (25 mg, 56.5 μmol) in THF (2 mL) was added Dess-Martin periodinane (40.7 mg, 96 μmol, 29.7 μL). The resulting off-white suspension was stirred at 20°C for 4 h. Then DMSO (0.2 mL) was added dropwise to the above suspension to make the clear solution and stirred for another 1 h. The resulting orange solution was concentrated by evaporation of THF and the residue was purified by prep-HPLC (column: Phenomenex Luna C18 100∗40mm∗5 μm; mobile phase: [H_2_O (0.1% TFA)-MCN]; gradient:15%–50% B over 8.0 min) to give ^**13**^**C-1** (12.7 mg, 51% yield) as a white solid.

^**1**^**H NMR** 400 MHz, DMSO-d_6_

δ ppm 11.59 (d, *J* = 1.50 Hz, 1 H), 9.15–9.67 (m, 1 H), 8.34–8.60 (m, 2 H), 7.59–7.80 (m, 1 H), 7.33–7.54 (m, 1 H), 6.95–7.14 (m, 2 H), 6.50 (d, *J* = 7.63 Hz, 1 H), 4.42–4.59 (m, 1 H), 4.22 (td, *J* = 11.51, 5.38 Hz, 1 H), 3.88 (s, 3 H), 3.02–3.17 (m, 2 H), 2.07–2.37 (m, 2 H), 1.34–1.96 (m, 5 H), 0.75–0.91 (m, 1 H), 0.32–0.48 (m, 2 H), 0.06–0.27 (m, 2 H).

### Quantification and statistical analysis

Details of for curve fitting for dose responses are provided in the method details sections above. The number of repeat measures for each measure are provided in Table 1. All data fitting for potency determination was carried out using Graphpad Prism 10.0.

## References

[1] Sun Z.G., Li Z.N., Zhu H.L. (2020). The Research Progress of DPP-4 Inhibitors. Mini Rev. Med. Chem..

[2] Williams B. (2016). Drug discovery in renin-angiotensin system intervention: past and future. Ther. Adv. Cardiovasc. Dis..

[3] Khadse A.N., Sharma M.K., Murumkar P.R., Rajput S.J., Yadav M.R. (2018). Advances in the Development of Novel Factor Xa Inhibitors: A Patent Review. Mini Rev. Med. Chem..

[4] de Leuw P., Stephan C. (2017). Protease inhibitors for the treatment of hepatitis C virus infection. GMS Infect. Dis..

[5] Ghosh A.K., Osswald H.L., Prato G. (2016). Recent Progress in the Development of HIV-1 Protease Inhibitors for the Treatment of HIV/AIDS. J. Med. Chem..

[6] Owen D.R., Allerton C.M.N., Anderson A.S., Aschenbrenner L., Avery M., Berritt S., Boras B., Cardin R.D., Carlo A., Coffman K.J. (2021). An oral SARS-CoV-2 M(pro) inhibitor clinical candidate for the treatment of COVID-19. Sci. Technol. Humanit..

[7] Abbenante G., Fairlie D.P. (2005). Protease inhibitors in the clinic. Med. Chem..

[8] Brogi S., Ibba R., Rossi S., Butini S., Calderone V., Gemma S., Campiani G. (2022). Covalent Reversible Inhibitors of Cysteine Proteases Containing the Nitrile Warhead: Recent Advancement in the Field of Viral and Parasitic Diseases. Molecules (Basel).

[9] Péter Ábrányi-Balogh G.M.K., Yao X. (2022). Developments in Organic Chemistry Advances in Chemical Proteomics.

[10] Bai B., Arutyunova E., Khan M.B., Lu J., Joyce M.A., Saffran H.A., Shields J.A., Kandadai A.S., Belovodskiy A., Hena M. (2021). Peptidomimetic nitrile warheads as SARS-CoV-2 3CL protease inhibitors. RSC Med. Chem..

[11] Hoffman R.L., Kania R.S., Br M.A., Davies J.F., Ferre R.A., Gajiwala K.S., He M., Hogan R.J., Kozminski K., Li L.Y. (2020). Discovery of Ketone-Based Covalent Inhibitors of Coronavirus 3CL Proteases for the Potential Therapeutic Treatment of COVID-19. J. Med. Chem..

[12] Schroder E., Phillips C., Garman E., Harlos K., Crawford C. (1993). X-ray crystallographic structure of a papain-leupeptin complex. FEBS Lett..

[13] Jamison D.A., Anand Narayanan S., Trovao N.S., Guarnieri J.W., Topper M.J., Moraes-Vieira P.M., Zaksas V., Singh K.K., Wurtele E.S., Beheshti A. (2022). A comprehensive SARS-CoV-2 and COVID-19 review, Part 1: Intracellular overdrive for SARS-CoV-2 infection. Eur. J. Hum. Genet..

[14] V'Kovski P., Kratzel A., Steiner S., Stalder H., Thiel V. (2021). Coronavirus biology and replication: implications for SARS-CoV-2. Nat. Rev. Microbiol..

[15] Chen P., Van Oers T.J., Arutyunova E., Fischer C., Wang C., Lamer T., van Belkum M.J., Young H.S., Vederas J.C., Lemieux M.J. (2024). A Structural Comparison of Oral SARS-CoV-2 Drug Candidate Ibuzatrelvir Complexed with the Main Protease (Mpro) of SARS-CoV-2 and MERS-CoV. JACS Au.

[16] Rhodin M.H.J., Reyes A.C., Balakrishnan A., Bisht N., Kelly N.M., Gibbons J.S., Lloyd J., Vaine M., Cressey T., Crepeau M. (2024). The small molecule inhibitor of SARS-CoV-2 3CLpro EDP-235 prevents viral replication and transmission in vivo. Nat. Commun..

[17] Xia B., Kang X. (2011). Activation and maturation of SARS-CoV main protease. Protein Cell.

[18] Aniana A., Nashed N.T., Ghirlando R., Coates L., Kneller D.W., Kovalevsky A., Louis J.M. (2023). Insights into the mechanism of SARS-CoV-2 main protease autocatalytic maturation from model precursors. Commun. Biol..

[19] Lee J., Worrall L.J., Vuckovic M., Rosell F.I., Gentile F., Ton A.T., Caveney N.A., Ban F., Cherkasov A., Paetzel M., Strynadka N.C.J. (2020). Crystallographic structure of wild-type SARS-CoV-2 main protease acyl-enzyme intermediate with physiological C-terminal autoprocessing site. Nat. Commun..

[20] Tomar S., Johnston M.L., St John S.E., Osswald H.L., Nyalapatla P.R., Paul L.N., Ghosh A.K., Denison M.R., Mesecar A.D. (2015). Ligand-induced dimerization of Middle East Respiratory Syndrome (MERS) coronavirus NSP5 protease (3CLpro): Implications for NSP5 regulation and the development of antivirals. J. Biol. Chem..

[21] Vuong W., Khan M.B., Fischer C., Arutyunova E., Lamer T., Shields J., Saffran H.A., McKay R.T., van Belkum M.J., Joyce M.A. (2020). Feline coronavirus drug inhibits the main protease of SARS-CoV-2 and blocks virus replication. Nat. Commun..

[22] Kovar P., Richardson P.L., Korepanova A., Afanador G.A., Stojkovic V., Li T., Schrimpf M.R., Ng T.I., Degoey D.A., Gopalakrishnan S.M., Chen J. (2024). Development of a sensitive high-throughput enzymatic assay capable of measuring sub-nanomolar inhibitors of SARS-CoV2 Mpro. SLAS Discov..

[23] Fornasier E., Fabbian S., Shehi H., Enderle J., Gatto B., Volpin D., Biondi B., Bellanda M., Giachin G., Sosic A., Battistutta R. (2024). Allostery in homodimeric SARS-CoV-2 main protease. Commun. Biol..

[24] Nashed N.T., Kneller D.W., Coates L., Ghirlando R., Aniana A., Kovalevsky A., Louis J.M. (2022). Autoprocessing and oxyanion loop reorganization upon GC373 and nirmatrelvir binding of monomeric SARS-CoV-2 main protease catalytic domain. Commun. Biol..

[25] Kovalevsky A., Aniana A., Coates L., Ghirlando R., Nashed N.T., Louis J.M. (2024). Visualizing the Active Site Oxyanion Loop Transition Upon Ensitrelvir Binding and Transient Dimerization of SARS-CoV-2 Main Protease. J. Mol. Biol..

[26] Aniana A., Nashed N.T., Ghirlando R., Drago V.N., Kovalevsky A., Louis J.M. (2024). Characterization of alternate encounter assemblies of SARS-CoV-2 main protease. J. Biol. Chem..

[27] Albani S., Costanzi E., Hoang G.L., Kuzikov M., Frings M., Ansari N., Demitri N., Nguyen T.T., Rizzi V., Schulz J.B. (2024). Unexpected Single-Ligand Occupancy and Negative Cooperativity in the SARS-CoV-2 Main Protease. J. Chem. Inf. Model..

[28] Shepherd T.A., Cox G.A., McKinney E., Tang J., Wakulchik M., Zimmerman R.E., Villarreal E.C. (1996). Small Peptidic Aldehyde Inhibitors of Human Rhinovirus 3C Protease. Bioorg. Med. Chem. Lett..

[29] Lewis C.A., Wolfenden R. (1977). Antiproteolytic aldehydes and ketones: substituent and secondary deuterium isotope effects on equilibrium addition of water and other nucleophiles. Biochemistry.

[30] Patten J.J., Keiser P.T., Morselli-Gysi D., Menichetti G., Mori H., Donahue C.J., Gan X., Valle I.D., Geoghegan-Barek K., Anantpadma M. (2022). Identification of potent inhibitors of SARS-CoV-2 infection by combined pharmacological evaluation and cellular network prioritization. iScience.

[31] Anand Balakrishnan, A.R., R. Shen, N. Bisht, Joyce S., R. Levene, N. McAllister, T. Cressey, N. Manalo, M.H. J. Rhodin, M. Vaine, et al. (2022). Molecular basis for antiviral action of EDP-235: A Potent and Selective SARS-CoV-2 3CLpro Inhibitor for the Treatment of COVID-19. held in Philadephia, PA USA

[32] Alberty R.A., Hammes G.G. (1958). Application of the Theory of Diffusion-controlled Reactions to Enzyme Kinetics. J. Phys. Chem..

[33] Pu F., Ugrin S.A., Radosevich A.J., Chang-Yen D., Sawicki J.W., Talaty N.N., Elsen N.L., Williams J.D. (2022). High-Throughput Intact Protein Analysis for Drug Discovery Using Infrared Matrix-Assisted Laser Desorption Electrospray Ionization Mass Spectrometry. Anal. Chem..

[34] Robertson A.J., Ying J., Bax A. (2022). NMR Observation of Sulfhydryl Signals in SARS-CoV-2 Main Protease Aids Structural Studies. Chembiochem.

[35] Williamson M.P. (2013). Using chemical shift perturbation to characterise ligand binding. Prog. Nucl. Magn. Reson. Spectrosc..

[36] Brisson J.R., Carey P.R., Storer A.C. (1986). Benzoylamidoacetonitrile is bound as a thioimidate in the active site of papain. J. Biol. Chem..

[37] Frankfater A., Kuppy T. (1981). Mechanism of association of N-acetyl-L-phenylalanylglycinal to papain. Biochemistry.

[38] Liang T.C., Abeles R.H. (1987). Inhibition of papain by nitriles: mechanistic studies using NMR and kinetic measurements. Arch. Biochem. Biophys..

[39] Malcolm B.A., Lowe C., Shechosky S., McKay R.T., Yang C.C., Shah V.J., Simon R.J., Vederas J.C., Santi D.V. (1995). Peptide aldehyde inhibitors of hepatitis A virus 3C proteinase. Biochemistry.

[40] Ortiz C., Tellier C., Williams H., Stolowich N.J., Scott A.I. (1991). Diastereotopic covalent binding of the natural inhibitor leupeptin to trypsin: detection of two interconverting hemiacetals by solution and solid-state NMR spectroscopy. Biochemistry.

[41] Tang C.P., Clark O., Ferrarone J.R., Campos C., Lalani A.S., Chodera J.D., Intlekofer A.M., Elemento O., Mellinghoff I.K. (2022). GCN2 kinase activation by ATP-competitive kinase inhibitors. Nat. Chem. Biol..

[42] Datta D., McClendon C.L., Jacobson M.P., Wells J.A. (2013). Substrate and inhibitor-induced dimerization and cooperativity in caspase-1 but not caspase-3. J. Biol. Chem..

[43] Zhou N.E., Tang S., Bian X., Parai M.K., Krieger I.V., Flores A., Jaiswal P.K., Bam R., Wood J.L., Shi Z. (2024). An oral non-covalent non-peptidic inhibitor of SARS-CoV-2 Mpro ameliorates viral replication and pathogenesis in vivo. Cell Rep..

[44] Delaglio F., Grzesiek S., Vuister G.W., Zhu G., Pfeifer J., Bax A. (1995). NMRPipe: a multidimensional spectral processing system based on UNIX pipes. J. Biomol. NMR.

[45] Vranken W.F., Boucher W., Stevens T.J., Fogh R.H., Pajon A., Llinas M., Ulrich E.L., Markley J.L., Ionides J., Laue E.D. (2005). The CCPN data model for NMR spectroscopy: development of a software pipeline. Proteins.

[46] Helmus J.J., Jaroniec C.P. (2013). Nmrglue: an open source Python package for the analysis of multidimensional NMR data. J. Biomol. NMR.

[47] Pu F., Knizner K.T., Robey M.T., Radosevich A.J., Ugrin S.A., Elsen N.L., Durbin K.R., Williams J.D. (2022). High-Throughput Deconvolution of Intact Protein Mass Spectra for the Screening of Covalent Inhibitors. J. Am. Soc. Mass Spectrom..

[48] Bernardes G.J.L., Chalker J.M., Errey J.C., Davis B.G. (2008). Facile conversion of cysteine and alkyl cysteines to dehydroalanine on protein surfaces: versatile and switchable access to functionalized proteins. J. Am. Chem. Soc..

[49] Lazzaro F., Crucianelli M., De Angelis F., Neri V., Saladino R. (2004). A novel oxidative side-chain transformation of α-amino acids and peptides by methyltrioxorhenium/H2O2 system. Tetrahedron Lett..

