# Supplementary material for: Inhibition of dimeric SARS-CoV-2 Mpro displays positive cooperativity and a mixture of covalent and non-covalent binding
Source: iScience. 2025 May 28;28(7):112773. doi: 10.1016/j.isci.2025.112773 (PMC12246642; doi:10.1016/j.isci.2025.112773)
Supplement: Document S1. Figures S1–S10 and Table S1 [file mmc1.pdf]

iScience, Volume *28*

Supplemental information

Inhibition of dimeric SARS-CoV-2 Mpro displays positive cooperativity and a mixture of covalent and non-covalent binding

Krishna M. Padmanabha Das, Jun Chen, Paul S. Charifson, Jeremy Green, Henry Tang, Sanjay Panchal, Fan Pu, Alla Korepanova, Abhinav Dubey, Gustavo Afanador, Vladimir Stojkovic, Boguslaw Nocek, Lance Bigelow, Sarah H. Stubbs, Robert A. Davey, David A. DeGoey, Haribabu Arthanari, and Mark N. Namchuk

SUPPLEMENTAL INFORMATION

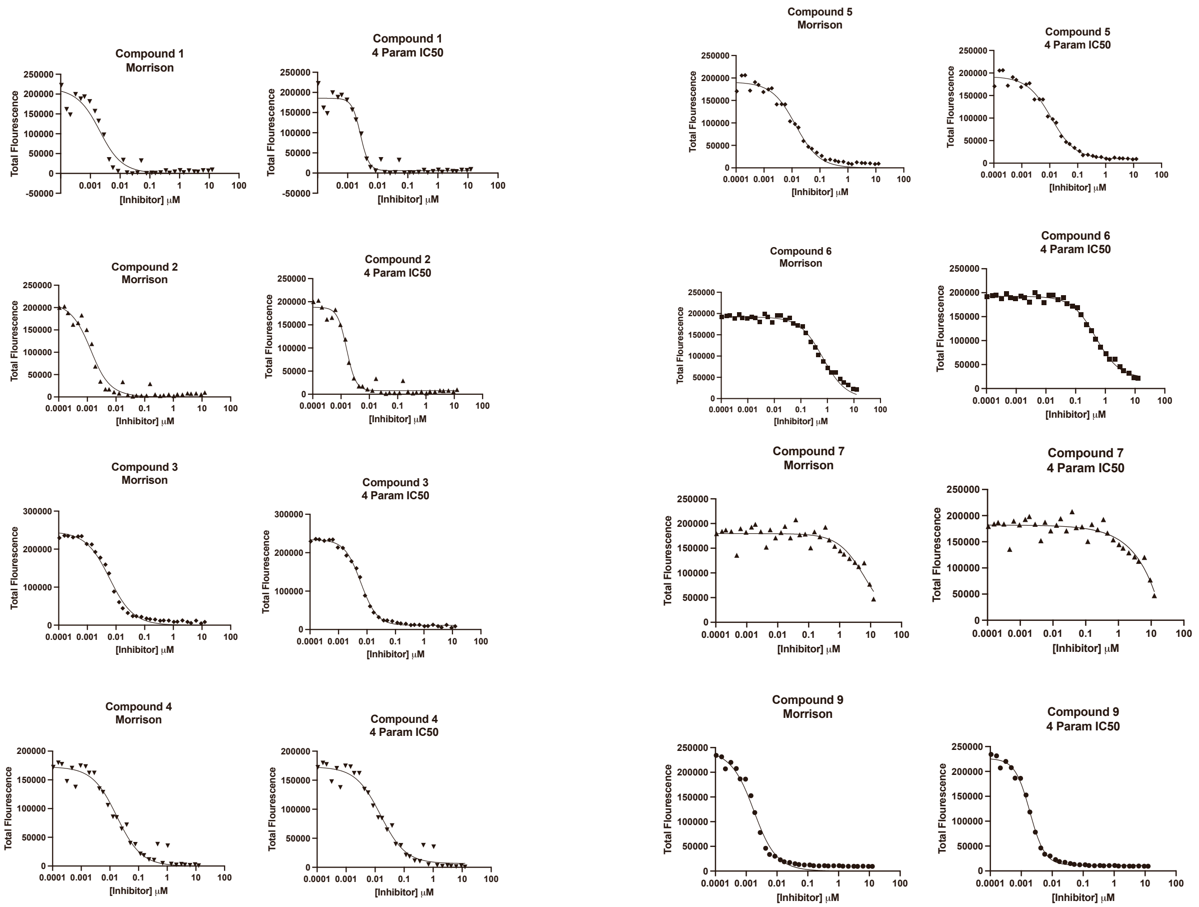

Figure S1. Related to Table 1. Representative examples of data for K_i determination. To determine compound potency, each compound was incubated with Mpro for 15 minutes before substrate addition, then fluorescent signal measured after an additional 20 minute. Given compound potency exceeded enzyme concentration for several compounds K_i values were determined using the Morrison tight binding equation (listed as Morrison above the plots). The identical data sets were refit to a 4 parameter IC_{50} fit to estimate Hill slope (4 Param IC_{50} above plots, see Table 1). All data sets were analyzed in Prism.

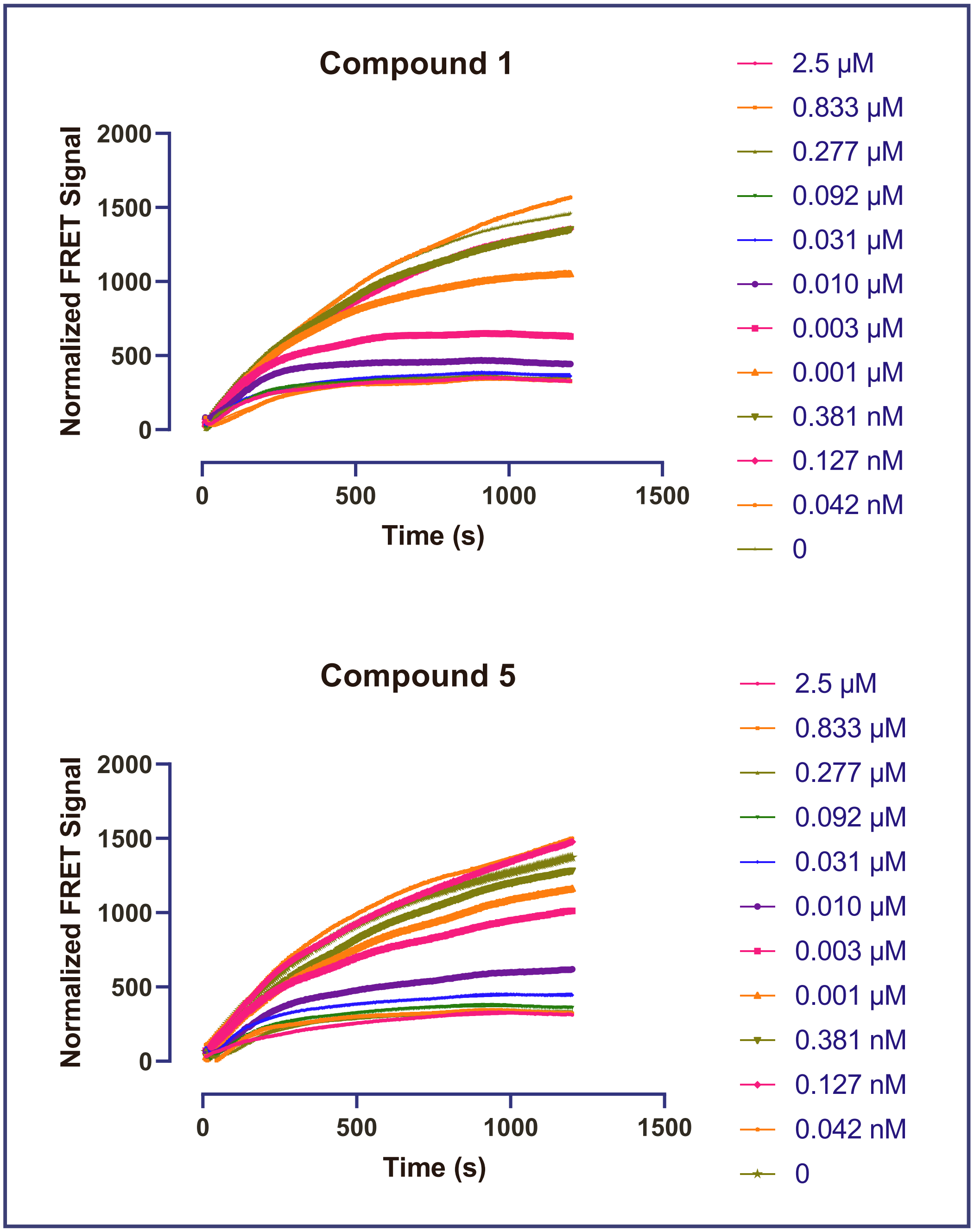

Figure S2. Related to main text, K_i determinations. Compound 1 or 5 plus 8 μM substrate were added simultaneously to a well containing 1 nM Mpro and the reaction followed for 1200 seconds will signal measured at 5 second intervals (Final substrate concentration 5 μM, final inhibitor concentrations as shown in Figure).

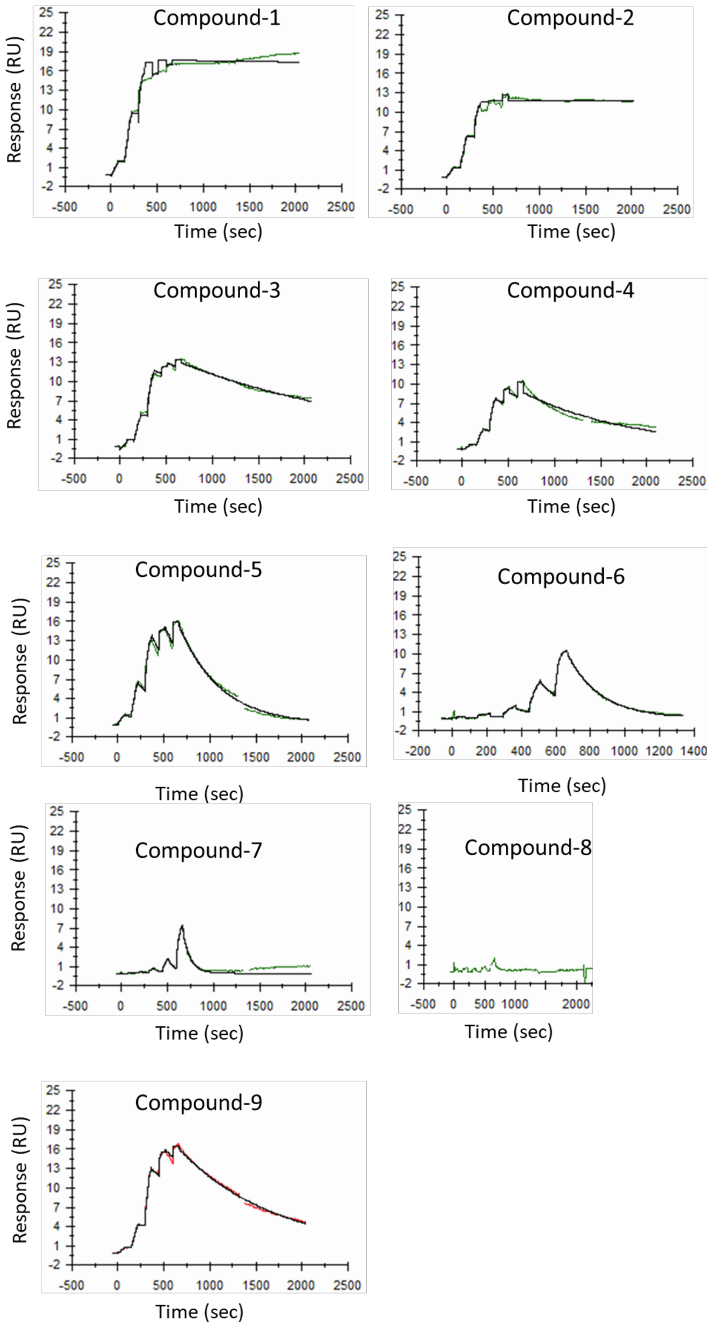

Figure S3 Related to Table 1. Representative SPR binding sensorgrams of compounds 1-9

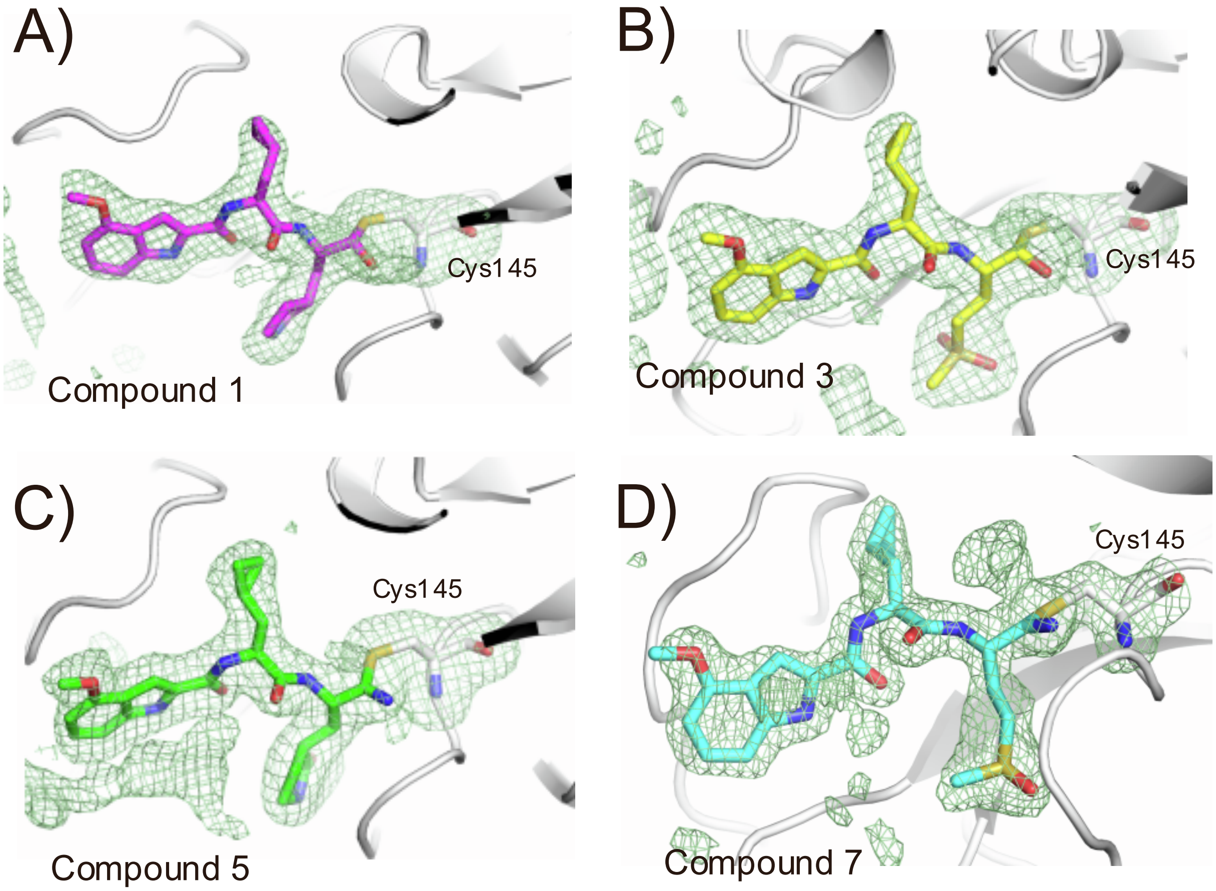

Figure S4. Related to Figure 1. Crystal structures of SARS-CoV-2 Mpro in complex with the four methoxy indoles. Polder maps (contoured to 3δ) generated over each ligand and including Cys145 show continuous omit density across the ligands and Cys145 (Panels A-D). In all cases the data support covalent binding of the compound with Cys145.

Aldehydes

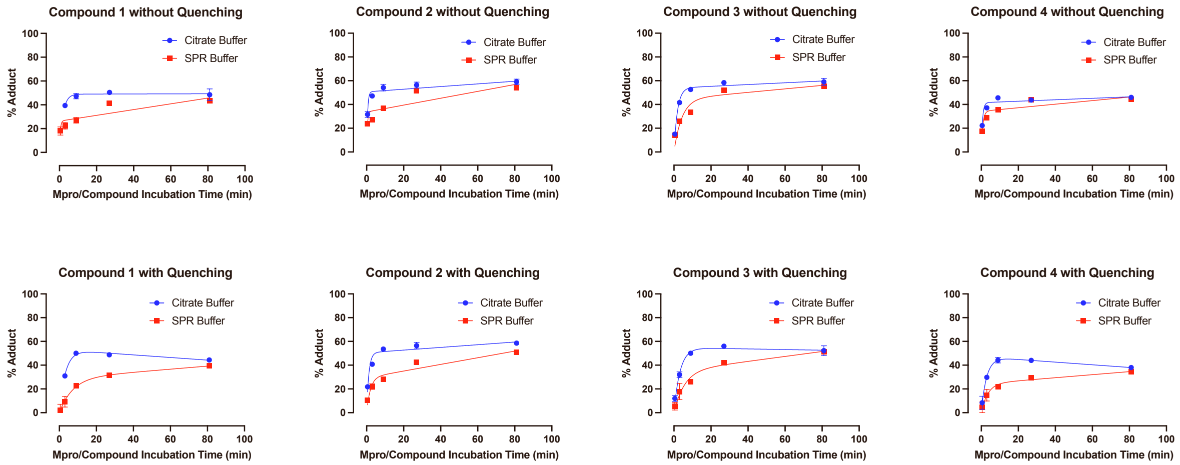

Nitriles

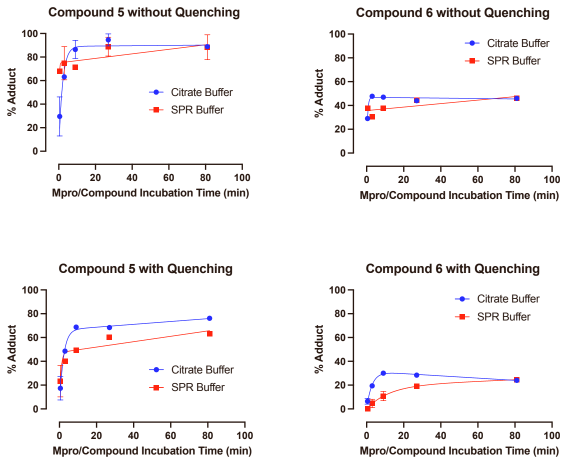

	kobs Min -1 (95 % CI) Quenched	
	Citrate	SPR
Compound 1	0.31 (0.2694 to 0.3510) *	0.13 (0.09094 to 0.2054)
Compound 2	0.85 (0.5590 to 1.304)	0.49 (0.2611 to 1.031)
Compound 3	0.30 (0.2430 to 0.3850)	0.18 (0.1099 to 0.3180)
Compound 4	0.35 (0.2834 to 0.4350)	0.29 (0.1687 to 0.5069)
Compound 5	0.47 (0.3769 to 0.6147)	1.2 (0.6073 to 2.111)
Compound 6	0.34 (0.2863 to 0.4271)	0.08 (0.02629 to 0.1692)

Figure S5. Related to Figure 2. Time course of the extent of covalent compound binding (adduct formation) assessed by MALDESI. The complexes were assessed after separation on a desalting column (without quenching) or after quenching the reaction with 200 μM compound 9, followed by desalting (with quenching). The percent adduct formation over time was fit to the slow binding equation to estimate the rate of conversion to the covalently bound species (k_{obs}). Time course values were best observed in the quenched conditions. All data were fit, but we note that the data in citrate buffer can only be estimated due to the lack of early timepoints in the plot*.

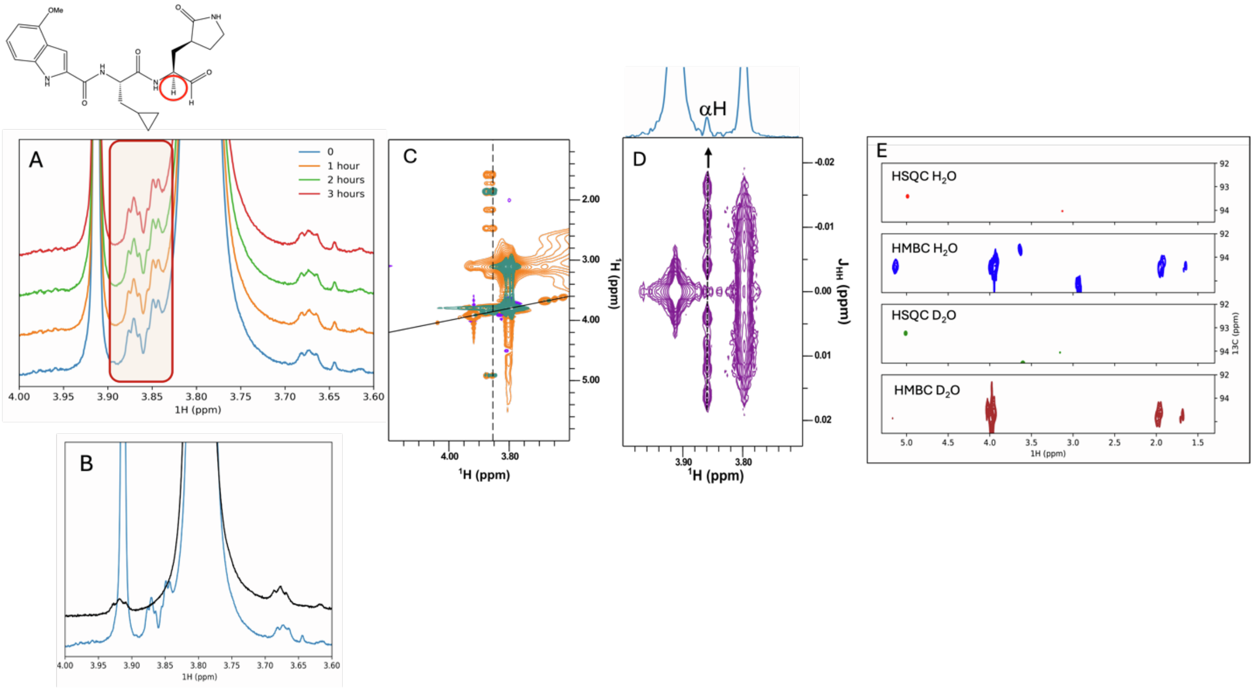

Figure S6. Related to STAR Methods. A) Overlay of the 1D proton spectrum of compound 1, highlighting the resonance corresponding to the alpha hydrogen at P1 (red circle), as a function of time after dilution into a D_2O buffer. No loss in signal was observed over time, consistent with no appreciable compound epimerization in solution. B) Overlay of the 1D –proton spectrum of the buffer alone (black) and the buffer with compound 1 (blue). C) Overlay of 2D TOCSY (orange) and 2D COSY (green) spectra highlighting the cross-peaks in the P1 αH region that assisted in the assignment of the αH resonance. D) 2D JRES spectrum of compound 1, focusing on the region corresponding to the P1 αH resonance. The 1D projection displayed at the top shows the αH resonance without homonuclear coupling. E) Two millimolar compound 1 is resuspended in either 100% D_2O or 100% H_2O, and HSQC and HMBC spectra were measured. HSQC shows a correlation between the ^{13}C labeled aldehyde carbon with the protons within 1 bond distance whereas the HMBC spectra show correlations up to 3 bonds away. Total incubation time 8 hrs**.**

```
         10         20         30         40         50         60
SGFRKMAFPS GKVEGCMVQV TCGTTTLNGL WLDDVVYCPR HVICTSEDML NPNYEDLLIR

         70         80         90        100        110        120
KSNHNFLVQA GNVQLRVIGH SMQNCVLKLK VDTANPKTPK YKFVRIQPGQ TFSVLACYNG

        130        140        150        160        170        180
SPSGVYQCAM RPNFTIKGSF LNGSAGSVGF NIDYDCVSFC YMHHMELPTG VHAGTDLEGN

        190        200        210        220        230        240
FYGPFVDRQT AQAAGTDTTI TVNVLAWLYA AVINGDRWFL NRFTTTLNDF NLVAMKYNYE

        250        260        270        280        290        300
PLTQDHVDIL GPLSAQTGIA VLDMCASLKE LLQNGMNGRT ILGSALLEDE FTPFDVVRQC

SGVTFQ
```

Assigned
No assignment available
The assignment could not be transferred

Figure S7. Related to Figure 3. The sequence of SARS-CoV-2 Mpro listing the residues that could be assigned, where no assignment was available or where the assignment could not be transferred. The chemical shifts were transferred from BMRB:51455, where near complete assignments were available. The residues for which assignments were not available (21/306) are marked in Blue. However, some of the assignments could not be transferred unambiguously (37/306) and they were omitted from the analysis (marked in red).

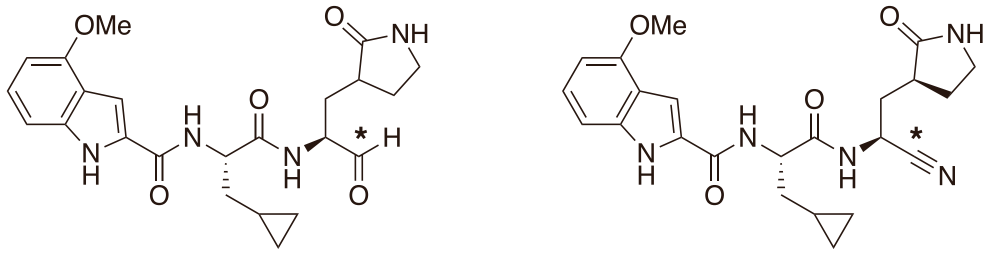

Figure S8. Related to Figure 5. The structures for compounds 1 and 5 with the asterisk denoting the site of ^{13}C labelling.

^{13}C NMR of Compound 1 in DMSO and SPR buffer: in DMSO and buffer

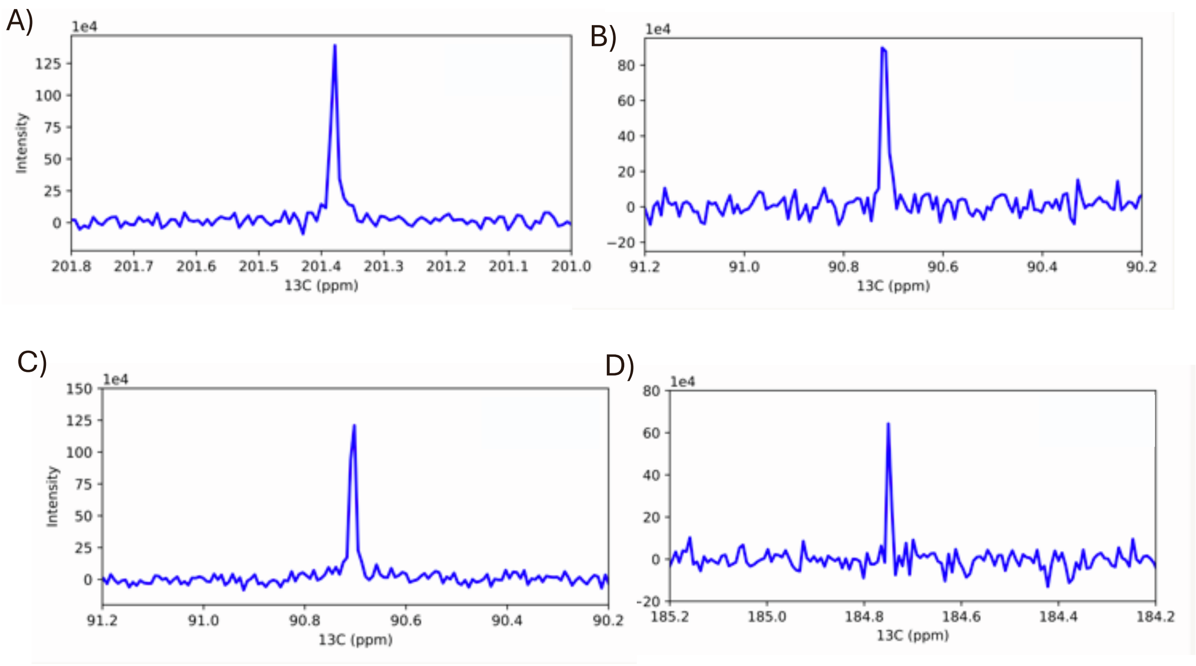

Figure S9. Related to Figure 5 A) 200 μM compound 1 in DMSO, and the aldehyde peak is at 201.4 ppm. B) 200 μM compound 1 in SPR buffer, the aldehyde hydrate peak is visible at 90.7 ppm. C) 200 μM compound 1 in SPR buffer adjusted to pH 6.0 where both the aldehyde (184.8 ppm) and diol peaks (90.7) are visible.

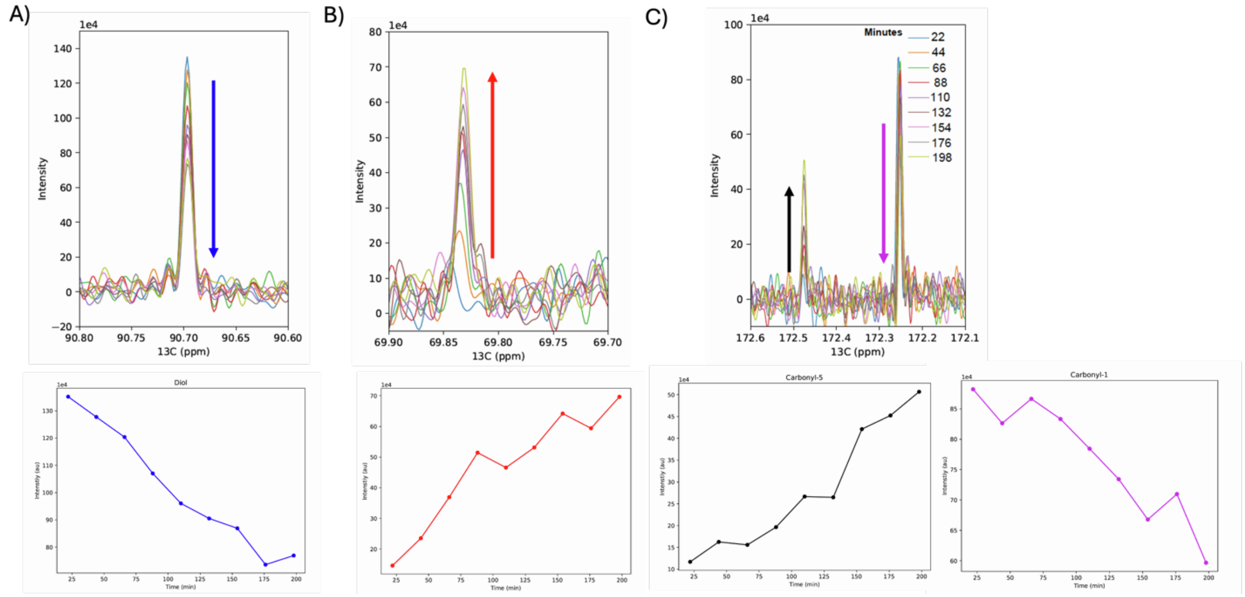

Figure S10. Related to Figure 5. 300 µM of compound 1 was incubated with 2 mM cysteine in SPR buffer, and ^{13}C- detected 1D experiments were acquired every 22 minutes. A) Shows the decrease in diol peak with time B) Increase in the thiol-diol peak with time, upon modification of the cysteine molecule, and C) shows a decrease in the carbonyl carbon peak observed due to natural abundance of ^{13}C (1.1 %) and a subsequent increase in the cysteine modified by compound 1. The plots below show the integrated peak volumes plotted against the reaction time showing the gradual increase or decrease in the individual populations.

Table S1. Data collection and refinement statistics. Related to Figure 1

	Mpro in complex with Compound 5	Mpro in complex with Compound 7	Mpro in complex with Compound 1	Mpro in complex with Compound 3
**Wavelength (Å)**	1.0000	1.0000	1.0000	0.9179
**Resolution range (Å)**	33.81 - 2.2 (2.279 - 2.2)	46.39 - 1.862 (1.928 - 1.862)	31.48 - 2.07 (2.144 - 2.07)	53.57 - 2.42 (2.507 - 2.42)
**Space group**	P 1 21 1	P 1 21 1	C 1 2 1	C 1 2 1
**Unit cell (a, b, c, α, β, γ)**	54.5 99.0 58.8 90 106.6 90	55.1 99.0 59.1 90 107.5 90	114.0 53.6 45.4 90 102.6 90	115.5 55.0 93.0 90 103.2 90
**Total reflections**	105508 (10671)	176748 (18152)	49609 (4451)	75699 (7876)
**Unique reflections**	30326 (3025)	50064 (4977)	15953 (1488)	21845 (2189)
**Multiplicity**	3.5 (3.5)	3.5 (3.6)	3.1 (2.9)	3.5 (3.6)
**Completeness (%)**	99.67 (99.74)	98.96 (98.42)	95.81 (91.97)	99.30 (100.00)
**Mean I/sigma(I)**	9.69 (2.32)	10.70 (2.34)	6.26 (2.63)	4.88 (2.08)
**Wilson B-factor ($Å^2$)**	41.01	27.74	38.95	46.99
**R-merge**	0.07848 (0.5213)	0.06748 (0.4901)	0.1185 (0.4511)	0.1767 (0.4819)
**R-meas**	0.09296 (0.6146)	0.07976 (0.574)	0.141 (0.5516)	0.2097 (0.5681)
**R-pim**	0.04943 (0.3233)	0.04218 (0.2973)	0.07566 (0.3137)	0.1119 (0.2984)
**$CC_{1/2}$**	0.995 (0.839)	0.997 (0.88)	0.98 (0.837)	0.958 (0.828)
**CC***	0.999 (0.955)	0.999 (0.968)	0.995 (0.955)	0.989 (0.952)
**Reflections used in refinement**	30310 (3022)	50045 (4974)	15763 (1489)	21839 (2190)
**Reflections used for R-free**	1481 (165)	2454 (247)	719 (72)	1120 (120)
**R-work**	0.2036 (0.2981)	0.1630 (0.2323)	0.2126 (0.3691)	0.2041 (0.2692)
**R-free**	0.2294 (0.3445)	0.1944 (0.2483)	0.2382 (0.4537)	0.2325 (0.3331)
**CC(work)**	0.955 (0.831)	0.967 (0.929)	0.947 (0.727)	0.950 (0.868)
**CC(free)**	0.946 (0.731)	0.953 (0.897)	0.936 (0.686)	0.962 (0.783)
**Number of non-hydrogen atoms**	4852	5043	2506	4827
**macromolecules**	4687	4689	2363	4666
**ligands**	121	116	61	118
**solvent**	100	292	111	99

**Protein residues**	605	602	303	602
**RMS(bonds) (Å)**	0.011	0.007	0.004	0.008
**RMS(angles) (°)**	1.58	1.01	0.73	1.24
**Ramachandran favored (%)**	97.67	98.66	97.67	97.99
**Ramachandran allowed (%)**	2.00	1.34	1.99	2.01
**Ramachandran outliers (%)**	0.33	0.00	0.33	0.00
**Rotamer outliers (%)**	1.54	0.19	2.29	2.12
**Clashscore**	5.14	3.21	1.70	7.21
**Average B-factor ($Å^2$)**	53.02	36.35	51.17	55.35
**macromolecules**	53.11	35.97	51.32	55.55
**ligands**	53.01	35.53	44.81	48.92
**solvent**	48.91	42.59	49.82	49.82
**Number of TLS groups**	14	8	3	12

Statistics for the highest-resolution shell are shown in parentheses.
